# Comparative effectiveness of non-pharmacological interventions on anxiety, depression, and quality of life in patients with epilepsy: a systematic review and network meta-analysis

**DOI:** 10.3389/fpsyt.2025.1624276

**Published:** 2025-07-30

**Authors:** Haoran Luo, Xianming Ding, Junyu Zhang, Ningkun Xiao

**Affiliations:** ^1^ Institute of Physical Education, Sports and Tourism, St. Petersburg Great Technical University, St. Petersburg, Russia; ^2^ College of Sports Science, Jishou University, Jishou, China; ^3^ School of Competitive Sports, Beijing Sport University, Beijing, China; ^4^ Laboratory for Brain and Neurocognitive Development, Department of Psychology, Institution of Humanities, Ural Federal University, Yekaterinburg, Russia; ^5^ Department of Immunochemistry, Institution of Chemical Engineering, Ural Federal University, Yekaterinburg, Russia

**Keywords:** non-pharmacological intervention, epilepsy, anxiety, depression, quality of life, systematic review, network meta-analysis

## Abstract

**Introduction:**

Epilepsy is a persistent neurological condition featuring abnormal electrical activity in the brain. Beyond its neurological symptoms, it is frequently associated with comorbid anxiety and depression, which significantly impair patients’ quality of life (QoL). Cognitive therapy (CBT), psychotherapy, and self-management (SM) have been substantiated through research to be significantly effective in alleviating psychological distress and enhancing quality of life. However, comprehensive comparisons of these diverse interventions remain scarce, highlighting a critical gap in the literature.

**Objective:**

This study aims to compare, through randomized controlled trials, the effects of different non-pharmacological interventions versus controls on anxiety, depression, and quality of life in patients with epilepsy.

**Data sources:**

A systematic search was conducted in five electronic databases: Cochrane Library, PsycInfo, PubMed, Web of Science and the Embase, covering studies published up to March 19, 2025. The search strategy included terms such as “exercise,” “mind-body exercise,” “cognitive-behavioral therapy,” “psychotherapy,” “epilepsy,” “anxiety,” “depression,” and “quality of life.”

**Study selection:**

Only English-language randomized controlled trials (RCTs) were included. Eligible studies examined the effects of non-pharmacological interventions on anxiety, depression, and QoL in patients clinically diagnosed with epilepsy. There were no restrictions on participants’ age or gender. Control conditions included standard care, placebo, wait-list, or alternative non-pharmacological interventions.

**Data extraction and synthesis:**

Three authors independently screened studies and extracted data. A frequentist random-effects network meta-analysis was conducted to calculate standardized mean differences (SMDs) along with 95% confidence intervals (CIs). The relative efficacy of interventions was ranked using the surface under the cumulative ranking curve (SUCRA). The analysis was conducted in accordance with the PRISMA-NMA reporting guidelines.

**Main outcomes and measures:**

Primary outcomes included changes in anxiety, depression, and QoL. Outcomes were assessed using validated psychological scales across studies.

**Results:**

Fifty-eight RCTs encompassing 6,541 participants across 20 countries or regions were included. Compared to control groups(CON), enhanced education therapy (EET) and psychotherapy (PT) were significantly more effective in reducing anxiety symptoms. Psychotherapy also demonstrated notable efficacy in alleviating depressive symptoms. For QoL improvement, Cognitive-behavioral therapy (CBT), mind-body therapies (MBT), Psychotherapy (PT), and enhanced care (EC) all showed significant advantages over controls. SUCRA rankings suggested that Enhanced education therapy (EET), Psychotherapy (PT), and Enhanced care (EC) were the most effective interventions for improving anxiety, depression, and Quality of Life (QoL), respectively. Subgroup analyses further suggest that enhanced education therapy and CBT may be more beneficial for minors in reducing anxiety and improving QoL, respectively, while psychotherapy shows consistent superiority in adults for both anxiety and depression.

**Conclusion:**

This network meta-analysis of 58 RCTs highlights the comparative benefits of multiple non-pharmacological strategies in improving mental health and QoL in patients with epilepsy. Interventions such as psychotherapy, CBT, and enhanced education appear particularly effective across psychological domains. These findings support the integration of tailored, non-pharmacological approaches into routine care for epilepsy and underscore the need for clinicians and policymakers to prioritize mental health alongside seizure control.

**Systematic review registration:**

https://www.crd.york.ac.uk/PROSPERO, identifier CRD420251015149.

## Introduction

Epilepsy is a persistent neurological condition marked by repeated seizures resulting from abnormal electrical discharges in the brain. It affects individuals across all age groups and remains one of the most prevalent neurological conditions globally, with an estimated 50 million people affected—a number that continues to rise worldwide (1, 2). Beyond its neurological manifestations, epilepsy is increasingly recognized as a condition influenced by a complex interplay of neurobiological, cognitive, psychological, and social factors (3, 4), with a disproportionately higher burden observed among children, older adults, and individuals in low-income populations (2). Clinically, seizures may present with a range of symptoms, including loss of consciousness, abnormal motor activity, sensory disturbances (e.g., visual, auditory, gustatory), emotional dysregulation, and impaired cognitive functioning, all of which can contribute to physical injury or even mortality (5). Unfortunately, people living with epilepsy (PWE) often experience social stigma, discrimination, and psychological distress, placing a substantial emotional and social burden on both patients and their families (6, 7). Approximately 50%–60% of individuals with chronic epilepsy report significant mood disorders, particularly anxiety and depression, both of which are strongly associated with reduced QoL (8, 9).

Given the profound impact of epilepsy on psychological well-being and long-term health outcomes, a wide range of therapeutic approaches has been explored. While pharmacological treatments remain the standard of care, their adverse effects—including increased teratogenic risks (10), dermatological and neurological side effects, and hepatobiliary complications—raise significant concerns (11, 12). Additionally, some antiseizure medications may exacerbate psychiatric symptoms, such as anxiety and cognitive dysfunction (12).

In recent years, non-pharmacological interventions (NPIs) have gained traction as promising complementary strategies to improve seizure control, QoL, and mental health in PWE (13).These interventions include vagus nerve stimulation, physical exercise, and mind-body practices (14), and growing evidence supports their positive effects on anxiety, depression, and overall well-being (15–18). Due to their favorable safety profiles, low side-effect burdens, and high acceptability, non-pharmacological treatments are now increasingly recommended as adjunctive therapies for the psychosocial management of epilepsy (19).

For instance, yoga has been associated with increased GABAergic activity and mood enhancement; cognitive behavioral therapy (CBT) is effective in managing psychiatric symptoms and reducing seizure frequency; lifestyle-based self-management and dietary interventions may decrease epilepsy-related complications; and regular exercise has been shown to improve memory, attention, executive function, and psychosocial outcomes (20). Several systematic reviews and conventional meta-analyses have assessed the therapeutic effects of individual non-pharmacological strategies in epilepsy populations (21–25). Collectively, they suggest that these interventions are cost-effective, accessible, and clinically beneficial in alleviating psychiatric symptoms and enhancing QoL (14).

However, most previous reviews have been limited in scope, focusing on either a single non-pharmacological modality (e.g., yoga or CBT) or a specific outcome (e.g., depression alone), without providing a comparative framework across diverse interventions or mental health dimensions. To our knowledge, this is the first network meta-analysis to comprehensively synthesize the evidence on multiple non-pharmacological interventions—including cognitive-behavioral therapy (CBT), psychotherapy, mind–body therapies, enhanced education, self-management, and neuromodulation—and evaluate their effects on anxiety, depression, and quality of life in patients with epilepsy.

In contrast to traditional meta-analyses that rely solely on pairwise comparisons derived from direct evidence (21, 26, 27), network meta-analysis (NMA) allows the integration of both direct and indirect evidence to simultaneously estimate and rank the relative effectiveness of three or more interventions (28–30). Even in the absence of head-to-head trials, NMA enables robust comparison and ranking through probabilistic modeling (e.g., SUCRA), which provides a more informative basis for clinical decision-making.

Therefore, the present study aimed to conduct a comprehensive NMA to evaluate and compare the efficacy of multiple non-pharmacological interventions in improving anxiety, depression, and QoL in patients with epilepsy. By systematically assessing and ranking the relative benefits of these interventions, our findings aim to fill existing gaps in the literature and provide clinicians with evidence-based recommendations for personalized, non-pharmacological treatment strategies for epilepsy.

## Methods

This study has been registered in the PROSPERO database (Registration ID: CRD420251015149) and strictly followed the guidelines of the Preferred Reporting Items for Systematic Reviews and Meta-Analyses (PRISMA 2020) and the PRISMA Extension for Network Meta-Analyses (PRISMA-NMA) to ensure transparency and rigor in methodology (31, 32).

### Search strategy

We systematically searched five major databases—Cochrane Central Register of Controlled Trials (CENTRAL), PsycInfo, PubMed, Web of Science, and Embase—from their inception to March 19, 2025, to identify randomized controlled trials (RCTs) examining the effects of non-pharmacological interventions in patients with epilepsy. The search was independently conducted by three reviewers (Luo, Ding, and Zhang). During this process, all disagreements among the three researchers were resolved by consulting a fourth researcher (Xiao). To ensure the completeness of our study. In addition, we reviewed the references of relevant meta-analyses to identify potentially eligible studies, thereby ensuring the comprehensiveness of our research.The details of search strategy is provided in Appendix 1.

### Inclusion criteria

Inclusion was determined based on the following criteria:

1. Participants had a clinically confirmed diagnosis of epilepsy.
2. Interventions that do not include drugs or other active substances.Such as, cognitive-behavioral therapy and exercise.
3. Primary outcomes included anxiety, depression, or quality of life (QoL), assessed using validated measurement tools.
4. Randomized controlled trial (RCT).
5. At least one post-intervention assessment reported at any follow-up time point, with no strict duration cutoff.
6. Articles were published in English.

Exclusion was determined based on the following criteria:

Studies were excluded if they:

1. Did not include participants with clinically confirmed epilepsy.
2. Employed pharmacological interventions as the primary exposure.
3. Did not assess outcomes related to anxiety, depression, or QoL, or used unvalidated instruments.
4. Were not RCTs (e.g., observational studies, case reports, quasi-experiments).
5. Had duplicate or incomplete data, lacked full text, or failed to report extractable outcome data.

### Study screening

All retrieved records were imported into EndNote 21.5 to automatically remove duplicates. The initial screening of titles and abstracts was independently conducted by three researchers (Luo, Ding, and Zhang). Any studies that appeared to meet the eligibility criteria were retained for full-text screening. Final inclusion decisions were reached by consensus during virtual meetings.

### Data extraction

All researchers independently extracted data from the included articles using a pre-designed standardized form. The table includes the following information: (1) Study characteristics (author, publication date, country/region); (2) Participant characteristics (sample size, age, sex); (3) Intervention and control conditions (type, duration, frequency); (4) Risk of bias assessment; (5) Outcome measures (type of validated scale used for anxiety, depression, and QoL). To ensure comparability of intervention effects, the primary outcome data extracted for this analysis were from the first post-intervention assessment time point reported in each included study. All discrepancies were discussed and resolved through consensus after a full-text review.

### Intervention classification and justification

In this study, we adopted a specific operational definition of non-pharmacological interventions (NPIs) to ensure conceptual clarity and analytic consistency. We defined NPIs as structured therapeutic strategies that do not involve pharmacological agents or the ingestion of biologically active substances. This definition aligns with previous reviews and focuses on interventions whose therapeutic effects are mediated through behavioral, psychological, or neuromodulatory mechanisms) (33, 34).

Accordingly, we included interventions such as cognitive behavioral therapy (CBT), mind–body therapies (e.g., mindfulness, yoga, tai chi), traditional exercise programs, self-management strategies, psychoeducational interventions, relaxation techniques, neuromodulation (e.g., transcranial magnetic stimulation), and enhanced care models (e.g., collaborative care, case management). While dietary therapies—such as the ketogenic diet—are commonly categorized as non-pharmacological in broader clinical contexts, we excluded them from this review because their mechanism of action depends primarily on the biochemical properties of ingested substances. Their inclusion could have introduced conceptual and methodological heterogeneity.

To facilitate network connectivity and ensure conceptual consistency, we categorized interventions into distinct nodes based on shared therapeutic principles and mechanisms of action. Specifically, yoga, mindfulness-based breathing exercises, and meditation were grouped under the node of Mind–Body Therapy (MBT). This decision was informed by prior meta-analyses and theoretical frameworks that consider these modalities as integrated strategies aimed at regulating physiological states (e.g., autonomic function, respiration) to promote psychological well-being (35). From a neurobiological perspective, these interventions are known to activate parasympathetic pathways, increase γ-aminobutyric acid (GABA) levels, and attenuate stress responses, thereby contributing to improvements in anxiety, depression, and quality of life. Moreover, these practices are often applied in combination in both clinical settings and research trials, further supporting their classification under a unified MBT node (36). Detailed intervention definitions and classification criteria are provided in **Supplementary Methods 4**.

### Risk of bias and quality assessment

Risk of bias was independently assessed for all included studies by two reviewers (Luo and Ding) using the Cochrane Risk of Bias 2.0 (RoB 2) tool. The tool evaluates five domains: (1) the randomization process; (2) deviations from intended interventions; (3) missing outcome data; (4) measurement of the outcome; and (5) selection of the reported result. Each domain was rated as “low risk,” “some concerns,” or “high risk.” The overall risk of bias for each study was determined according to RoB 2 guidelines: (1) “high risk” if any domain was rated high risk; (2) “some concerns” if one or more domains were rated as such but none were high risk; and (3) “low risk” if all domains were rated low risk. Disagreements were resolved by a third reviewer (Xiao).

### Data synthesis and statistical analysis

All analyses were performed using Stata 17.0. Network meta-analysis (NMA) integrates direct and indirect comparisons across multiple interventions. The evidence network diagram serves as a visual representation of the comparative structure. In each node represents a distinct intervention, and the size of the node is proportional to the cumulative sample size across all studies involving that intervention. The edges (connections) between nodes represent direct comparisons reported in the literature. The thickness of each edge reflects the number of studies providing direct comparisons for that treatment pair—thicker lines indicate more robust direct evidence, while thinner lines suggest limited direct data and greater reliance on indirect evidence.

Standardized mean differences (SMDs) with 95% confidence intervals (CIs) were used to synthesize continuous outcomes, accounting for heterogeneity in measurement scales. Statistical significance was set at α = 0.05.

A frequentist random-effects network meta-analysis (NMA) was conducted to simultaneously compare the effectiveness of multiple non-pharmacological interventions. We first examined the topological structure of the intervention network to assess its geometry. For open-loop networks, a consistency model was applied directly. For closed-loop networks, inconsistency was evaluated using loop-specific inconsistency tests and node-splitting models, which compare the effect estimates derived from direct versus indirect evidence. A P-value > 0.05 in the node-splitting model indicates acceptable agreement between direct and indirect comparisons, supporting the use of a consistency model. In contrast, a P < 0.05 suggests significant inconsistency, prompting the use of an inconsistency model and further exploration through subgroup analyses. Additionally, local inconsistency was assessed by calculating the inconsistency factor (IF) for each closed loop. An IF 95% CI that includes zero indicates consistency within the loop.

The comparative effectiveness of interventions was ranked using the surface under the cumulative ranking curve (SUCRA), which quantifies the likelihood that each intervention is the most effective (range: 0% to 100%).

## Results

### Literature selection and characteristics

**Figure 1:**
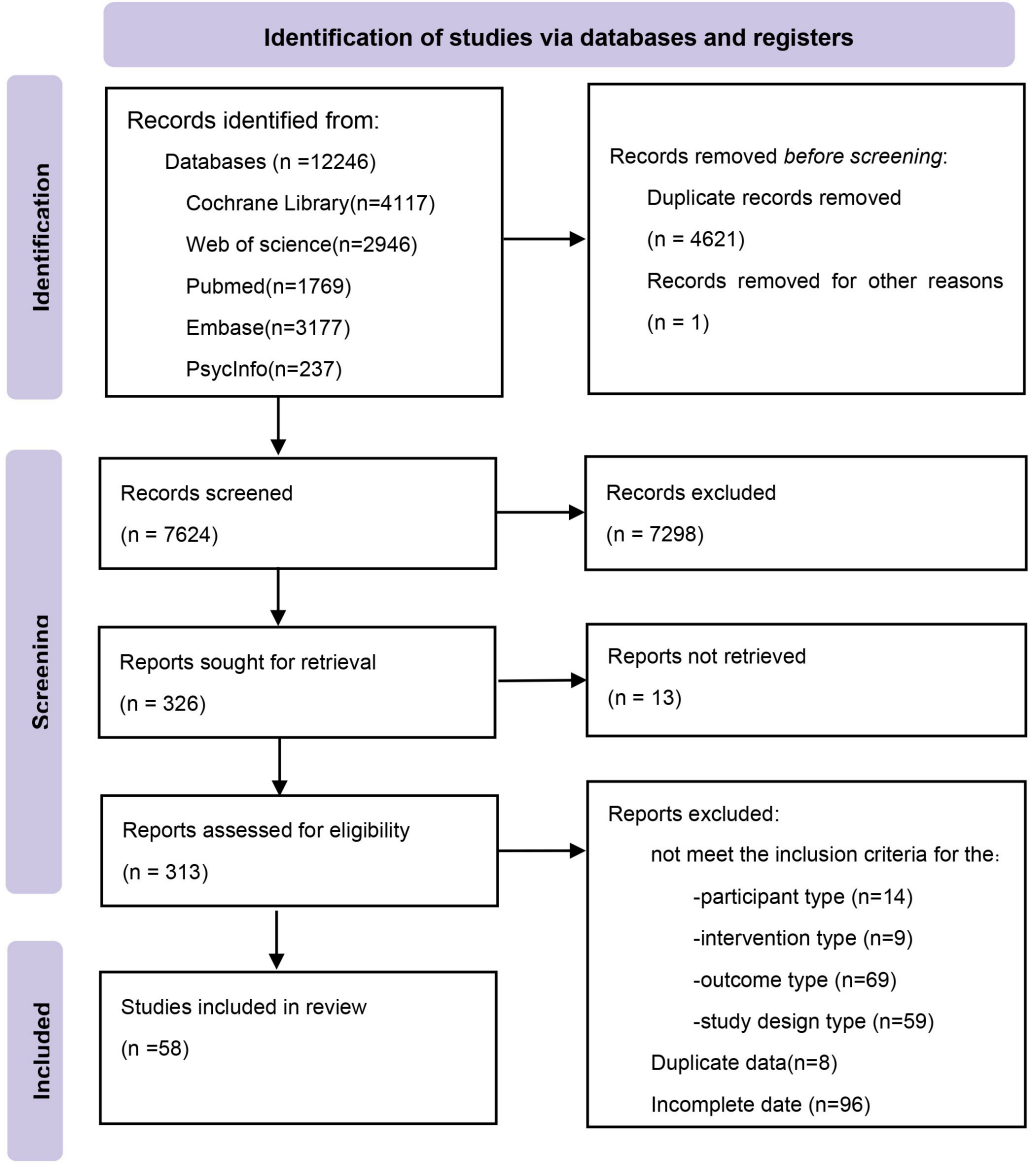
Flow diagram of systematic literature search.

We identified 12,246 potentially relevant citations from the databases. After removing duplicates, we screened the titles and abstracts. Finally, 58 eligible studies were included (**Figure 1**). The included studies spanned 20 countries or regions and involved a total of 6,541 participants. The mean age of participants ranged from 4.0 years (SD = 1.4) (37) to 72.4 years (SD = 20.1) (38). Sample sizes ranged from 20 participants (39, 40) to 660 participants (38). A total of 13 distinct non-pharmacological interventions were included, with intervention durations varying from a single session (41) to 12 months (42). The network meta-analysis (NMA) included 13 interventions for the outcome of depression and 12 interventions for both anxiety and quality of life (QoL) (see **Supplementary Table S1** for details).

### Quality assessment

Using the RoB 2 tool, we assessed the methodological quality of the 58 included RCTs. In total, 19 studies were judged as having low risk of bias, 34 showed some concerns, and 5 were classified as high risk.

In the randomization process domain, most studies clearly described sequence generation and allocation concealment; 18 were rated “some concerns” due to incomplete reporting. In the deviations from intended interventions domain, 27 studies lacked detail on analytic approaches (e.g., intention-to-treat), while 3 studies exhibited significant deviations and were rated high risk. In the missing outcome data domain, 21 studies had moderate or imbalanced attrition (“some concerns”), and 4 had high dropout rates (“high risk”). In the measurement of the outcome domain, 2 studies used invalid or poorly reported outcome measures, leading to high-risk judgments. In the selection of the reported result domain, 7 studies lacked access to pre-registered protocols and were judged as “some concerns.” Detailed domain-level assessments for each study are provided in **Supplementary Table S2**.

### Evidence network and coherence analysis

**Figure 2:**
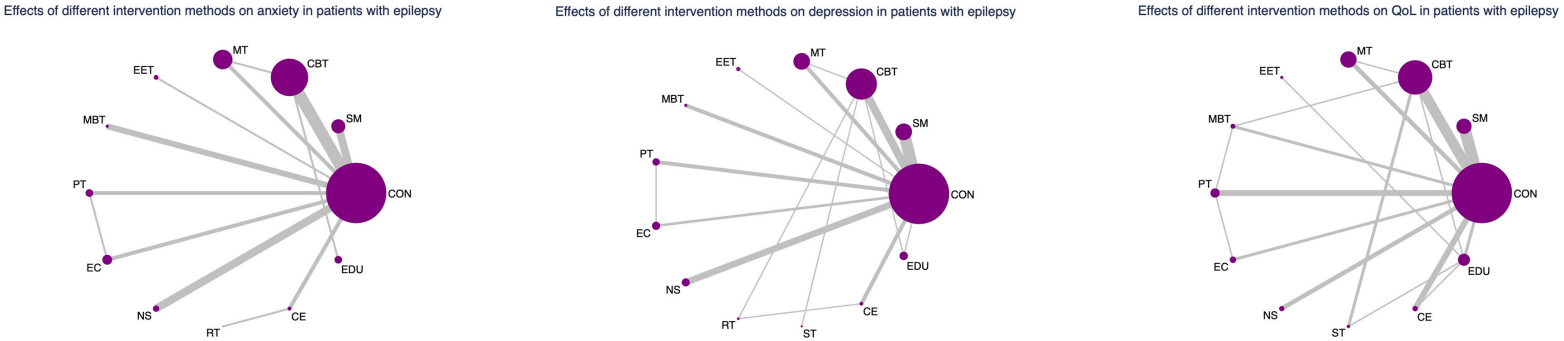
Network evidence graph for anxiety, depression and QoL.

**Figure 3:**
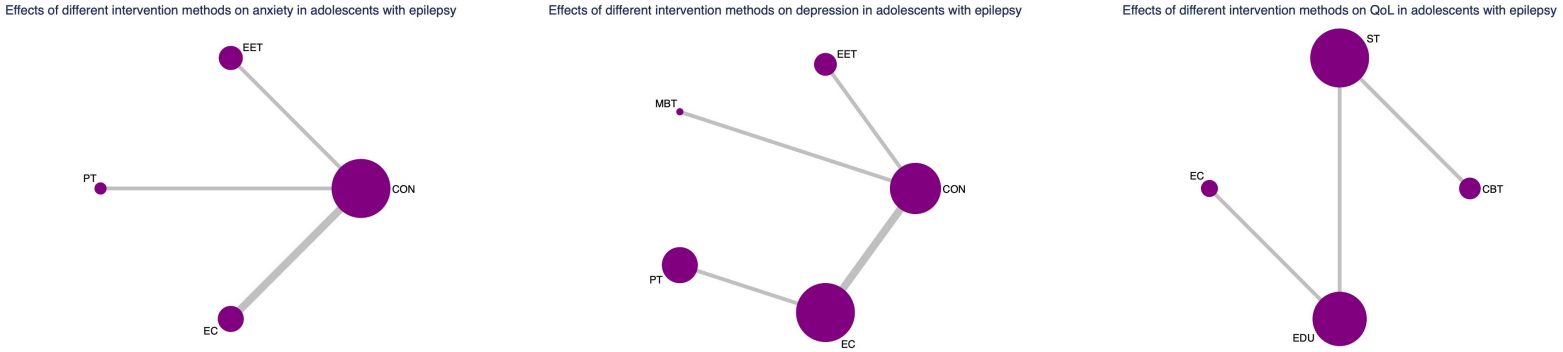
Network evidence graph for anxiety, depression and QoL in adolescents.

**Figure 4:**
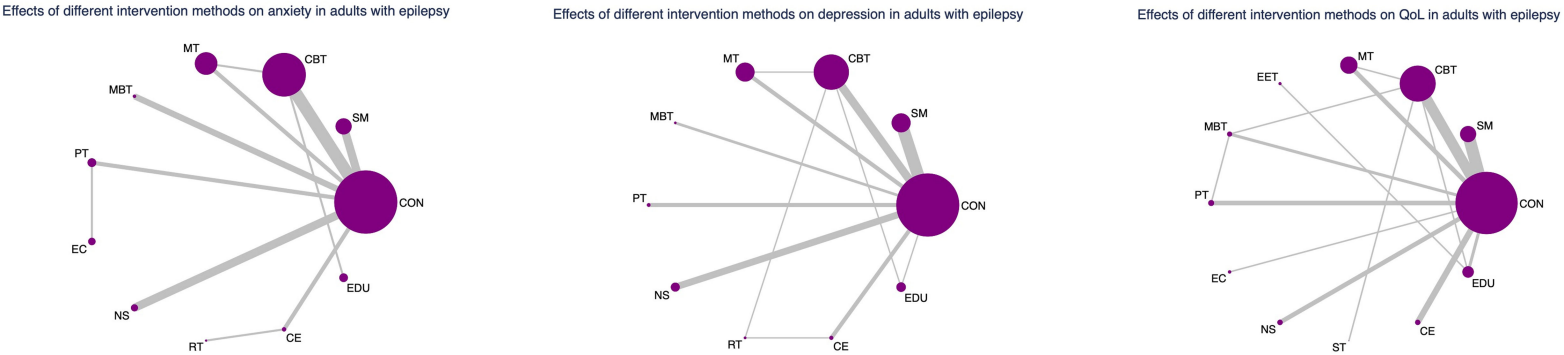
Network evidence graph for anxiety, depression and QoL in adults. CON, Control group; SM, Self-management; CBT, Cognitive-behavioral therapy; MT, Multi-component intervention; EET, Enhanced education therapy; MBT, Mind-body therapy; PT, Psychotherapy; EC, Enhanced care; NS, Neurostimulation; RT, Relaxation therapy; ST, Supportive therapy; CE, Conventional Exercise; EDU, Education.

As depicted in **Figures 2**–**4**, the results of the Network Meta-Analysis (NMA) are illustrated. In the figure, each point represents a different intervention, and the number of points indicates the number of interventions included in the analysis for that particular outcome measure. The connections between the points signify direct comparisons between the respective interventions, with the thickness of the lines representing the number and frequency of direct comparisons between those interventions.

Furthermore, we examined the topological structure of the network and assessed the consistency and inconsistency within the network, particularly in the presence of closed-loop structures, through loop inconsistency tests, overall inconsistency tests, and local inconsistency tests. The results of these tests showed that all p-values were greater than 0.05, indicating that the consistency of the studies was acceptable.

### Effects of non-pharmacological interventions on anxiety

A network meta-analysis of 28 RCTs revealed that enhanced education therapy (EET) and psychotherapy (PT) were significantly more effective than the control group in reducing anxiety symptoms in patients with epilepsy, with effect sizes of (SMD: −1.80,95% CI: −3.31 to −0.30) and (SMD:−1.41,95% CI: −2.44 to −0.39),respectively. The remaining nine interventions—including self-management (SM), cognitive behavioral therapy (CBT), multicomponent therapy (MT), mind–body therapy (MBT), enhanced care (EC), neurostimulation (NS), relaxation therapy (RT), conventional exercise (CE), and education (EDU)—did not show statistically significant improvements in anxiety symptoms compared with control. Furthermore, no significant differences were found in head-to-head comparisons among the 12 active interventions (**Supplementary Tables S4**, **S5**).

### Possible ranking of the effectiveness for treating anxiety

**Figure 5:**
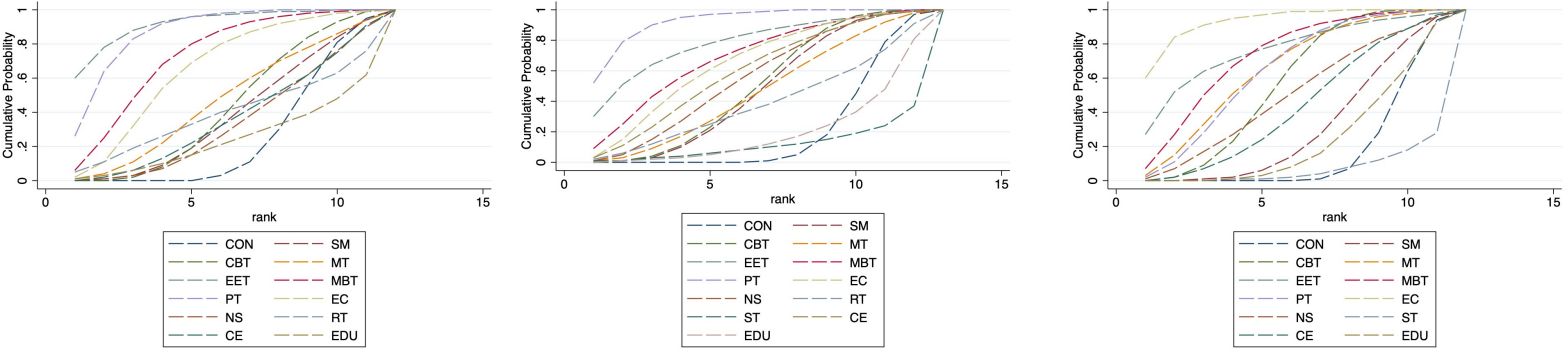
Cumulative probability plot for anxiety, depression and QoL.

Based on the SUCRA analysis, EET ranked as the most effective intervention for reducing anxiety, with a SUCRA score of 91.5% and a corresponding probability of 59.5% of being the best treatment. The full ranking of interventions from most to least effective was: EET (91.5%), PT (86.9%), MBT (72.7%), EC (65.2%), MT (46.6%), CBT (42.3%), RT (38.5%), SM (38.0%), CE (36.1%), NS (33.3%), CON (25.1%), and EDU (24.0%). (See **Figure 5**; **Supplementary Tables S6**, **S7**) This ranking indicates that EET and PT are the most promising interventions for anxiety reduction. Importantly, both EET (SMD: -1.80) and PT (SMD: -1.41) demonstrated statistically significant benefits compared to control. Although EET has a slightly higher SUCRA value, the direct head-to-head comparison between EET and PT did not show a statistically significant difference (**Supplementary Table S5**), suggesting their effects on anxiety may be comparable in clinical practice. The relatively wide confidence intervals for both EET and PT, however, indicate some uncertainty in the precise magnitude of their effects.

### Effects of non-pharmacological interventions on depression

A network meta-analysis of 41 RCTs involving 4,422 participants demonstrated that psychotherapy (PT) was significantly more effective than the control group in reducing depression symptoms, with an effect size of (SMD: −1.05, 95% CI, −1.71 to −0.38). The other 11 interventions—including SM, CBT, MT, EET, MBT, EC, NS, RT, supportive therapy (ST), CE, and EDU—did not yield statistically significant effects compared to control. In pairwise comparisons, PT was significantly superior to SM (SMD: −0.76, 95% CI, −1.50 to −0.02), EDU (SMD: −1.57, 95% CI, −2.99 to −0.16), and ST (SMD: −1.19, 95% CI, −2.16 to −0.21) in reducing depression symptoms (**Supplementary Tables S4**, **S5**).

### Possible ranking of the effectiveness for treating depression

SUCRA-based ranking identified PT as the most effective intervention for improving depressive symptoms, with a SUCRA score of 92.4% and a probability of 51.6% of being the most effective. The descending order of intervention effectiveness was: PT (92.4%), EET (78.2%), MBT (68.7%), EC (64.8%), CE (59.0%), NS (54.8%), CBT (49.3%), SM (47.0%), MT (46.1%), RT (38.5%), CON (20.3%), EDU (19.3%), and ST (11.6%). (See **Figure 5**; **Supplementary Tables S6**, **S7**) The SUCRA ranking aligns with the primary NMA findings: Psychotherapy (PT) is the only intervention demonstrating statistically significant reduction in depression symptoms compared to control (SMD: -1.05; 95% CI: -1.71 to -0.38). Its top ranking (SUCRA 92.4%)—substantially higher than the second-ranked EET (78.2%)—further supports PT’s clinical superiority. This is reinforced by direct head-to-head comparisons, where PT showed significant benefits over self-management (SM), education (EDU), and supportive therapy (ST).

While EET, MBT, and EC ranked highly by SUCRA, none achieved statistical significance versus control in the NMA(**Supplementary Table S5**). Thus, their high rankings should be interpreted with caution; they may reflect potential efficacy but lack robust statistical evidence in this analysis. Clinically, PT emerges as the only non-pharmacological intervention with confirmed efficacy for depression in epilepsy patients based on current evidence.

### Effects of non-pharmacological interventions on QoL

Compared to control, CBT, MBT, PT, and EC were all associated with statistically significant improvements in QoL among patients with epilepsy: CBT (SMD: 0.41, 95% CI, 0.07 to 0.76), MBT (SMD: 0.66, 95% CI, 0.03 to 1.29), PT (SMD: 0.52, 95% CI, 0.02 to 1.03), EC (SMD: 1.18, 95% CI, 0.42 to 1.94). Other interventions—such as SM, MT, EET, NS, RT, ST, CE, and EDU—did not significantly differ from the control group in improving QoL. In head-to-head comparisons: NS outperformed SM (SMD: 1.06; 95% CI, 0.21 to 1.90), EC outperformed CE (SMD: 0.92, 95% CI, 0.01 to 1.84), EET outperformed EDU (SMD: 1.13, 95% CI, 0.25 to 2.01). CBT, MBT, and EC outperformed ST with respective SMDs of: CBT (SMD: 0.76, 95% CI, 0.06 to 1.46), MBT (SMD: 1.01, 95% CI, 0.06 to 1.96), and EC (SMD: 1.53, 95% CI, 0.47 to 2.59)(**Supplementary Tables S4**, **S5**).

### Possible ranking of the effectiveness for improving QoL

SUCRA analysis indicated that EC was the most effective in improving QoL, with a SUCRA score of 93.1% and a probability of 59.5% of being the best intervention. The rankings were as follows: EC (93.1%), EET (76.2%), MBT (72.7%), MT (64.9%), PT (64.3%), CBT (56.6%), NS (49.9%), CE (42.8%), SM (30.9%), EDU (24.4%), CON (17.7%), and ST (6.6%). (See **Figure 5**, **Supplementary Tables S6**, **S7**) The SUCRA ranking suggests Enhanced Care (EC) as the potentially optimal intervention for QoL improvement. Critically, this is consistent with EC having the largest effect size versus control (SMD: 1.18; 95% CI: 0.42–1.94) and its significant superiority over Conventional Exercise (CE) and Supportive Therapy (ST) in direct comparisons. However, three other interventions—Mind-Body Therapy (MBT), Psychotherapy (PT), and Cognitive Behavioral Therapy (CBT)—also demonstrated statistically significant benefits for QoL (SMDs: 0.66, 0.52, 0.41 respectively), with SUCRA values >50%. While EC ranks highest, the overlapping confidence intervals of these four effective interventions (EC, MBT, PT, CBT) and lack of significant direct comparisons between them suggest their effects on QoL may be clinically comparable in practice.

Notably, Enhanced Education Therapy (EET) ranked second (SUCRA 76.2%) but did not achieve statistical significance versus control. Its high ranking should be interpreted cautiously as potential rather than confirmed efficacy.

### Subgroup analysis by age group

To explore potential age-related differences in intervention efficacy, we conducted age-stratified network meta-analyses for minors (≤18 years) and adults (>18 years), acknowledging that the applicability and impact of certain non-pharmacological interventions may vary across age groups.

In the pediatric subgroup, Enhanced Education Therapy (EET) was the most effective intervention for reducing anxiety symptoms (SUCRA = 97.6%; SMD = -1.85; 95% CI: -3.55 to -0.15), demonstrating clear superiority over control. Cognitive Behavioral Therapy (CBT) showed the greatest improvement in quality of life (QoL) (SUCRA = 99.5%; SMD = 1.50; 95% CI: 0.68 to 2.33), while both EET and Psychotherapy (PT) ranked highest for depression relief. PT, in particular, was significantly more effective than Enhanced Care (EC) (SMD = -0.28; 95% CI: -0.53 to -0.04).

In the adult subgroup, PT consistently emerged as the top-ranked intervention for both anxiety (SUCRA = 91.3%; SMD = 1.67; 95% CI: 0.39 to 2.96) and depression (SUCRA = 94.3%; SMD = 1.25; 95% CI: 0.42 to 2.09), significantly outperforming Self-Management in depressive symptom reduction. For QoL, Mind–Body Therapy (MBT) and EET were the highest-ranked interventions (SUCRA = 76.1% and 77.5%, respectively), with MBT showing statistically significant benefit over control (SMD: -0.71; 95% CI: -1.33 to -0.08).

These results underscore the need for age-specific intervention strategies. In minors, EET and CBT appear particularly beneficial for improving anxiety and QoL, while PT remains an effective option for depression. In adults, PT demonstrates consistent superiority across mental health outcomes, and MBT may offer unique advantages for enhancing QoL.

**Figure 6:**
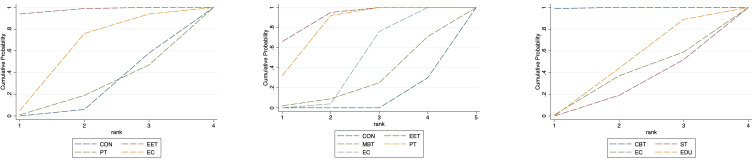
Cumulative probability plot for anxiety, depression and QoL in adolescents.

**Figure 7:**
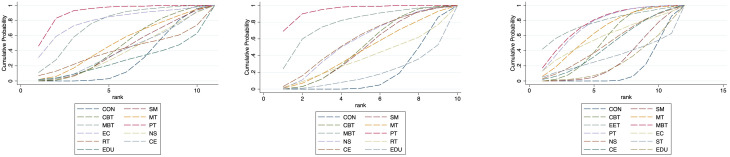
Cumulative probability plot for anxiety, depression and QoL in adults. CON, Control group; SM, Self-management; CBT, Cognitive-behavioral therapy; MT, Multi-component intervention; EET, Enhanced education therapy; MBT, Mind-body therapy; PT, Psychotherapy; EC, Enhanced care; NS, Neurostimulation; RT, Relaxation therapy; ST, Supportive therapy; CE, Conventional Exercise; EDU, Education.

Nevertheless, caution is warranted in interpreting the pediatric subgroup findings due to the relatively small sample size and wide confidence intervals. These findings require validation in future large-scale, age-targeted randomized trials. (See **Figure 6**, **7**, **Supplementary Tables S6**, **S7** for full details).

### Certainty of evidence

In order to evaluate the possibility of publication bias, we generated funnel plots corresponding to each outcome. Visual inspection indicated a potential bias was identified in the outcomes related to depression (see **Supplementary Tables S8**).

The quality of evidence was evaluated using the GRADE approach. The majority of direct evidence comparisons were assigned ratings of low to moderate confidence, largely driven by issues related to risk of bias, inconsistency, and imprecision (refer to **Supplementary Table S9** for details).

### Summary of evidence

This network meta-analysis synthesized evidence from 58 randomized controlled trials (6,541 participants across 20 countries) to compare multiple NPIs for people with epilepsy. The findings indicate that certain NPIs yielded greater improvements in mental health and QoL outcomes than others. In particular, enhanced educational therapy (EET) and psychotherapy (PT) emerged as the most effective interventions for reducing anxiety symptoms, while PT alone produced the largest reduction in depressive symptoms. For enhancing QoL, cognitive-behavioral therapy (CBT), mind–body therapy (MBT), PT, and enhanced care (EC) all showed significant benefits, with EC ranking highest according to SUCRA values.

A high SUCRA rank indicates a greater probability that an intervention is among the best options, but it does not automatically mean a dramatically large clinical advantage over other treatments (43). For example, although EC ranked first for QoL, the magnitude of QoL improvement with EC was only moderately higher than those of the next best interventions, and confidence intervals for some comparisons overlapped. This suggests that being top-ranked in SUCRA reflects relative efficacy but should be interpreted with caution regarding absolute clinical effect size. Likewise, while PT had the highest probability of treating depression effectively, differences between PT and the second-ranked intervention were not statistically significant in all comparisons. Overall, the evidence supports that tailored NPIs can meaningfully reduce anxiety and depression levels and improve daily functioning in epilepsy, but small between-intervention differences and overlapping outcomes mean that the best therapy may only be modestly more effective than the next best. Clinicians should thus use the rankings as a guide in conjunction with clinical judgment about effect sizes and patient context.

### Mechanisms and clinical significance

The consistent efficacy of psychotherapy (PT) across anxiety, depression, and QoL outcomes highlights its central role in improving the psychological well-being of epilepsy patients. PT interventions likely confer broad benefits by providing patients with emotional support, coping strategies, and skills to manage stressors related to epilepsy. Neurobiologically, psychological therapy has been associated with decreased hyperactivity in limbic and frontal regions of the brain (such as the anterior cingulate cortex, inferior frontal gyrus, and insula), which may alleviate negative emotional states (anxiety, depressed mood) and thereby enhance overall QoL. From a clinical perspective, this means that integrating formal psychotherapy into epilepsy care can simultaneously address multiple mental health dimensions, yielding improvements in mood and daily functioning. Our findings reinforce that CBT is another highly beneficial intervention, particularly for improving QoL. CBT operates by restructuring maladaptive beliefs and thought patterns; in epilepsy, it helps patients reframe catastrophic thoughts about seizures, reduce self-perceived stigma, and gain a sense of control. Physiologically, CBT has been shown to dampen stress-hormone levels (cortisol and ACTH), thereby attenuating hypothalamic–pituitary–adrenal (HPA) axis overactivation and relieving anxiety and depressive symptoms. Clinically, the implication is that CBT not only improves coping and mood through cognitive changes, but also produces measurable stress reduction in the body – a combination that can translate into better psychosocial adjustment and QoL. Similarly, mind–body therapies (MBT) (e.g. yoga, tai chi, mindfulness meditation) showed significant QoL benefits and some emotional symptom reduction. These practices emphasize slow, deep breathing and mindful physical activity, which help modulate autonomic nervous system balance. MBT techniques increase parasympathetic activity and raise central levels of gamma-aminobutyric acid (GABA), promoting relaxation and stress relief. Clinically, this mechanistic insight suggests that MBT can be prescribed as an adjunct to reduce stress-related symptoms; patients often report improved mood stability and fewer autonomic arousal symptoms (like panic or insomnia) with regular practice of yoga or similar exercises. Enhanced care (EC) and enhanced education therapy (EET), which were top-ranked for QoL and anxiety respectively, combine standard medical management with additional counseling, self-management training, or psychoeducation. These interventions likely work by reducing epilepsy-related stigma, correcting misconceptions, and empowering patients in active disease management, which in turn alleviates anxiety about seizures and improves social functioning and confidence. The high effectiveness of EET in anxiety suggests that when patients better understand their condition and acquire self-management skills, their uncertainty and fear diminish, leading to tangible anxiety reduction (44–55). Likewise, EC’s top ranking for QoL underscores the clinical significance of providing extra supportive care and attention: patients receiving EC often have more frequent contact with healthcare providers, personalized guidance, and encouragement, resulting in a greater sense of security and improved life quality. Taken together, the above mechanisms illustrate why the most effective interventions (PT, CBT, MBT, EET, EC) achieved superior outcomes – each targets different but complementary aspects of the patient’s mental health (cognitive reappraisal, emotional support, physiological calming, educational empowerment), thereby yielding meaningful improvements in well-being.

In contrast, our analysis also identified interventions with weak or no significant effects, notably supportive therapy (ST) and standard educational programs (EDU). ST and generic education tended to rank lower and did not produce reliable improvements in anxiety, depression, or QoL. There are plausible reasons for these limited outcomes. Supportive therapy primarily involves empathic listening and general emotional support without imparting specific cognitive-behavioral skills. While this approach can temporarily boost morale or provide comfort, it does not actively equip patients with coping strategies to manage their anxiety or depressive thoughts in the long run. Thus, patients receiving ST might feel understood during therapy sessions but still lack tools for sustainable symptom management or relapse prevention once sessions end. Clinically, this suggests that purely supportive counseling, although well-intentioned, may yield only minimal benefits for epilepsy-related anxiety/depression unless combined with skill-based techniques. Standard educational interventions (EDU), on the other hand, often consist of providing information about epilepsy (e.g. pamphlets, lectures or basic nurse counseling about seizures and medications). Our findings indicate that basic education alone had no significant impact on psychological outcomes, which may reflect several implementation challenges. Educational programs can fail to engage patients if they are not tailored to individual needs and cultural contexts. For example, patients with low health literacy or those facing cultural/language barriers may not absorb generic educational content, limiting its efficacy. Additionally, if educational sessions are too superficial or delivered in a one-size-fits-all manner, patients might not internalize the knowledge or change their health-related behaviors. In resource-limited settings, lack of time and trained personnel can further reduce the quality of educational interventions, resulting in low “implementation fidelity” (i.e., the intervention is not delivered as intended or at sufficient intensity) (56, 57).

These factors could explain why the trials of standard education in our analysis showed negligible improvements. From a clinical standpoint, the ineffectiveness of generic EDU means that simply handing out information is not enough – educational efforts must be enhanced (as seen with EET or EC) by making them interactive, personalized, and coupled with psychological support to truly affect patients’ mental well-being. Recognizing which interventions have limited yield is important for guiding healthcare resources: it would be prudent to prioritize therapies like PT, CBT, MBT, and EC that offer clear benefits, while refining or avoiding low-yield strategies like ST or unadapted education in routine practice. This targeted approach can ensure patients receive the most effective support for their mental health needs.

## Limitations and future directions

While this study provides a comprehensive comparative effectiveness overview, several limitations must be acknowledged when interpreting the results. First, there was substantial variability among the included trials in terms of participant characteristics, outcome measurement scales, intervention intensity, and follow-up durations. This clinical heterogeneity introduces uncertainty and may have impacted our treatment effect estimates. For instance, different studies used different anxiety and depression scales (e.g. HADS vs. BDI for depression), and follow-up periods ranged from a few weeks up to a year, which could lead to variability in observed outcomes. An intervention might show a strong short-term effect in a trial with brief follow-up, yet appear less effective in a trial assessing longer-term outcomes. Similarly, some trials focused on adults with severe epilepsy and comorbid psychiatric symptoms, whereas others included mixed populations or less severe cases – such differences in baseline severity and population could cause inconsistencies in how well an intervention works across studies. Although our network analysis passed global and local inconsistency tests (indicating the network was statistically coherent), we cannot exclude that this underlying heterogeneity contributed to wider confidence intervals and reduced precision in certain comparisons. In practical terms, this means the true magnitude of benefit for a given intervention may differ by patient subgroup or context, and our average estimates should be applied with that caution in mind.

Second, we identified potential publication bias for depression outcomes (e.g., a funnel plot asymmetry was observed in the depression domain). This bias suggests that smaller trials with negative or null results might be under-represented in the literature, leading to an overestimation of the efficacy of some interventions for depression. As a result, the comparative advantages observed for depression (especially for top-ranked therapies like PT) should be interpreted conservatively – the true effect in an unbiased scenario might be smaller. More broadly, several outcomes (particularly QoL and depression) were supported by evidence of generally low or moderate quality. Many trials had limited sample sizes and, due to the nature of behavioral interventions, most could not implement double-blinding. This leads to the third limitation: a risk of performance and detection bias. Participants and personnel were usually aware of the treatment being given (e.g. one cannot truly blind someone to whether they are receiving CBT or not), which can inflate reported benefits for subjective outcomes like mood and QoL. Lack of blinding could make intervention groups more prone to placebo effects or more active reporting of improvement, thereby biasing results in favor of the therapy. Additionally, some studies had other risk-of-bias issues (e.g. incomplete outcome data or unclear randomization procedures), which collectively lower confidence in certain findings. Fourth, the evidence base for a few interventions was relatively sparse. Therapies such as supportive counseling or standard education were each evaluated in only a handful of RCTs with small sample sizes, and often these were ancillary interventions rather than primary therapies. The limited number of studies for these interventions means their effect estimates carry greater uncertainty and their SUCRA rankings may be unreliable. In our case, interventions like ST and EDU might rank poorly in part because the evidence for them is not only indicating low effectiveness but is also of low quality, so we should be cautious in definitively labeling them as ineffective without further research.

To address heterogeneity, future studies should strive for more standardized outcome measures and consistent reporting – for example, using core outcome sets for depression, anxiety and QoL in epilepsy trials to ensure results are comparable across studies. Researchers could perform subgroup analyses or stratify randomization based on key characteristics (such as baseline depression severity, age group, or seizure frequency) to explore how different patient subsets respond to the same intervention. This approach would illuminate whether certain therapies work better for certain profiles (reflecting the principle of personalized medicine in psychosocial care). To mitigate publication bias, it is crucial to encourage preregistration of trials and the publication of negative findings. Investigators should report all outcomes transparently so that meta-analyses reflect the true balance of evidence. Where feasible, trial registries and collaboration among researchers can ensure that even non-significant or unfavorable results see the light of day. With regard to performance bias from lack of blinding, we acknowledge that blinding is challenging in behavioral trials; however, researchers can implement measures such as blinded outcome assessment (having an independent evaluator administer scales) or use active control groups (e.g., comparing CBT against a credible alternative program rather than a waitlist) to help reduce expectancy effects. Objective outcome metrics (like cortisol levels, if relevant, or seizure frequency as a co-outcome) could also supplement self-reported measures to provide more unbiased evidence of benefit. Finally, to improve the evidence base for under-studied interventions and to verify our findings, there is a need for large-scale, high-quality randomized controlled trials evaluating these NPIs. Ideally, future trials should enroll sufficient sample sizes to detect clinically meaningful differences, use rigorous methodologies (allocation concealment, intention-to-treat analysis, adequate follow-up), and report results according to CONSORT guidelines for transparency. Longer follow-up periods are especially important to determine the durability of NPI effects on psychological outcomes, since some benefits might wane over time if not reinforced.

Building on the current evidence, several future directions in research are suggested. One important avenue is the exploration of combined intervention strategies. Our results hinted that different NPIs have complementary mechanisms (cognitive, behavioral, physiological, educational), so combining them could yield additive or synergistic effects. For example, patients with comorbid anxiety and depression might benefit most from a combination like CBT plus psychotherapy (CBT+PT) – CBT could target maladaptive thoughts contributing to anxiety/depressive feelings, while psychotherapy sessions provide interpersonal support and emotion-focused coping, addressing both aspects of their mental health. To test such hypotheses, future trials could employ factorial study designs or sequential multiple assignment randomized trials (SMART). In a factorial RCT, for instance, one group might receive CBT, another receives PT, a third receives the combination CBT+PT, and a fourth receives standard care, allowing investigators to assess both individual and combined effects within the same trial. This design would clarify whether the combination yields superior outcomes compared to each component alone. Additionally, adaptive trial designs or stratified care models should be considered. A stratified or adaptive intervention model would involve tailoring the type or intensity of NPI to patient-specific factors – for example, those with high baseline anxiety might start with a more intensive therapy or a combo of interventions, whereas those with milder symptoms receive a single, targeted intervention. Over time, non-responders to one NPI could be “stepped up” to add another modality. Research using stratified approaches (potentially guided by biomarkers or validated risk profiles) could help identify optimal treatment matches for subgroups of patients, thereby maximizing efficacy. We have incorporated these ideas in the revised discussion to emphasize how future research can build upon our findings. In particular, we now explicitly suggest exploring synergistic effects of combined NPIs (e.g., CBT with PT for patients facing both anxiety and depression) through innovative trial designs, and we cite the potential of stratified adaptive models for personalized therapy selection. Furthermore, mechanistic studies remain a priority: employing neuroimaging or neurophysiological monitoring in future NPI trials could unravel the neural pathways or biomarkers that mediate improvement (for example, confirming whether therapies indeed normalize HPA-axis activity or functional connectivity in emotion-regulating circuits). Such insights would not only validate the mechanisms we propose but also guide refinement of interventions (e.g., if mindfulness is shown to activate certain calming brain networks, one might optimize that practice for greater effect). Finally, we underscore the importance of assessing cost-effectiveness and implementation feasibility of these NPIs in real-world clinical settings. As mental health care is integrated into epilepsy management, stakeholders will need evidence on how resource-intensive each intervention is, and whether benefits justify the costs. Future studies or pilot programs can evaluate delivery models (group vs individual therapy, in-person vs telehealth delivery of CBT, etc.) to find sustainable ways to make these interventions accessible to patients across diverse healthcare environments.

In summary, our revised discussion provides a clearer and more detailed interpretation of the evidence. We conclude that specific non-pharmacological interventions – especially psychotherapy, CBT, MBT, enhanced education, and enhanced care – demonstrate significant benefits for reducing anxiety and depression and for improving quality of life in people with epilepsy. Psychotherapy (PT) in particular showed a broad-spectrum efficacy across outcomes, underscoring its pivotal role in comprehensive epilepsy care. Meanwhile, enhanced educational and self-management programs (EET, EC) offer additional improvements, likely by empowering patients and addressing psychosocial factors like stigma. We have also acknowledged that some interventions (supportive therapy, standard education) appear less useful in their current forms, which is valuable information for clinicians when choosing how to allocate therapeutic time and resources. Our recommendations strongly advocate for incorporating individualized, evidence-based NPI programs into routine epilepsy management. Given that mental health outcomes are increasingly recognized as crucial to the overall well-being and social functioning of people with epilepsy, optimizing the use of NPIs will be essential. We envision that future clinical guidelines should integrate these findings by listing NPIs as core components of holistic epilepsy treatment (alongside pharmacological and surgical interventions). By continuing to refine these therapies through high-quality research and by targeting them to the right patients (or combinations of patients’ needs), the field can improve not only seizure control but also the QoL and psychiatric resilience of individuals living with epilepsy.

## Data Availability

The original contributions presented in the study are included in the article/**Supplementary Material**. Further inquiries can be directed to the corresponding author.
