# Supplementary material for: Comparative effectiveness of non-pharmacological interventions on anxiety, depression, and quality of life in patients with epilepsy: a systematic review and network meta-analysis
Source: Front Psychiatry. 2025 Jul 30;16:1624276. doi: 10.3389/fpsyt.2025.1624276 (PMC12345471; doi:10.3389/fpsyt.2025.1624276)
Supplement: Supplementary file 1 [file Table1.pdf]

SUPPLEMENTARY MATERIAL

Table of Contents

Supplementary Methods 1 – Database search strategy

Supplementary Methods 2 – Conversion formula for standard deviation (SD)

Supplementary Methods 3 – Inclusion in the list of studies

Supplementary Methods 4 – Definition of Interventions and Controls

Supplementary Table S1 – Table of Literature Characteristics

Supplementary Table S2 – Risk of bias table of included studies

Supplementary Table S3 – Inconsistency Test

Supplementary Table S3.1 – Loop Inconsistency Test

Supplementary Table S3.1.1 – Loop Inconsistency Test in adults

Supplementary Table S3.2 – Global Inconsistency Test

Supplementary Table S3.2.1 – Global Inconsistency Test in adults

Supplementary Table S3.3 – Local Inconsistency Test

Supplementary Table S3.3.1 – Local Inconsistency Test in adults

Supplementary Table S4 – Forest plot

Supplementary Table S4.1 – Forest plot for anxiety

Supplementary Table S4.1.1 – Forest plot for anxiety in adolescents

Supplementary Table S4.1.2 – Forest plot for anxiety in adults

Supplementary Table S4.2 – Forest plot for depression

Supplementary Table S4.2.1 – Forest plot for depression in adolescents

Supplementary Table S4.2.2 – Forest plot for depression in adults

Supplementary Table S4.3 – Forest plot for QoL

Supplementary Table S4.3.1 – Forest plot for QoL in adolescents

Supplementary Table S4.3.2 – Forest plot for QoL in adults

Supplementary Table S5 – League table

Supplementary Table S5.1 – Anxiety: league table

Supplementary Table S5.1.1 – Anxiety in adolescents: league table

Supplementary Table S5.1.2 – Anxiety in adults: league table

Supplementary Table S5.2 – Depression: league table

Supplementary Table S5.2.1 – Depression in adolescents: league table

Supplementary Table S5.2.2 – Depression in adults: league table

Supplementary Table S5.3 – QoL: league table

Supplementary Table S5.3.1 – QoL in adolescents: league table

Supplementary Table S5.3.2 – QoL in adults: league table

Supplementary Table S6 – SCURA score and ranking

Supplementary Table S6.1 – Sucra Score and Ranking in adults

Supplementary Table S6.2 – Ranking Probability for anxiety

Supplementary Table S6.2.1 – Ranking Probability for anxiety in adolescents

Supplementary Table S6.2.2 – Ranking Probability for anxiety in adults

Supplementary Table S6.3 – Ranking Probability for depression

Supplementary Table S6.3.1 – Ranking Probability for depression in adolescents

Supplementary Table S6.3.2 – Ranking Probability for depression in adults

Supplementary Table S6.4 – Ranking Probability for QoL

Supplementary Table S6.4.1 – Ranking Probability for QoL in adolescents

Supplementary Table S6.4.2 – Ranking Probability for QoL in adults

Supplementary Table S7 – Contribution graph

Supplementary Table S7.1 – Contribution graph for anxiety

Supplementary Table S7.1.1 – Contribution graph for anxiety in adolescents

Supplementary Table S7.1.2 – Contribution graph for anxiety in adults

Supplementary Table S7.2 – Contribution graph for depression

Supplementary Table S7.2.1 – Contribution graph for depression in adolescents

Supplementary Table S7.2.2 – Contribution graph for depression in adults

Supplementary Table S7.3 – Contribution graph for QoL

Supplementary Table S7.3.1 – Contribution graph for QoL in adolescents

Supplementary Table S7.3.2 – Contribution graph for QoL in adults

Supplementary Table S8 – Sensitivity analyses

Supplementary Table S8.1 – Sensitivity analyses for anxiety

Supplementary Table S8.1.1 – Sensitivity analyses for anxiety in adolescents

Supplementary Table S8.1.2 – Sensitivity analyses for anxiety in adults

Supplementary Table S8.2 – Sensitivity analyses for depression

Supplementary Table S8.2.1 – Sensitivity analyses for depression in adolescents

Supplementary Table S8.2.2 – Sensitivity analyses for depression in adults

Supplementary Table S8.3 – Sensitivity analyses for QoL

Supplementary Table S8.3.1 – Sensitivity analyses for QoL in adolescents

Supplementary Table S8.3.2 – Sensitivity analyses for QoL in adults

Supplementary Table S9 – GRADE assessment

Supplementary Table S9.1 – The Grading of Recommendations Assessment, Development and Evaluation (GRADE) assessment for anxiety

Supplementary Table S9.2 – The Grading of Recommendations Assessment, Development and Evaluation (GRADE) assessment for depression

Supplementary Table S9.3 – The Grading of Recommendations Assessment, Development and Evaluation (GRADE) assessment for QoL

Supplementary Table S10 – Major modifications for protocal in PROSPERO(CRD420251015149)

Methods 1 – Database search strategy

1.1 Database search strategy - Pubmed

**#1**	**"Epilepsy"[Mesh]**	132,564
#2	Epilepsy*[Title/Abstract] OR Epilepsies[Title/Abstract] OR Seizure*[Title/Abstract] OR Aura*[Title/Abstract] OR PWE[Title/Abstract] OR epileptic[Title/Abstract]	269,782
#3	**((((((((("Exercise"[Mesh]) OR "Sports"[Mesh]) OR "Therapeutics"[Mesh]) OR "therapy" [Subheading]) OR "Mindfulness"[Mesh]) OR "Relaxation"[Mesh]) OR "Yoga"[Mesh]) OR "Music"[Mesh]) OR "Qigong"[Mesh]) OR "Art"[Mesh]) OR "Dancing" [Mesh]**	11,038,172
#4	exercise*[Title/Abstract] OR sport*[Title/Abstract] OR Physical Activit* [Title/Abstract] OR training*[Title/Abstract] OR therap*[Title/Abstract] OR mindfulness[Title/Abstract] OR relaxation[Title/Abstract] OR yoga[Title/Abstract] OR music[Title/Abstract] OR education*[Title/Abstract] OR Ch'iKung[Title/Abstract] OR Qi Gong[Title/Abstract] OR art* [Title/Abstract] OR Danc*[Title/Abstract]	5,923,040
**#5**	**(("Depression"[Mesh]) OR "Anxiety"[Mesh]) OR "Quality of Life"[Mesh]**	530,775
#6	Depression*[Title/Abstract] OR Symptom*, Depressive[Title/Abstract] OR Depressive Symptom*[Title/Abstract] OR Anxiet*[Title/Abstract] OR Angst[Title/Abstract] OR Nervousness[Title/Abstract] OR Hypervigilance[Title/Abstract] OR Anxiousness[Title/Abstract] OR Life Quality[Title/Abstract] OR Quality Of Life[Title/Abstract] OR HRQOL[Title/Abstract] OR mental health[Title/Abstract] OR psychological health[Title/Abstract]	1,223,353
**#7**	**"Randomized Controlled Trial" [Publication Type]**	635,645
#8	randomized controlled trial* OR randomized clinical trial* OR randomized controlled clinical trial* OR RCT OR random control test* OR randomized controlled test* OR randomized comparison stud* OR randomized experiment* OR placebo* OR random allocation OR randomized	1,710,540
#9	("Epilepsy"[Mesh]) OR (Epilepsy*[Title/Abstract] OR Epilepsies[Title/Abstract] OR Seizure*[Title/Abstract] OR Aura* [Title/Abstract] OR PWE[Title/Abstract] OR epileptic[Title/Abstract])	290,535
#10	(((((((((("Exercise"[Mesh]) OR "Sports"[Mesh]) OR "Therapeutics"[Mesh]) OR "therapy" [Subheading]) OR "Mindfulness"[Mesh]) OR "Relaxation"[Mesh]) OR "Yoga"[Mesh]) OR "Music"[Mesh]) OR "Qigong"[Mesh]) OR "Art"[Mesh]) OR "Dancing"[Mesh]) OR (exercise*[Title/Abstract] OR sport*[Title/Abstract] OR Physical Activit*[Title/Abstract] OR training*[Title/Abstract] OR therap*[Title/Abstract] OR mindfulness[Title/Abstract] OR relaxation[Title/Abstract] OR yoga[Title/Abstract] OR music[Title/Abstract] OR education*[Title/Abstract] OR Ch'i Kung[Title/Abstract] OR Qi Gong[Title/Abstract] OR art* [Title/Abstract] OR	13,830,733

	Danc*[Title/Abstract])	
#11	((("Depression"[Mesh]) OR "Anxiety"[Mesh]) OR "Quality of Life"[Mesh]) OR (Depression*[Title/Abstract] OR Symptom*, Depressive[Title/Abstract] OR Depressive Symptom*[Title/Abstract] OR Anxiet*[Title/Abstract] OR Angst[Title/Abstract] OR Nervousness[Title/Abstract] OR Hypervigilance[Title/Abstract] OR Anxiousness[Title/Abstract] OR Life Quality[Title/Abstract] OR Quality Of Life[Title/Abstract] OR HRQOL[Title/Abstract] OR mental health[Title/Abstract] OR psychological health[Title/Abstract])	1,325,398
#12	("Randomized Controlled Trial" [Publication Type]) OR (randomized controlled trial* OR randomized clinical trial* OR randomized controlled clinical trial* OR RCT OR random control test* OR randomized controlled test* OR randomized comparison stud* OR randomized experiment* OR placebo* OR random allocation OR randomized)	1,711,245
#13	(((("Epilepsy"[Mesh]) OR (Epilepsy*[Title/Abstract] OR Epilepsies[Title/Abstract] OR Seizure*[Title/Abstract] OR Aura* [Title/Abstract] OR PWE[Title/Abstract] OR epileptic[Title/Abstract])) AND ((((((((((("Exercise"[Mesh]) OR "Sports"[Mesh]) OR "Therapeutics"[Mesh]) OR "therapy" [Subheading]) OR "Mindfulness"[Mesh]) OR "Relaxation"[Mesh]) OR "Yoga"[Mesh]) OR "Music"[Mesh]) OR "Qigong"[Mesh]) OR "Art"[Mesh]) OR "Dancing" [Mesh]) OR (exercise*[Title/Abstract] OR sport*[Title/Abstract] OR Physical Activit*[Title/Abstract] OR training*[Title/Abstract] OR therap*[Title/Abstract] OR mindfulness[Title/Abstract] OR relaxation[Title/Abstract] OR yoga[Title/Abstract] OR music[Title/Abstract] OR education*[Title/Abstract] OR Ch'i Kung[Title/Abstract] OR Qi Gong[Title/Abstract] OR art* [Title/Abstract] OR Danc*[Title/Abstract]))) AND (((("Depression" [Mesh]) OR "Anxiety"[Mesh]) OR "Quality of Life"[Mesh]) OR (Depression*[Title/Abstract] OR Symptom*, Depressive[Title/Abstract] OR Depressive Symptom*[Title/Abstract] OR Anxiet*[Title/Abstract] OR Angst[Title/Abstract] OR Nervousness[Title/Abstract] OR Hypervigilance[Title/Abstract] OR Anxiousness[Title/Abstract] OR Life Quality[Title/Abstract] OR Quality Of Life[Title/Abstract] OR HRQOL[Title/Abstract] OR mental health[Title/Abstract] OR psychological health[Title/Abstract]))) AND (("Randomized Controlled Trial" [Publication Type]) OR (randomized controlled trial* OR randomized clinical trial* OR randomized controlled clinical trial* OR RCT OR random control test* OR randomized controlled test* OR randomized comparison stud* OR randomized experiment* OR placebo* OR random allocation OR randomized))	1,769

Search date: March 19, 2025.

1.2 Database search strategy - Web of Science

#1	TS=(Epilepsy* OR Epilepsies OR Seizure* OR Aura* OR PWE OR epileptic)	441,743
#2	TS=(exercise* OR sports OR sport* OR Physical Activit* OR training* OR Therapeutics OR therapy OR therap* OR mindfulness OR mindfulness OR relaxation OR relaxation OR yoga OR yoga OR music)	16,102,708
#3	TS=(Depression* OR Symptom*, Depressive OR Depressive Symptom* OR Anxiet* OR Angst OR Nervousness OR Hypervigilance OR Anxiousness OR Quality of Life OR Life Quality OR Quality Of Life OR HRQOL OR mental health OR psychological health)	2,879,305
#4	TS=(randomized controlled trial* OR randomized clinical trial* OR randomized controlled clinical trial* OR RCT OR random control test* OR randomized controlled test* OR randomized comparison stud* OR randomized experiment* OR placebo* OR random allocation OR randomized)	1,661,649
#5	#4 AND #3 AND #2 AND #1	2,946

Search date: March 19, 2025.

1.3 Database search strategy - PsycInfo

#1	AB (Epilepsy* OR Epilepsies OR Seizure* OR Aura* OR PWE OR epileptic)	
#2	AB (exercise* OR sports OR sport* OR Physical Activit* OR training* OR Therapeutics OR therapy OR therap* OR mindfulness OR mindfulness OR relaxation OR relaxation OR yoga OR yoga OR music)	
#3	AB (Depression* OR Symptom*, Depressive OR Depressive Symptom* OR Anxiet* OR Angst OR Nervousness OR Hypervigilance OR Anxiousness OR Quality of Life OR Life Quality OR Quality Of Life OR HRQOL OR mental health OR psychological health)	
#4	AB (randomized controlled trial* OR randomized clinical trial* OR randomized controlled clinical trial* OR RCT OR random control test* OR randomized controlled test* OR randomized comparison stud* OR randomized experiment* OR placebo* OR random allocation OR randomized)	
#5	#4 AND #3 AND #2 AND #1	237

Search date: March 19, 2025.

1.3 Database search strategy - Cochrane

**#1**	**[Exercise] explode all trees**	39,411
**#2**	**[Sports] explode all trees**	22,421
**#3**	**[Therapeutics] explode all trees**	425,626
**#4**	**[Mindfulness] explode all trees**	2,411
**#5**	**[Relaxation] explode all trees**	2,173
**#6**	**[Yoga] explode all trees**	1,212
**#7**	**[Music] explode all trees**	1,216
**#8**	**[Education] explode all trees**	46,137
**#9**	**[Qigong] explode all trees**	169
#10	(exercise OR sport* OR Physical Activit* OR training* OR therap* OR mindfulness OR relaxation OR yoga OR music OR education* OR Ch'i Kung OR Qi Gong OR art* OR Danc*):ti,ab,kw	1,416,533
#11	#1 OR #2 OR #3 OR #4 OR #5 OR #6 OR #7 OR #8 OR #9 OR #10	1,474,618
#12	**[Epilepsy] explode all trees**	3,492
#13	(Epilepsy* OR Epilepsies OR Seizure* OR Aura* OR PWE OR epileptic):ti,ab,kw	18,300
#14	#12 OR #13	18,346
**#15**	**[Depression] explode all trees**	18,820
**#16**	**[Anxiety] explode all trees**	13,026
**#17**	**[Quality of Life] explode all tree**	44,016
#18	(Depression* OR Symptom*, Depressive OR Depressive Symptom* OR Anxiet* OR Angst OR Nervousness OR Hypervigilance OR Anxiousness OR Life Quality OR Quality Of Life OR HRQOL OR mental health OR psychological health):ti,ab,kw	371,605
#19	#15 OR #16 OR #17 OR #18	371,746
#20	#11 AND #14 AND #19	4,117

Search date: March 19, 2025.

1.4 Database search strategy - Embase

**#1**	**'epilepsy'/exp**	330,293
#2	'epilepsy*':ab,ti OR 'epilepsies':ab,ti OR 'seizure*':ab,ti OR 'aura*':ab,ti OR 'pwe':ab,ti OR 'epileptic':ab,ti	400,288
**#3**	**'exercise'/exp OR 'sport'/exp OR 'therapy'/exp OR 'mindfulness'/exp OR 'leisure'/exp OR 'yoga'/exp OR 'music'/exp OR 'education'/exp OR 'qigong'/exp OR 'art'/exp OR 'dancing'/exp**	13,899,542
#4	'exercise':ab,ti OR 'sport*':ab,ti OR 'physical activit*':ab,ti OR 'training*':ab,ti OR 'therap*':ab,ti OR 'mindfulness':ab,ti OR 'relaxation':ab,ti OR 'yoga':ab,ti OR 'music':ab,ti OR 'education*':ab,ti OR 'chi kung':ab,ti OR 'qi gong':ab,ti OR 'art*':ab,ti	11,237,788
**#5**	**'depression'/exp OR 'anxiety'/exp OR 'quality of life'/exp**	1,588,698
#6	'depression*':ab,ti OR 'symptom*, depressive':ab,ti OR 'depressive symptom*':ab,ti OR 'anxiet*':ab,ti OR 'angst':ab,ti OR 'nervousness':ab,ti OR 'hypervigilance':ab,ti OR 'anxiousness':ab,ti OR 'life quality':ab,ti OR 'quality of life':ab,ti OR 'hrqol':ab,ti OR 'mental health':ab,ti OR 'psychological health':ab,ti	1,686,312
**#7**	**'randomized controlled trial'/exp OR 'randomized controlled trial'**	1,181,508
#8	'randomized clinical trial*':ab,ti OR 'randomized controlled clinical trial*':ab,ti OR 'rct':ab,ti OR 'random control test*':ab,ti OR 'randomized controlled test*':ab,ti OR 'randomized comparison stud*':ab,ti OR 'randomized experiment*':ab,ti OR 'placebo*':ab,ti OR 'random allocation':ab,ti	548,870
#9	#1 OR #2	489,283
#10	#3 OR #4	19,168,538
#11	#5 OR #6	2,190,429
#12	**#7 OR #8**	1,425,150
#13	#9 AND #10 AND #11 AND #12	3,177

Search date: March 19, 2025.

Supplementary Methods 2 – Conversion formula for standard deviation (SD).

Standard Error to Standard Deviation:

SD=SE × $\sqrt{N}$ where SE is the standard error and N is the sample size.

95% Confidence Interval to Standard Deviation:

1 If the sample size of the test and control groups is greater than or equal to 100:

SD=$\sqrt{N}$×(Upper limit of credible intervals - Lower limit of credible intervals)/3.92

2 If the sample size of the test and control groups is less than or equal to 60:

SD=$\sqrt{N}$×(Upper limit of credible intervals - Lower limit of credible intervals)/ tinv (1-0.95,n-1)

3 For studies with sample sizes between 60 and 100 in each group, both of the above methods can be used.

Range converted SD:

SD= (Upper limit - lower limit)/4

Quartile converted SD:

SD= (Upper limit - lower limit)/1.35

Tinv: Represent probabilities, degrees of freedom in excel sheet

Supplementary Methods 3 – Inclusion in the list of studies.

1. Sajatovic, M., Colon-Zimmermann, K., Kahriman, M., Fuentes-Casiano, E., Liu, H.,Tatsuoka, C., Cassidy, K. A., Lhatoo, S., Einstadter, D., & Chen, P. (2018). A 6-month prospective randomized controlled trial of remotely delivered group format epilepsy self-management versus waitlist control for high-risk people with epilepsy. Epilepsia, 59(9), 1684–1695. https://doi.org/10.1111/epi.14527

2. Bennett, S. D., Cross, J. H., Chowdhury, K., Ford, T., Heyman, I., Coughtrey, A. E., Dalrymple, E., Byford, S., Chorpita, B., Fonagy, P., Moss-Morris, R., Reilly, C., Smith, J. A., Stephenson, T., Varadkar, S., Blackstone, J., Quartly, H., Hughes, T., Lewins, A., . . . Shafran, R. (2024). Clinical effectiveness of the psychological therapy Mental Health Intervention for Children with Epilepsy in addition to usual care compared with assessment-enhanced usual care alone: a multicentre, randomised controlled clinical trial in the UK. The Lancet, 403(10433), 1254–1266. https://doi.org/10.1016/s0140-6736(23)02791-5

3. Gandy, M., Sharpe, L., Perry, K. N., Thayer, Z., Miller, L., Boserio, J., & Mohamed, A. (2014). Cognitive Behaviour Therapy to Improve Mood in People with Epilepsy: A Randomised Controlled Trial. Cognitive Behaviour Therapy, 43(2), 153–166. https://doi.org/10.1080/16506073.2014.892530

4. Goldstein, L. H., Robinson, E. J., Mellers, J. D. C., Stone, J., Carson, A., Reuber, M., Medford, N., McCrone, P., Murray, J., Richardson, M. P., Pilecka, I., Eastwood, C., Moore, M., Mosweu, I., Perdue, I., Landau, S., Chalder, T., & CODES study group (2020). Cognitive behavioural therapy for adults with dissociative seizures (CODES): a pragmatic, multicentre, randomised controlled trial. The lancet. Psychiatry, 7(6), 491–505. https://doi.org/10.1016/S2215-0366(20)30128-0

5. Goldstein, L. H., Chalder, T., Chigwedere, C., Khondoker, M. R., Moriarty, J., Toone, B. K., & Mellers, J. D. (2010). Cognitive-behavioral therapy for psychogenic nonepileptic seizures: a pilot RCT. Neurology, 74(24), 1986–1994. https://doi.org/10.1212/WNL.0b013e3181e39658

6. Beretta, S., Beghi, E., Messina, P., Gerardi, F., Pescini, F., La Licata, A., Specchio, L., Ferrara, M., Canevini, M. P., Turner, K., La Briola, F., Franceschetti, S., Binelli, S., Giglioli, I., Galimberti, C. A.,

Fattore, C., Zaccara, G., Tramacere, L., Sasanelli, F., Pirovano, M., … Ferrarese, C. (2014). Comprehensive educational plan for patients with epilepsy and comorbidity (EDU-COM): a pragmatic randomised trial. Journal of neurology, neurosurgery, and psychiatry, 85(8), 889–894. https://doi.org/10.1136/jnnp-2013-306553

7. Wang, J., Chen, W., & Lai, Y. (2023). Comprehensive nursing program for children with epilepsy: A randomized controlled trial. Nigerian journal of clinical practice, 26(10), 1498–1504. https://doi.org/10.4103/njcp.njcp_93_23

8. Wijnen, B. F. M., Leenen, L. A. M., de Kinderen, R. J. A., van Heugten, C. M., Majoie, M. H. J. M., & Evers, S. M. A. A. (2017). An economic evaluation of a multicomponent self-management intervention for adults with epilepsy (ZMILE study). Epilepsia, 58(8), 1398–1408. https://doi.org/10.1111/epi.13806

9. Hu, M., Zhang, C., Xiao, X., Guo, J., & Sun, H. (2020). Effect of intensive self-management education on seizure frequency and quality of life in epilepsy patients with prodromes or precipitating factors. Seizure, 78, 38–42. https://doi.org/10.1016/j.seizure.2020.03.003

10. Zhuang, C., Wu, H., Lin, B., & An, X. (2021). The effect of Omaha System-based continuous nursing care on the psychological status, self-esteem, and quality of life in epileptic children. American journal of translational research, 13(4), 3435–3442.

11. Yadegary, M. A., Maemodan, F. G., Nayeri, N. D., & Ghanjekhanlo, A. (2015). The effect of self-management training on health-related quality of life in patients with epilepsy. Epilepsy & behavior : E&B, 50, 108–112. https://doi.org/10.1016/j.yebeh.2015.04.051

12. Ridsdale, L., Wojewodka, G., Robinson, E. J., Noble, A. J., Morgan, M., Taylor, S. J. C., McCrone, P., Richardson, M. P., Baker, G., Landau, S., & Goldstein, L. H. (2018). The effectiveness of a group self-management education course for adults with poorly controlled epilepsy, SMILE (UK): A randomized controlled trial. Epilepsia, 59(5), 1048–1061. https://doi.org/10.1111/epi.14073

13. Leenen, L. A. M., Wijnen, B. F. M., Kessels, A. G. H., Chan, H., de Kinderen, R. J. A., Evers, S. M. A. A., van Heugten, C. M., & Majoie, M. H. J. M. (2018). Effectiveness of a multicomponent self-management intervention for adults with epilepsy (ZMILE study): A randomized controlled trial. Epilepsy & behavior : E&B, 80, 259–265. https://doi.org/10.1016/j.yebeh.2018.01.019

14. Schröder, J., Brückner, K., Fischer, A., Lindenau, M., Köther, U., Vettorazzi, E., & Moritz, S. (2014). Efficacy of a psychological online intervention for depression in people with epilepsy: a randomized controlled trial. Epilepsia, 55(12), 2069–2076. https://doi.org/10.1111/epi.12833

15. Mohamadpour, S., Tajikzadeh, F., & Aflakseir, A. (2017). The Efficacy of Mindfulness-Based Cognitive therapy on Self-Efficacy and anxiety among epileptic patients. Archives of Neuroscience, In press(In press). https://doi.org/10.5812/archneurosci.39060

16. Tsai, S. Y., Lee, W. T., Lee, C. C., Jeng, S. F., & Weng, W. C. (2024). Five-year follow-up of a clinic-based sleep intervention for paediatric epilepsy: A randomized clinical trial. Journal of sleep research, 33(3), e14059. https://doi.org/10.1111/jsr.14059

17. Eshiet, U. I., Okonta, J. M., & Ukwe, C. V. (2021). Impact of a pharmacist-led education and counseling interventions on quality of life in epilepsy: A randomized controlled trial. Epilepsy research, 174, 106648. https://doi.org/10.1016/j.eplepsyres.2021.106648

18. Lin, C. Y., Potenza, M. N., Broström, A., Blycker, G. R., & Pakpour, A. H. (2019). Mindfulness-based cognitive therapy for sexuality (MBCT-S) improves sexual functioning and intimacy among older women with epilepsy: A multicenter randomized controlled trial. Seizure, 73, 64–74. https://doi.org/10.1016/j.seizure.2019.10.010

19. Lai, S. T., Lim, K. S., Tang, V., & Low, W. Y. (2021). Mindfulness-based intervention to promote psychological wellbeing in people with epilepsy: A randomized controlled trial. Epilepsy & behavior : E&B, 118, 107916. https://doi.org/10.1016/j.yebeh.2021.107916

20. Caller, T. A., Ferguson, R. J., Roth, R. M., Secore, K. L., Alexandre, F. P., Zhao, W., Tosteson, T. D., Henegan, P. L., Birney, K., & Jobst, B. C. (2016). A cognitive behavioral intervention (HOBSCOTCH) improves quality of life and attention in epilepsy. Epilepsy & behavior : E&B, 57(Pt A), 111–117. https://doi.org/10.1016/j.yebeh.2016.01.024

21. Pramuka, M., Hendrickson, R., Zinski, A., & Van Cott, A. C. (2007). A psychosocial self-management program for epilepsy: a randomized pilot study in adults. Epilepsy & behavior : E&B, 11(4), 533–545. https://doi.org/10.1016/j.yebeh.2007.06.013

22. Helde, G., Bovim, G., Bråthen, G., & Brodtkorb, E. (2005). A structured, nurse-led intervention program improves quality of life in patients with epilepsy: a randomized, controlled trial. Epilepsy & behavior : E&B, 7(3), 451–457. https://doi.org/10.1016/j.yebeh.2005.06.008

23. Sajatovic, M., Tatsuoka, C., Welter, E., Perzynski, A. T., Colon-Zimmermann, K., Van Doren, J. R., Bukach, A., Lawless, M. E., Ryan, E. R., Sturniolo, K., & Lhatoo, S. (2016). Targeted Self-Management of Epilepsy and Mental Illness for individuals with epilepsy and psychiatric comorbidity. Epilepsy & behavior : E&B, 64(Pt A), 152–159. https://doi.org/10.1016/j.yebeh.2016.08.012

24. Dorris, L., Broome, H., Wilson, M., Grant, C., Young, D., Baker, G., Balloo, S., Bruce, S., Campbell, J., Concannon, B., Conway, N., Cook, L., Davis, C., Downey, B., Evans, J., Flower, D., Garlo VS ky, J., Kearney, S., Lewis, S., Stephens, V., … Wright, I. (2017). A randomized controlled trial of a manual-based psychosocial group intervention for young people with epilepsy [PIE]. Epilepsy & behavior : E&B, 72, 89–98. https://doi.org/10.1016/j.yebeh.2017.04.007

25. Azmoodeh, S., Soleimani, E., & Issazadegan, A. (2021). The Effects of Transcranial Direct Current Stimulation on Depression, Anxiety, and Stress in Patients with Epilepsy: A Randomized Clinical Trial. Iranian journal of medical sciences, 46(4), 272–280. https://doi.org/10.30476/ijms.2020.83233.1215

26. Gil-López, F., Boget, T., Manzanares, I., Donaire, A., Conde-Blanco, E., Baillés, E., Pintor, L., Setoaín, X., Bargalló, N., Navarro, J., Casanova, J., Valls, J., Roldán, P., Rumià, J., Casanovas, G.,

Domenech, G., Torres, F., & Carreño, M. (2020). External trigeminal nerve stimulation for drug resistant epilepsy: A randomized controlled trial. Brain stimulation, 13(5), 1245–1253. https://doi.org/10.1016/j.brs.2020.06.005

27. Mota, S. M., Amaral de Castro, L., Riedel, P. G., Torres, C. M., Bragatti, J. A., Brondani, R., Secchi, T. L., Sanches, P. R. S., Caumo, W., & Bianchin, M. M. (2021). Home-Based Transcranial Direct Current Stimulation for the Treatment of Symptoms of Depression and Anxiety in Temporal Lobe Epilepsy: A Randomized, Double-Blind, Sham-Controlled Clinical Trial. Frontiers in integrative neuroscience, 15, 753995. https://doi.org/10.3389/fnint.2021.753995

28. Lundgren, T., Dahl, J., Yardi, N., & Melin, L. (2008). Acceptance and Commitment Therapy and yoga for drug-refractory epilepsy: a randomized controlled trial. Epilepsy & behavior : E&B, 13(1), 102–108. https://doi.org/10.1016/j.yebeh.2008.02.009

29. Feng, H. X., Wang, M. X., Zhao, H. M., Hou, X. X., Xu, B., Gui, Q., Wu, G. H., Dong, X. F., Xu, Q. R., Shen, M. Q., Shi, Q. R., Cheng, Q. Z., & Xue, S. R. (2022). Effect of cognitive behavioral intervention on anxiety, depression, and quality of life in patients with epilepsy. American journal of translational research, 14(7), 5077–5087.

30. Meyer, B., Weiss, M., Holtkamp, M., Arnold, S., Brückner, K., Schröder, J., Scheibe, F., & Nestoriuc, Y. (2019). Effects of an epilepsy-specific Internet intervention (Emyna) on depression: Results of the ENCODE randomized controlled trial. Epilepsia, 60(4), 656–668. https://doi.org/10.1111/epi.14673

31. Häfele, C. A., Rombaldi, A. J., Feter, N., Häfele, V., Gervini, B. L., Domingues, M. R., & da Silva, M. C. (2021). Effects of an exercise program on health of people with epilepsy: A randomized clinical trial. Epilepsy & behavior : E&B, 117, 107904. https://doi.org/10.1016/j.yebeh.2021.107904

32. Etemadifar, S., Heidari, M., Jivad, N., & Masoudi, R. (2018). Effects of family-centered empowerment intervention on stress, anxiety, and depression among family caregivers of patients with epilepsy. Epilepsy & behavior : E&B, 88, 106–112. https://doi.org/10.1016/j.yebeh.2018.08.030

33. May, T. W., & Pfäfflin, M. (2002). The efficacy of an educational treatment program for patients with epilepsy (MOSES): results of a controlled, randomized study. Modular Service Package Epilepsy. Epilepsia, 43(5), 539–549. https://doi.org/10.1046/j.1528-1157.2002.23801.x

34. Meyer, B., Betz, L. T., Brückner, K., & Holtkamp, M. (2024). Enhancing quality of life in epilepsy with a digital intervention (emyna): Results of the ELAINE randomized controlled trial. Epilepsia open, 9(5), 1758–1771. https://doi.org/10.1002/epi4.13014

35. Chen, H. F., Tsai, Y. F., Fan, J. Y., Chen, M. C., Hsi, M. S., & Hua, M. S. (2021). Evaluation of a self-management intervention for adults with epilepsy in Taiwan: A longitudinal randomized controlled trial. Epilepsy & behavior : E&B, 117, 107845. https://doi.org/10.1016/j.yebeh.2021.107845

36. Lundgren, T., Dahl, J., Melin, L., & Kies, B. (2006). Evaluation of acceptance and commitment therapy for drug refractory epilepsy: a randomized controlled trial in South Africa--a pilot study. Epilepsia, 47(12), 2173–2179. https://doi.org/10.1111/j.1528-1167.2006.00892.x

37. Paardekooper, D., Thayer, Z., Miller, L., Nikpour, A., & Gascoigne, M. B. (2020). Group-based cognitive behavioral therapy program for improving poor sleep quality and quality of life in people with epilepsy: A pilot study. Epilepsy & behavior : E&B, 104(Pt A), 106884. https://doi.org/10.1016/j.yebeh.2019.106884

38. Kavuran, E., Özalp, C., & Ay, E. (2025). The impact of a walking program on self-management, anxiety, stress, depression, quality of life, and seizure frequency in patients with epilepsy: A mixed methods approach using the COM-B behaviour change model. Epilepsy & behavior : E&B, 162, 110149. https://doi.org/10.1016/j.yebeh.2024.110149

39. Fontaine, S., Gautier, L., Diependaele, A. S., Hamieh, M., Morello, R., Guillouët, S., & Bertran, F. (2023). Impact of educational actions on the quality of life of patients with epilepsy: A randomised controlled trial. Epilepsy research, 192, 107128. https://doi.org/10.1016/j.eplepsyres.2023.107128

40. Kumar, M., Ramanujam, B., Barki, S., Dwivedi, R., Vibha, D., Singh, R. K., & Tripathi, M. (2022). Impact of exercise as a complementary management strategy in people with epilepsy: A randomized controlled trial. Epilepsy & behavior : E&B, 129, 108616. https://doi.org/10.1016/j.yebeh.2022.108616

41. Sharma, S., Nehra, A., Pandey, S., Tripathi, M., Srivastava, A., Padma, M. V., Garg, A., Pandey, R. M., Chandra, S., & Tripathi, M. (2024). Neuropsychological Rehabilitation for Epilepsy in India: Looking Beyond the Basics. Epilepsy & behavior : E&B, 153, 109703. https://doi.org/10.1016/j.yebeh.2024.109703

42. Fraser, R. T., Johnson, E. K., Lashley, S., Barber, J., Chaytor, N., Miller, J. W., Ciechanowski, P., Temkin, N., & Caylor, L. (2015). PACES in epilepsy: Results of a self-management randomized controlled trial. Epilepsia, 56(8), 1264–1274. https://doi.org/10.1111/epi.13052

43. Martinović, Z., Simonović, P., & Djokić, R. (2006). Preventing depression in adolescents with epilepsy. Epilepsy & behavior : E&B, 9(4), 619–624. https://doi.org/10.1016/j.yebeh.2006.08.017

44. Johnson, E. K., Fraser, R. T., Lashley, S., Barber, J., Brandling-Bennett, E. M., Vossler, D. G., Miller, J. W., Caylor, L., & Warheit-Niemi, T. (2020). Program of Active Consumer Engagement in Self-Management in Epilepsy: Replication and extension of a self-management randomized controlled trial. Epilepsia, 61(6), 1129–1141. https://doi.org/10.1111/epi.16530

45. McAuley, J. W., Long, L., Heise, J., Kirby, T., Buckworth, J., Pitt, C., Lehman, K. J., Moore, J. L., & Reeves, A. L. (2001). A Prospective Evaluation of the Effects of a 12-Week Outpatient Exercise Program on Clinical and Behavioral Outcomes in Patients with Epilepsy. Epilepsy & behavior : E&B, 2(6), 592–600. https://doi.org/10.1006/ebeh.2001.0271

46. Olley, B. O., Osinowo, H. O., & Brieger, W. R. (2001). Psycho-educational therapy among Nigerian adult patients with epilepsy: a controlled outcome study. Patient education and counseling, 42(1), 25–33. https://doi.org/10.1016/s0738-3991(00)00087-2

47. Åkerlund, S., Varkey, E., Klecki, J., Zelano, J., & Ben-Menachem, E. (2021). Randomized controlled trial of moderate cardiovascular exercise for patients with drug-resistant epilepsy. Epilepsy & behavior : E&B, 124, 108335. Advance online publication. https://doi.org/10.1016/j.yebeh.2021.108335

48. McLaughlin, D. P., & McFarland, K. (2011). A randomized trial of a group based cognitive behavior therapy program for older adults with epilepsy: the impact on seizure frequency, depression and psychosocial well-being. Journal of behavioral medicine, 34(3), 201–207. https://doi.org/10.1007/s10865-010-9299-z

49. Moncrief, G. G., Aita, S. L., Tyson, B. T., Abecassis, M., Roth, R. M., Caller, T. A., Schmidt, S. S., & Jobst, B. C. (2021). Self-rated executive dysfunction in adults with epilepsy and effects of a cognitive-behavioral intervention (HOBSCOTCH). Epilepsy & behavior : E&B, 121(Pt A), 108042. https://doi.org/10.1016/j.yebeh.2021.108042

50. Modi, A. C., Guilfoyle, S. M., Glauser, T. A., & Mara, C. A. (2021). Supporting treatment adherence regimens in children with epilepsy: A randomized clinical trial. Epilepsia, 62(7), 1643–1655. https://doi.org/10.1111/epi.16921

51. Spruill, T. M., Friedman, D., Diaz, L., Butler, M. J., Goldfeld, K. S., O'Kula, S., Montesdeoca, J., Payano, L., Shallcross, A. J., Kaur, K., Tau, M., Vazquez, B., Jongeling, A., Ogedegbe, G., & Devinsky, O. (2021). Telephone-based depression self-management in Hispanic adults with epilepsy: a pilot randomized controlled trial. Translational behavioral medicine, 11(7), 1451–1460. https://doi.org/10.1093/tbm/ibab045

52. Ahorsu, D. K., Lin, C. Y., Imani, V., Carlbring, P., Nygårdh, A., Broström, A., Hamilton, K., & Pakpour, A. H. (2020). Testing an app-based intervention to improve insomnia in patients with epilepsy: A randomized controlled trial. Epilepsy & behavior : E&B, 112, 107371. https://doi.org/10.1016/j.yebeh.2020.107371

53. Huang, X., Kang, Y., Wang, M., Liu, Q., Wang, F., & Zeng, M. (2023). WeChat-based remote

follow-up management reduces the burden of home care and anxiety on parents of children with refractory epilepsy: A randomized controlled study. Medicine, 102(25), e34070. https://doi.org/10.1097/MD.0000000000034070

54. Meador, K. J., Kapur, R., Loring, D. W., Kanner, A. M., Morrell, M. J., & RNS® System Pivotal Trial Investigators (2015). Quality of life and mood in patients with medically intractable epilepsy treated with targeted responsive neurostimulation. Epilepsy & behavior : E&B, 45, 242–247. https://doi.org/10.1016/j.yebeh.2015.01.012

55. Yang, H., Shi, W., Fan, J., Wang, X., Song, Y., Lian, Y., Shan, W., & Wang, Q. (2023). Transcutaneous Auricular Vagus Nerve Stimulation (ta-VNS) for Treatment of Drug-Resistant Epilepsy: A Randomized, Double-Blind Clinical Trial. Neurotherapeutics : the journal of the American Society for Experimental NeuroTherapeutics, 20(3), 870–880. https://doi.org/10.1007/s13311-023-01353-9

56. Lim, K. S., Fong, S. L., Yu, X., Lim, Y. H., Wong, K. Y., Lai, S. T., Ng, C. G., Tan, C. T., & Tan, S. B. (2024). The effect of 20-minute mindful breathing exercise on psychological well-being in epilepsy: A pilot randomized controlled trial. Epilepsy & behavior : E&B, 155, 109778. https://doi.org/10.1016/j.yebeh.2024.109778

57. Ibañez-Micó, S., Gil-Aparicio, R., & Gómez-Conesa, A. (2024). Effect of a physical exercise program supported by wearable technology in children with drug-resistant epilepsy. A randomized controlled trial. Seizure, 121, 56–63. https://doi.org/10.1016/j.seizure.2024.07.019

58. Kaur, K., Sharma, G., Dwivedi, R., Nehra, A., Parajuli, N., Upadhyay, A. D., Deepak, K. K., Jat, M. S., Ramanujam, B., Sagar, R., Mohanty, S., & Tripathi, M. (2023). Effectiveness of Yoga Intervention in Reducing Felt Stigma in Adults With Epilepsy: A Randomized Controlled Trial. Neurology, 101(23), e2388–e2400. https://doi.org/10.1212/WNL.0000000000207944

Supplementary Methods 4 – Interventions Included in the Network Meta-analysis

Group	Abbreviation	Definition / Description
**Contral group**	**CON**	Reference comparator, which may include usual care, waitlist, attention control, or minimal intervention conditions.
**Self-management**	**SM**	Self-management therapy is based on the social cognitive theory, which focuses on the cognitive, emotional and behavioral aspects of behavior change. Patients take specific actions such as making wise nursing decisions, carrying out activities to manage diseases, and using necessary skills to maintain good psychosocial functioning, control the condition, reduce flare-ups, and maintain normal functioning.
**Cognitive-behavioral therapy**	**CBT**	A structured, time-limited psychotherapy focusing on modifying dysfunctional thoughts, behaviors, and emotional responses. Widely used for anxiety and depression management.
**Multi-component intervention**	**MT**	The therapeutic regimen that involves the concurrent application of multiple distinct intervention measures or strategies.
**Enhanced education therapy**	**EET**	Patient education programs that go beyond routine information delivery, including skills training, personalized coaching, or interactive educational modules aimed at self-management and behavior change.
**Mind-body therapy**	**MBT**	A group of interventions that integrate physical practices (e.g., yoga, breathing exercises, meditation) with mental focus to promote emotional regulation and stress reduction.
**Psychotherapy**	**PT**	Broad category of psychological interventions including various talk therapies beyond CBT, such as interpersonal therapy, psychodynamic therapy, or supportive counseling.
**Enhanced care**	**EC**	Multicomponent care models (e.g., collaborative care, stepped care, case management) designed to improve access, coordination, and personalization of psychosocial or mental health services.
**Neurostimulation**	**NS**	A therapeutic approach that regulates the function of the nervous system through physical means (such as Vagus Nerve Stimulation and Transcranial Magnetic Stimulation), commonly used for neurological disorders.
**Relaxation therapy**	**RT**	A set of therapeutic methods aimed at helping people reduce tension and anxiety. These methods induce the body's "relaxation response," characterized by slower breathing, reduced blood pressure, a slower heart rate, and improved stress. For example, muscle relaxation training.

**Supportive therapy**	**ST**	A type of therapy aimed at providing help, encouragement, or care for patients, typically used to complement other treatments to enhance the patient's treatment experience and outcomes.
**Conventional Exercise**	**CE**	A series of traditional, planned, organized, and repetitive physical activities aimed at improving physical fitness and health. Exercise methods typically include aerobic exercise, resistance training, and integrated training.
**Education**	**EDU**	A type of educational intervention carried out by healthcare professionals who have undergone specialized training. It aims to improve clinical outcomes and support patients in self-managing chronic diseases with the help of caregivers and family members, thereby generating additional therapeutic effects.

Supplementary Table S1 – Table of Literature Characteristics

	Author,Year	Country (Region)	% of Men	Sample (n)	Age mean± sd or range	Type of Intervention	Intervening duration	Evaluation of outcome	Type of outcome
1	Sajatovic,2018	USA	35.0<br>28.8	53<br>58	41.5±12.3<br>41.0±11.4	SM<br>CON	60-90min,1time per week ,8-10weeks<br>/	PHQ-9<br>QOLIE-10	②③
2	Bennett,2024	UK	51.2<br>48.2	132<br>132	10.5±3.6<br>10.3±4.0	PT<br>EC	1time per week,20times<br>/	PHQ-9 GAD-7	①②
3	Gandy,2014	AUS	50.0<br>24.0	20<br>25	41.0±12.0<br>38.0±13.0	CBT<br>CON	60min,1time per week ,9weeks<br>/	HADS<br>QOLIE-31	①②③
4	Goldstein,2020	UK	24.7<br>30.8	186<br>182	37.3±14.2<br>37.7±14.5	CBT<br>CON	1-2times per week ,4month<br>/	GAD-7 PHQ-9<br>EQ-5D-5L	①②③
5	Goldstein,2010	UK	27.3<br>22.6	33<br>31	37.4±12.6<br>35.9±15.1	CBT<br>CON	1-2times per week ,4month<br>/	HADS	①②
6	Beretta,2014	IT	57.1<br>51.8	91<br>83	NR	EDU<br>CON	1 time per month,2month<br>/	QOLIE-31	③
7	Wang,2023	CHN	62.9<br>54.3	35<br>35	12.0±2.5<br>13.0±2.2	EC<br>CON	3 month<br>3 month	SAS,SDS<br>WHOQL	①②③
8	Wijnen,2017	NLD	53.9<br>44.0	52<br>50	40.0±13.1<br>43.5±15.4	MT<br>CON	1time per week,9weeks<br>/	QOLIE-31-P	③
9	Hu,2020	CHN	40.0<br>42.6	45<br>47	28.7±7.3<br>31.9±9.8	EET<br>EDU	1 time per month,12month<br>1 time per month,12month	QOLIE-31	③
10	Zhuang,2021	CHN	72.3<br>69.4	65<br>62	8.0±1.6<br>8.2±1.7	EC<br>CON	15-45min,1 time per week,3 month<br>15-45min,1 time per week,3 month	SAS,SDS<br>CE-QOL	①②

11	Yadegary,2015	IRN	53.3	30	26.7±6.7	SM	120min,1time per week ,1 month	QOLIE-31-P	③
			50.0	30	26.6±6.7	CON	/		
12	Ridsdale,2018	UK	43.9	205	42.5±14.3	SM	total 16 hours,Four and a half days	HADS	①②③
			47.7	199	40.8±14.0	CON	/	QOLIE-31-P	
13	Leenen,2018	NLD	53.9	52	40.0±13.1	SM	120min,1time per week ,8 weeks	HADS	①②③
			44.0	50	43.5±15.4	CON	/	QOLIE-31-P	
14	Schröder,2014	DEU	34.2	38	35.0±10.0	PT	arrange time freely,9weeks	BDI-I	②③
			15.0	40	40.0±11.9	CON	/	QOLIE-31	
15	Mohamadpour,2017	IRN	80.0	15	32.8±7.7	MBT	60-120min,1time per week ,8weeks	SAS	①
			73.3	15	34.8±6.0	CON	/		
16	Tsai,2024	TWN	54.2	24	4.0±1.4	EDU	3 times	CES-D	②
			55.6	18	4.0±1.2	CON	/		
17	Eshiet,2021	NGA	60.8	79	NR	EDU	2 times per month,6 month	QOLIE-10P	③
			57.7	78		CON	/		
18	Lin,2019	IRN	NR	220	71.3±18.9	CBT	90min,1time per week ,8 weeks	HADS	①②③
				220	70.6±24.9	MT	90min,1time per week ,8 weeks	QOLIE-31	
				220	72.4±20.1	CON	/		
19	Lai,2021	MYS	28.6	14	34.1±14.3	MBT	150min,1time per week ,6 weeks	BAI BDI-II	①②③
			50.0	14	36.5±13.1	CON	/	QOLIE-31	
20	Caller,2016	USA	34.5	29	49.3±9.2	MT	1time per week ,8 weeks	PHQ-9	②③
			35.0	20	41.4±11.2	CON	/	QOLIE-31	
21	Pramuka,2007	USA	41.9	31	48.9±14.3	PT	120min,1time per week ,6 weeks	QOLIE-89	③
			29.2	24	48.1±14.3	CON	/		
22	Helde,2005	NOR	43.1	57	35.3±16.7	EC	a day	QOLIE-89	③
			39.3	54	39.5±16.7	CON	/		

23	Sajatovic,2016	USA	40.9	22	52.0±7.6	SM	60-90min,1time per week ,12weeks	PHQ-9	②③
			42.9	22	45.1±14.2	CON	/	QOLIE-10	
24	Dorris,2017	UK	35.0	40	14.4±1.5	PT	120min,1time per week ,6 weeks	PedsQL 4.0	③
			44.2	43	14.3±1.4	CON	/		
25	Azmoodeh,2021	IRN	53.3	15	38.1±9.1	NS	20min,10times	DASS-21	①②
			53.3	15	34.7±9.3	CON	/		
26	Lopez,2020	ESP	0.4	20	44.2±11.0	NS	480min,everyday,a year	HADS BDI	①②③
			0.5	20	37.1±12.7	CON	/	QOLIE-31	
27	Mota,2021	BRA	7.7	13	53.4±14.5	NS	20min,1 time a day,4weeks	HAM-A BDI	①②③
			23.1	13	55.8±7.7	CON	/	QOLIE-31	
28	Lundgrena,2008	SWE	70.0	10	21.9	CBT	90min,6 times,5 week	WHOQOL-BREF	③
			62.5	8	25.8	MBT	90min,6 times,5 week		
29	Feng,2022	CHN	45.7	46	30.6±5.6	CBT	60min,1time per week ,8weeks	SAS HDMA	①②③
			40.8	49	31.2±6.3	CON	/	QOLIE-31	
30	Meyer,2019	DEU	35.0	100	40.5±12.9	CBT	6 month	GAD-7 PHQ-9	①②③
			38.0	100	40.1±13.4	CON	/	QOLIE-10	
31	Hafele,2021	BRA	36.4	11	37.1±13.7	CE	60min,2 times per week ,12weeks	STAI NDDI-E	①②③
			40.0	10	39.7±10.8	CON	/	QOLIE-31	
32	Etemadifar,2018	IRN	31.7	41	35.8±11.6	MT	5 times per week ,4weeks	DASS	①②
			46.2	39	35.6±10.1	CON	/		
33	May,2002	DEU	42.5	113	37.5±13.7	EDU	2 days	D-S	②
			43.4	129	38.4±13.5	CON	/		
34	Meyer,2024	DEU	31.5	216	37.3±13.6	CBT	arrange time freely,9weeks	DASS-21	③
			27.9	222	37.7±14.3	CON	/	QOLIE-31	
35	Chen,2021	CHN	54.7	75	39.1±10.4	SM	30min,3 times per month,6 month	HADS	①②③

			57.5	80	40.7±10.5	CON	/	QOLIE-31	
36	Lundgren,2006	SWE	50.0	14	38.85	CBT	90-180min,4 times,5 weeks	WHOQOL-BREF	③
			46.2	13	42.5	ST	90-180min,4 times,5 weeks		
37	Paardekooper,2020	AUS	18.2	11	39.2±10.9	CBT	120min,1 time per week,4 weeks	QOLIE-31	③
			22.2	9	47.4±12.2	CON	/		
38	Kavuran,2025	TUR	36.9	38	38.0±12.2	CE	30-45min,2 times per week,12weeks	DASS-21	①②③
			55.0	40	42.9±10.6	CON	/	SF-12	
39	Fontaine,2023	FRA	30.8	39	38.2±14.9	EDU	2 hours,3 times,6 month	QOLIE-31	③
			36.6	41	42.6±17.1	CON	/		
40	Kumar,2022	IND	67.2	58	26.2±7.9	CE	150min per week ,12 weeks	QOLIE-31	③
			72.9	59	27.1±8.0	CON	/		
41	Sharma,2024	IND	66.7	15	28.1±8.5	PT	20-40min,6 times per week,6 weeks	HAM-A HAM-D	①②③
			69.2	13	26.6±8.4	CON	/	QOLIE-31	
42	Fraser,2015	USA	43.9	41	44.9±12.5	SM	75min,1time per week,8 weeks	GAD-7 PHQ-9	①②③
			45.2	42	45.4±12.6	CON	/	QOLIE-31	
43	Martinovic,2006	SRB	40.0	15	17.2±2.5	CBT	1-4times per month,12 times,6 month	BDI	②③
			40.0	15	17.6±2.2	ST	1-4times per month,12 times,6 month	QOLIE-31	
44	Johnson,2020	USA	36.7	49	42.9±14.3	SM	60-75min,1time per week,8 weeks	PHQ-9	②③
			44.2	52	42.8±13.7	CON	/	QOLIE-31	
45	McAuley,2001	USA	42.9	14	40	CE	60min,1time per week,12 weeks	POMS	②③
			33.3	9	37.2	CON	/	QOLIE-89	
46	Olley,2001	NGA	58.6	15	32.6±10.2	PT	2 days	BDI CCEI	①②
				15		CON	/		
47	Akerlund,2021	SWE	41.7	12	48	CE	30min,5 times per week,6 month	HADS	①②
			33.3	10	41	RT	30min,5 times per week,6 month		

48	Mclaughlin,2010	AUS	44.4	18	67.6±7.3	CBT	120 min,1 time per week,6 weeks	CIDI	②
			52.6	19	67.4±7.5	RT	120 min,1 time per week,6 weeks		
49	Moncrief,2021	USA	32.3	31	49.2±9.1	CBT	45-60min,total 8 times,2 month	PHQ-9	②
			35.0	20	41.5±11.2	CON	/		
50	Modi,2021	USA	44.4	27	7.1±2.8	ST	15-45min,1 time per 2 weeks,4 month	QOLCE	③
			51.7	29	8.2±3.3	EDU	15-45min,1 time per 2 weeks,4 month		
51	Spruill,2021	USA	27.8	36	47.0±11.4	SM	60min,1 time per week,8 weeks	PHQ-9	②
			30.6	36	39.6±10.0	CON	/		
52	Ahorsu,2020	HK	39.4	160	38.4±13.5	CBT	1 time per week,6 weeks	HADS	①②③
			43.8	160	38.0±9.9	EDU	1 time per week,6 weeks	QOLIE-31	
53	Huang,2023	CHN	63.0	81	4.9±1.2	EET	3 time per month,6 month	SAS,SDS	①②
			59.3	81	4.6±1.1	CON	/		
54	Meador,2015	USA	52.0	93	34.9±11.6	NS	4 month	BDI-II	②③
				87		CON	/	QOLIE-89	
55	Yang,2023	CHN	56.5	100	33.3±11.3	NS	120min,1 time per week,20 weeks	HAMA HAMD	①②
			52.8	50	34.0±10.8	CON	/	QOLIE-31	
56	Lim,2024	MYS	37.5	10	34.1±9.9	MBT	20 min,2 times per week,2 weeks	GAD-7 NDDI-E	①②③
			62.5	10	32.1±14.0	CON	/	QOLIE-31	
57	Mico,2024	ESP	35.7	14	10.9	CE	60min,3 times per week,6 month	QOLCE-55	③
			46.7	15	8.73	EDU	55min,2 times per week,6 month		
58	Kaur,2023	IND	67.5	80	29.9±8.3	MBT	30 min,5 times per week,3 month	QoLIE-10	③
			62.5	80	29.4±8.4	PT	30 min,5 times per week,3 month		

CON: Control group **SM**: Self-management **CBT**: Cognitive-behavioral therapy **MT**: Multi-component intervention **EET**: Enhanced education therapy
MBT: Mind-body therapy **PT**: Psychotherapy **EC**: Enhanced care **NS**: Neurostimulation **RT**: Relaxation therapy **ST**: Supportive therapy **CE**: Conventional Exercise
EDU: Education

Supplementary Table S2 – Risk of bias table of included studies

Study	Randomization process	Deviations from intended interventio	Missing outcome data	Measurement of the outcome	Selection of the reported result	Overall
Sajatovic,2018	Low risk	Some concerns	Low risk	Low risk	Some concerns	Some concerns
Bennett,2024	Low risk	Low risk	Low risk	Low risk	Low risk	Low risk
Gandy,2014	Low risk	High risk	Some concerns	Low risk	Some concerns	High risk
Goldstein,2020	Low risk	Some concerns	Low risk	Low risk	Low risk	Some concerns
Goldstein,2010	Low risk	Some concerns	Some concerns	Low risk	Low risk	Some concerns
Beretta,2014	Low risk	Some concerns	Some concerns	Low risk	Low risk	Some concerns
Wang,2023	Low risk	Some concerns	Some concerns	Low risk	Low risk	Some concerns
Wijnen,2017	Low risk	Some concerns	Some concerns	Low risk	Low risk	Some concerns
Hu,2020	Low risk	Some concerns	Some concerns	Low risk	Low risk	Some concerns
Zhuang,2021	Some concerns	Some concerns	Low risk	Low risk	Low risk	Some concerns
Yadegary,2015	Some concerns	Low risk	Low risk	Low risk	Low risk	Some concerns
Ridsdale,2018	Low risk	Low risk	Low risk	Low risk	Low risk	Low risk
Leenen,2018	Low risk	Low risk	Low risk	Low risk	Low risk	Low risk
Schröder,2014	Low risk	Some concerns	Some concerns	Low risk	Low risk	Some concerns
Mohamadpour,2017	Low risk	Low risk	Low risk	Low risk	Low risk	Low risk
Tsai,2024	Low risk	Some concerns	High risk	Low risk	Low risk	High risk
Eshiet,2021	Low risk	Some concerns	Some concerns	Low risk	Low risk	Some concerns
Lin,2019	Low risk	Some concerns	Low risk	Low risk	Low risk	Some concerns
Lai,2021	Low risk	Some concerns	Low risk	Low risk	Low risk	Some concerns
Caller,2016	Low risk	Low risk	Some concerns	Low risk	Low risk	Some concerns
Pramuka,2007	Low risk	Low risk	Some concerns	Low risk	Low risk	Some concerns
Helde,2005	Low risk	Low risk	Low risk	Low risk	Low risk	Low risk
Sajatovic,2016	Low risk	Low risk	Some concerns	Low risk	Low risk	Some concerns
Dorris,2017	Low risk	Some concerns	Some concerns	Low risk	Low risk	Some concerns
Azmoodeh,2021	Some concerns	Some concerns	Low risk	Low risk	Low risk	Some concerns
Lopez,2020	Some concerns	Some concerns	Low risk	Low risk	Low risk	Some concerns
Mota,2021	Low risk	Some concerns	Some concerns	Low risk	Low risk	Some concerns
Lundgrena,2008	Low risk	Low risk	Low risk	Low risk	Low risk	Low risk
Feng,2022	Some concerns	Some concerns	Low risk	Low risk	Low risk	Some concerns
Meyer,2019	Low risk	Low risk	Some concerns	Low risk	Some concerns	Some concerns
Hafele,2021	Low risk	Low risk	Low risk	Low risk	Low risk	Low risk
Etemadifar,2018	Some concerns	Some concerns	Some concerns	Low risk	Low risk	Some concerns
May,2002	Low risk	Low risk	Some concerns	Low risk	Some concerns	Some concerns
Meyer,2024	Low risk	Some concerns	Some concerns	Low risk	Some concerns	Some concerns
Chen,2021	Some concerns	High risk	High risk	Low risk	Some concerns	High risk
Lundgren,2006	Low risk	Low risk	Low risk	Low risk	Low risk	Low risk
Paardekooper,2020	Some concerns	Some concerns	Low risk	Low risk	Low risk	Some concerns
Kavuran,2025	Some concerns	Some concerns	Low risk	Low risk	Low risk	Some concerns
Fontaine,2023	Low risk	Low risk	Low risk	Low risk	Low risk	Low risk
Kumar,2022	Low risk	Low risk	Low risk	Low risk	Low risk	Low risk
Sharma,2024	Some concerns	Some concerns	Low risk	Low risk	Low risk	Low risk
Fraser,2015	Some concerns	Some concerns	Low risk	Low risk	Low risk	Low risk
Martinovic,2006	Some concerns	Low risk	Low risk	Low risk	Low risk	Low risk
Johnson,2020	Low risk	Low risk	Low risk	Low risk	Low risk	Low risk
McAuley,2001	Some concerns	Some concerns	Some concerns	Low risk	Low risk	Some concerns
Olley,2001	Some concerns	Some concerns	High risk	High risk	Low risk	High risk
Akerlund,2021	Low risk	Low risk	Low risk	Low risk	Low risk	Low risk
Mclaughlin,2010	Low risk	Low risk	Low risk	Low risk	Low risk	Low risk
Moncrief,2021	Low risk	Low risk	Low risk	Low risk	Low risk	Low risk
Modi,2021	Low risk	Low risk	Low risk	Low risk	Low risk	Low risk
Spruill,2021	Low risk	Low risk	Low risk	Low risk	Low risk	Low risk
Ahorsu,2020	Low risk	Low risk	Low risk	Low risk	Low risk	Low risk
Huang,2023	Some concerns	Some concerns	Some concerns	Low risk	Low risk	Some concerns
Meador,2015	Some concerns	Low risk	Some concerns	Low risk	Low risk	Some concerns
Yang,2023	Some concerns	Low risk	Some concerns	Low risk	Low risk	Some concerns
Lim,2024	Low risk	Low risk	Low risk	Low risk	Low risk	Low risk
Mico,2024	Some concerns	High risk	High risk	High risk	Low risk	High risk
Kaur,2023	Low risk	Some concerns	Low risk	Low risk	Low risk	Low risk

+ Low risk
? Some concerns
? High risk

Supplementary Table S3 – Inconsistency Test

Supplementary Table S3.1 – Loop Inconsistency Test

Anxiety<br>(28 studies, *N* = 3554)		Depression<br>(40 studies, *N* = 4422)		Quality of Life<br>(43 studies, *N* = 4823)	
Type	P value	Type	P value	Type	P value
CON-PT-EC	0.656	CON-PT-EC	0.699	CON-MBT-PT	0.298
CON-CBT-MT	0.358	CON-CBT-RT-CE	0.330	CBT-RT-EDU	0.323
		CON-CBT-EDU	0.235	CON-CE-EDU	0.570
		CON-CBT-MT	0.946	CON-CBT-MBT	0.724
				CON-CBT-EDU	0.796
				CON-CBT-MT	0.658

Supplementary Table S3.1.1 – Loop Inconsistency Test in adults

Anxiety		Depression		Quality of Life	
Type	P value	Type	P value	Type	P value
CON-CBT-MT	0.358	CON-CBT-RT-CE	0.330	CON-MBT-PT	0.486
		CON-CBT-EDU	0.235	CON-CBT-MBT	0.724
		CON-CBT-MT	0.946	CON-CBT-MT	0.658
				CON-CBT-EDU	0.854

Notes: CON：Control group **SM**：Self-management **CBT**：Cognitive-behavioral therapy **MT**：Multi-component intervention **EET**：Enhanced education therapy **MBT**：Mind-body therapy **PT**：Psychotherapy **EC**：Enhanced care **NS**：Neurostimulation **RT**：Relaxation therapy **ST**：Supportive therapy **CE**：Conventional Exercise **EDU**：Education

Supplementary Table S3.2 – Global Inconsistency Test

Anxiety (28 studies, *N* = 3554)		Depression (40 studies, *N* = 4422)		Quality of Life (43 studies, *N* = 4823)	
Type		Type		Type	
Chi^2	0.65	Chi^2	1.42	Chi^2	2.18
Pro > Chi2	0.8849	Pro > Chi2	0.9222	Pro > Chi2	0.9494

Supplementary Table S3.2.1 – Global Inconsistency Test in adults

Anxiety		Depression		Quality of Life	
Type		Type		Type	
Chi^2	0.20	Chi^2	0.79	Chi^2	1.25
Pro > Chi2	0.9059	Pro > Chi2	0.9403	Pro > Chi2	0.9401

Supplementary Table S3.3 – Local Inconsistency Test

Anxiety (28 studies, *N* = 3554)		Depression (40 studies, *N* = 4422)		Quality of Life (43 studies, *N* = 4823)	
Side	P > ∣z∣	Side	P > ∣z∣	Side	P > ∣z∣
A B	.	A B	.	A B	.
A C*	0.707	A C	0.864	A C	0.939
A D*	0.851	A D*	0.878	A D*	0.840
A E	.	A E	.	A F	0.272
A F	.	A F	.	A G	0.166
A G	0.481	A G	0.476	A H	.
A H	0.481	A H	0.476	A I	.
A I	.	A I	.	A K	0.569
A K*	0.999	A L	0.416	A L	0.909
C D	0.945	A M	0.404	C D	0.952
C L*	0.998	C D	0.947	C F	0.846
G H	0.481	C J	0.416	C J	0.568
J K*	0.999	C K*	0.998	C L	0.899
		C M	0.404	E L*	0.993
		G H	0.476	F G	0.166
		J L	0.416	J L	0.568
				K L	0.569

*： All the evidence about there contrasts comes from the trails which directly compare them.

Notes (Anxiety): A： Control group **B：** Self-management **C：** Cognitive-behavioral therapy **D：** Multi-component intervention **E：**Enhanced education therapy **F：**Mind-body therapy **G：**Psychotherapy **H：** Enhanced care **I：** Neurostimulation **J：** Relaxation therapy **K：** Conventional Exercise **L：** Education

Notes(Depression): A： Control group **B：** Self-management **C：** Cognitive-behavioral therapy **D：** Multi-component intervention **E：**Enhanced education therapy **F：**Mind-body therapy **G：**Psychotherapy **H：** Enhanced care **I：** Neurostimulation **J：** Relaxation therapy **K：** Supportive therapy **L：** Conventional Exercise **M：** Education

Notes(QoL): A： Control group **B：** Self-management **C：** Cognitive-behavioral therapy **D：** Multi-component intervention **E：**Enhanced education therapy **F：**Mind-body therapy **G：**Psychotherapy **H：** Enhanced care **I：** Neurostimulation **J：** Supportive therapy **K：** Conventional Exercise **L：** Education

Supplementary Table S3.3.1 – Local Inconsistency Test in adults

Anxiety		Depression		Quality of Life	
Side	P ＞ ｜z｜	Side	P ＞ ｜z｜	Side	P ＞ ｜z｜
A B	.	A B	.	A B	.
A C *	0.722	A C	0.904	A C	0.888
A D *	0.860	A D*	0.893	A D*	0.802
A E	.	A E	.	A F	0.332
A F *	0.987	A F	.	A G	0.247
A H	.	A G	.	A H	.
A J *	0.999	A I	0.461	A I	.
C D	0.948	A J	0.467	A K	.
C K *	0.998	C D	0.954	A L	0.956
F G *	0.987	C H	0.461	C D	0.921
I J *	0.999	C J	0.467	C F	0.934
		H I	0.461	C J*	0.997
				C L	0.956
				E L*	0.993
				F G	0.247

*： All the evidence about there contrasts comes from the trails which directly compare them.

Notes (Anxiety): A： Control group **B：** Self-management **C：** Cognitive-behavioral therapy

D： Multi-component intervention **E：** Mind-body therapy **F：** Psychotherapy **G：** Enhanced care

H： Neurostimulation **I：** Relaxation therapy **J：** Conventional Exercise **K：** Education

Notes(Depression): A： Control group **B：** Self-management **C：** Cognitive-behavioral therapy

D： Multi-component intervention **E：** Mind-body therapy **F：** Psychotherapy **G：** Neurostimulation

H： Relaxation therapy **I：** Conventional Exercise **J：** Education

Notes(QoL): A： Control group **B：** Self-management **C：** Cognitive-behavioral therapy

D： Multi-component intervention **E：** Enhanced education therapy **F：** Mind-body therapy

G： Psychotherapy **H：** Enhanced care **I：** Neurostimulation **J：** Supportive therapy

K： Conventional Exercise **L：** Education

Supplementary Table S4 – Forest plot

Supplementary Table S4.1 – Forest plot for anxiety

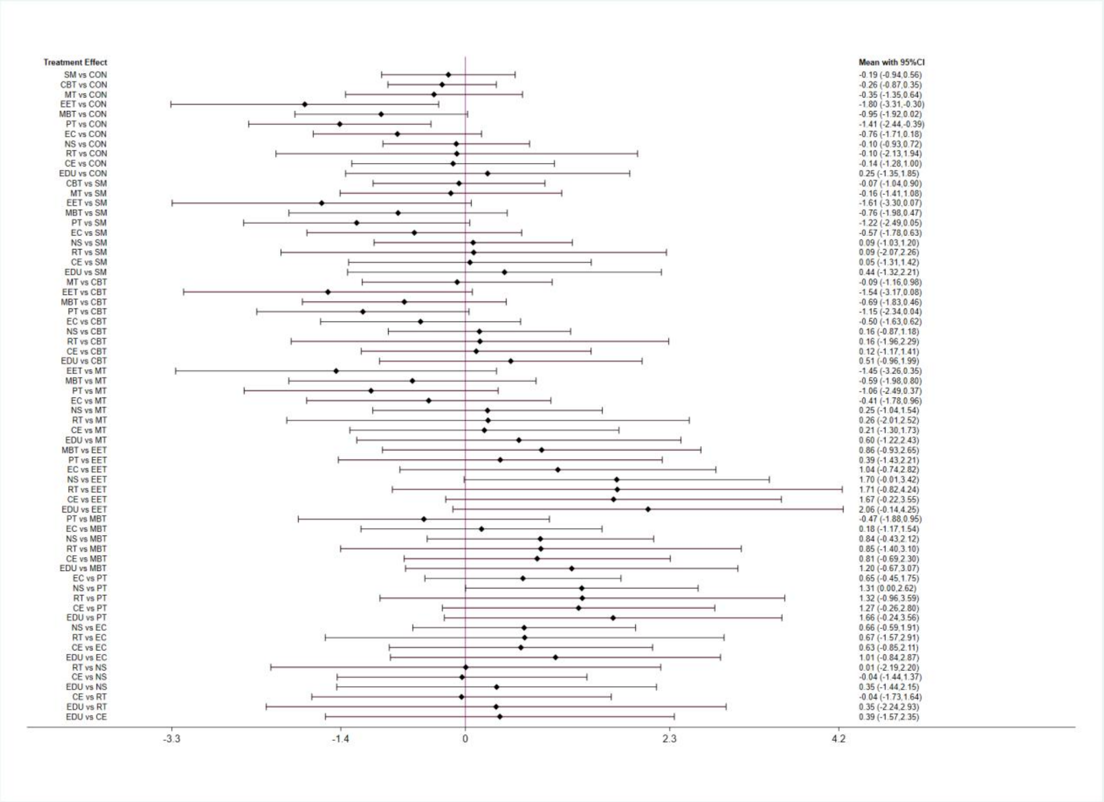

Notes: CON：Control group **SM**：Self-management **CBT**：Cognitive-behavioral therapy **MT**：Multi-component intervention **EET**：Enhanced education therapy **MBT**：Mind-body therapy **PT**：Psychotherapy **EC**：Enhanced care **NS**：Neurostimulation **RT**：Relaxation therapy **CE**：Conventional Exercise **EDU**：Education

Supplementary Table S4.1.1 – Forest plot for anxiety in adolescents

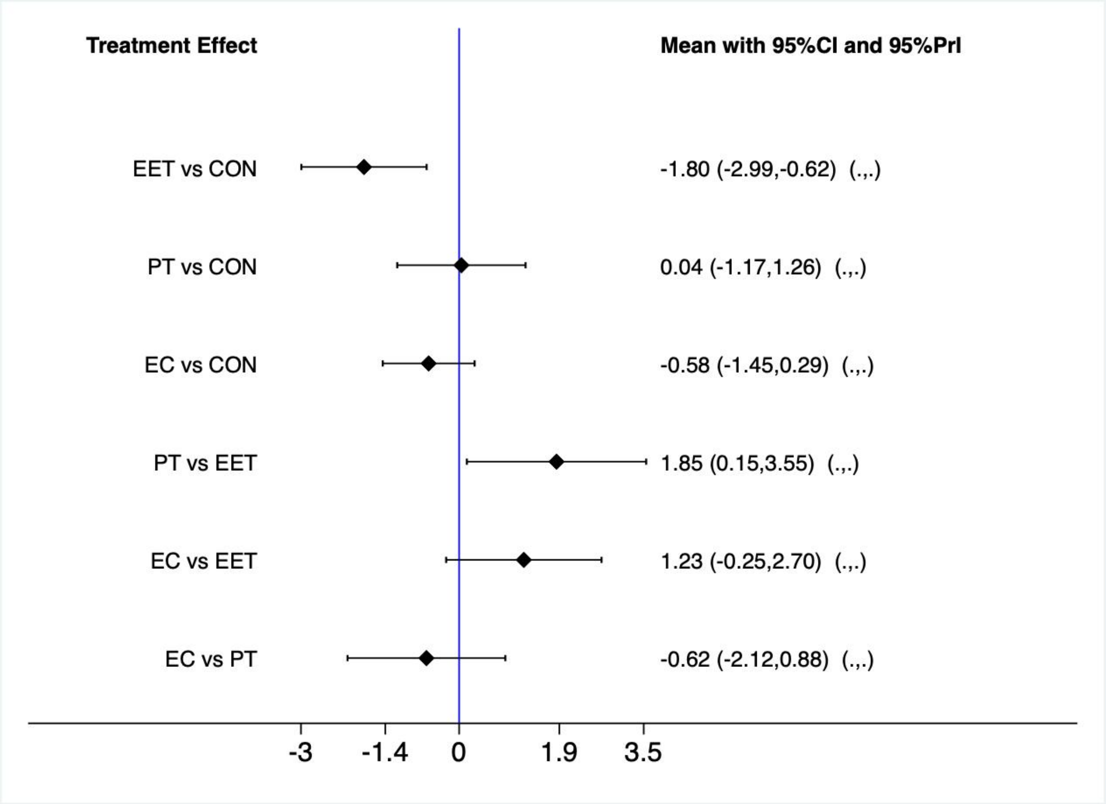

Notes: CON： Control group **EET：** Enhanced education therapy **PT：** Psychotherapy **EC：** Enhanced care

Supplementary Table S4.1.2 – Forest plot for anxiety in adults

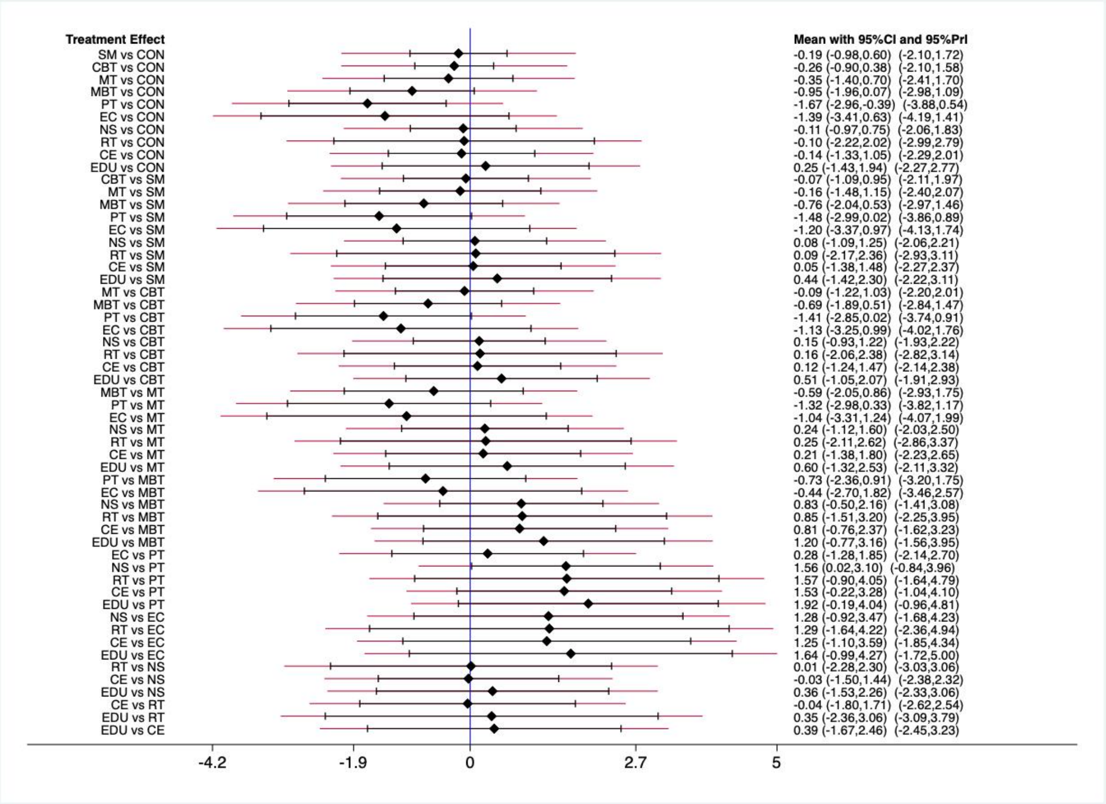

Notes: CON：Control group **SM：**Self-management **CBT：**Cognitive-behavioral therapy **MT：**Multi-component intervention **MBT：** Mind-body therapy **PT：** Psychotherapy **EC：** Enhanced care **NS：** Neurostimulation **RT：** Relaxation therapy **CE：**Conventional Exercise **EDU：**Education

Supplementary Table S4.2 – Forest plot for depression.

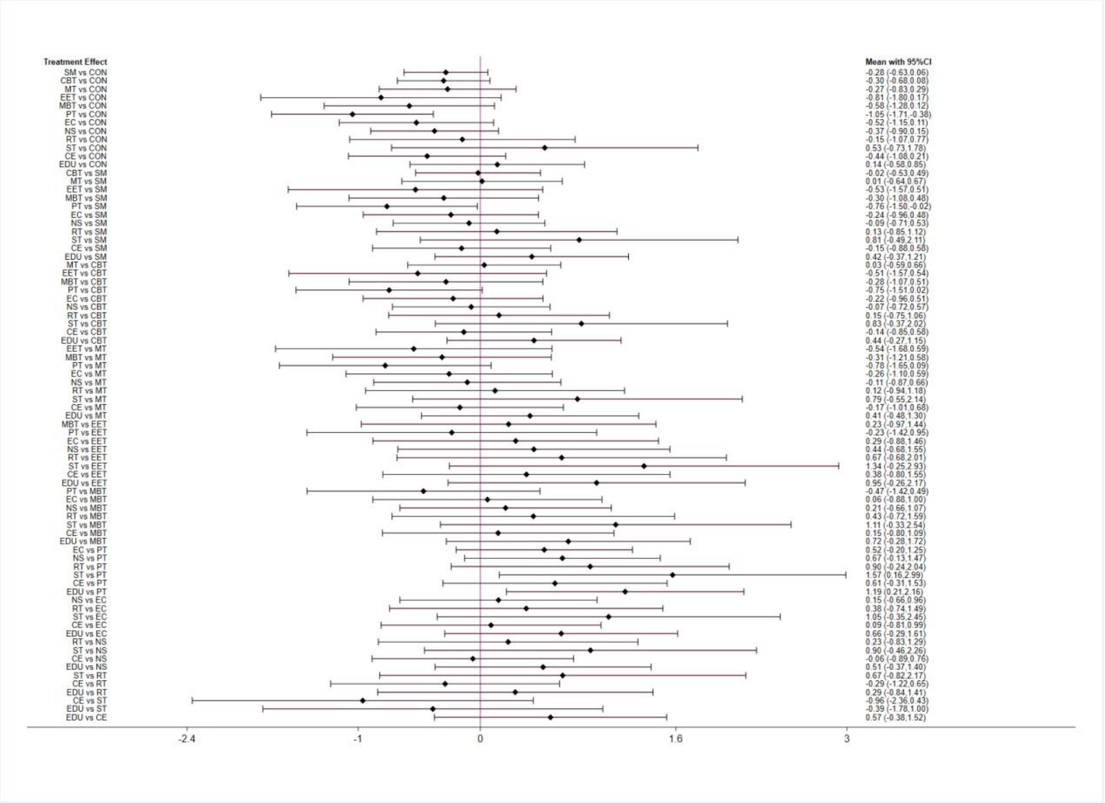

Notes: **CON**：Control group **SM**：Self-management **CBT**：Cognitive-behavioral therapy **MT**：Multi-component intervention **EET**：Enhanced education therapy **MBT**：Mind-body therapy **PT**：Psychotherapy **EC**：Enhanced care **NS**：Neurostimulation **RT**：Relaxation therapy **ST**：Supportive therapy **CE**：Conventional Exercise **EDU**：Education

Supplementary Table S4.2.1 – Forest plot for depression in adolescents

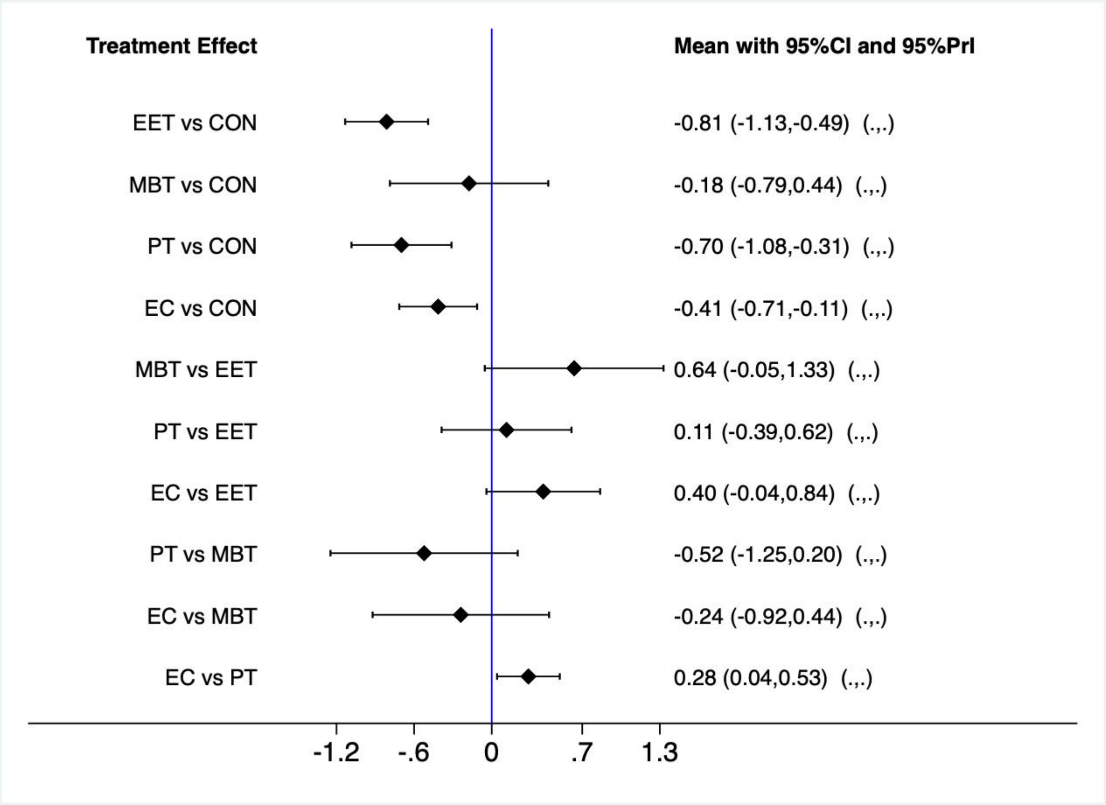

Notes: CON：Control group **EET**：Enhanced education therapy **MBT**：Mind-body therapy **PT**：Psychotherapy **EC**：Enhanced care

Supplementary Table S4.2.2 – Forest plot for depression in adults

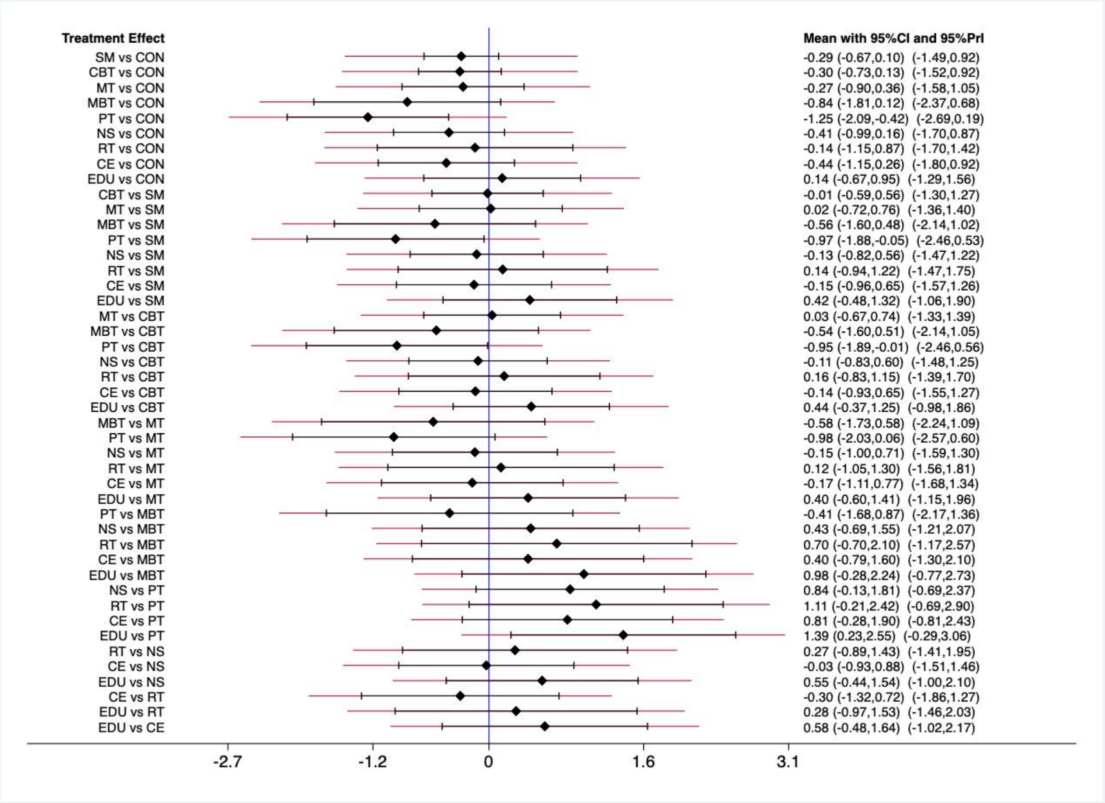

Notes: CON： Control group **SM：** Self-management **CBT：** Cognitive-behavioral therapy **MT：** Multi-component intervention **MBT：** Mind-body therapy **PT：** Psychotherapy **NS：** Neurostimulation **RT：** Relaxation therapy **CE：** Conventional Exercise **EDU：** Education

Supplementary Table S4.3 – Forest plot for QoL

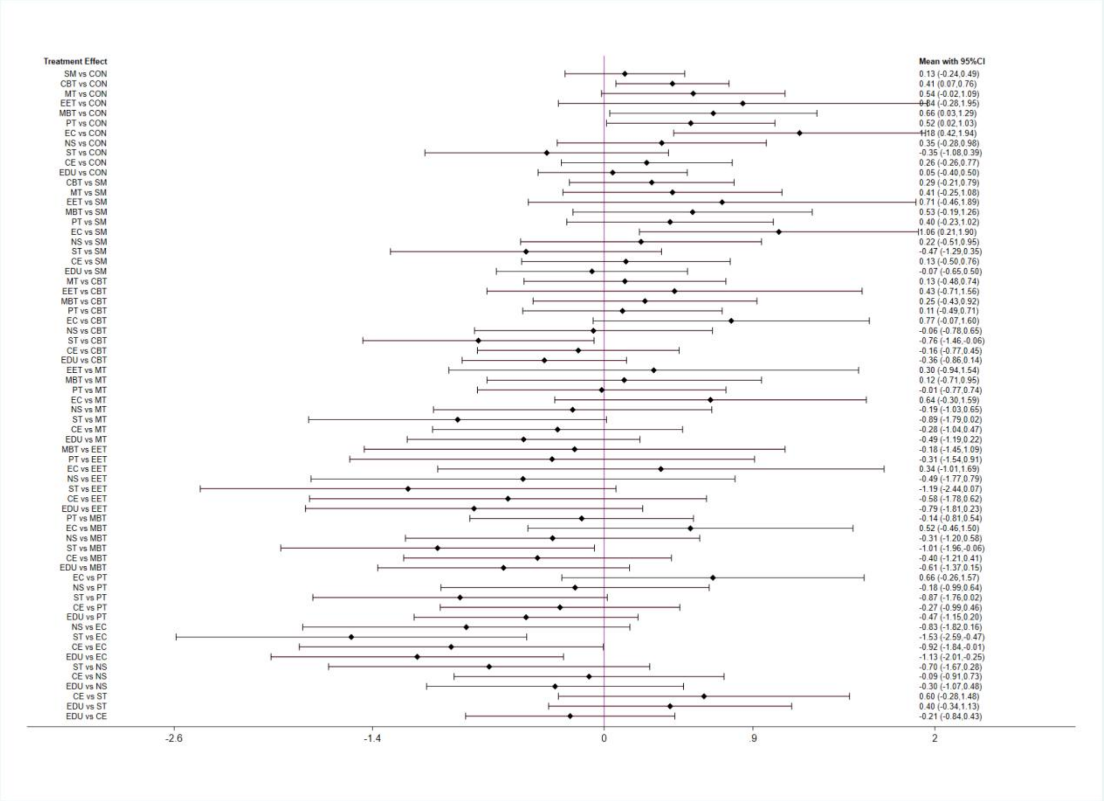

Notes: CON: Control group **SM**: Self-management **CBT**: Cognitive-behavioral therapy **MT**: Multi-component intervention **EET**: Enhanced education therapy **MBT**: Mind-body therapy **PT**: Psychotherapy **EC**: Enhanced care **NS**: Neurostimulation **ST**: Supportive therapy **CE**: Conventional Exercise **EDU**: Education

Supplementary Table S4.3.1 – Forest plot for QoL in adolescents

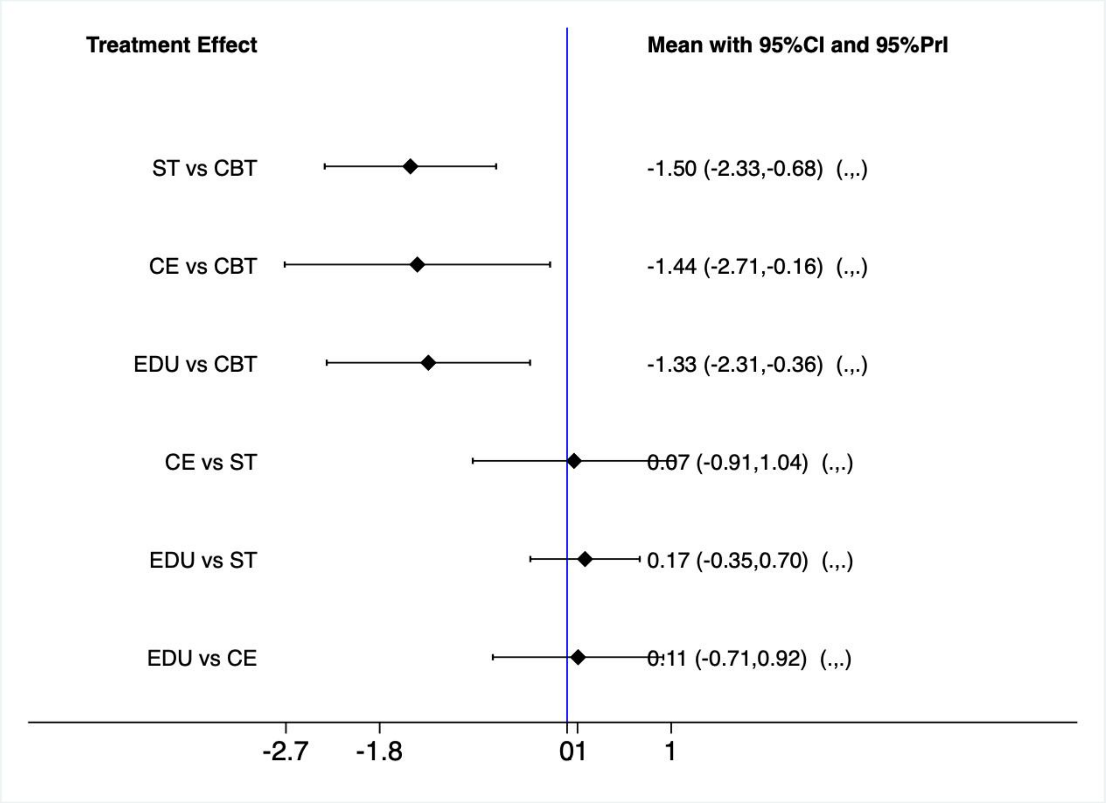

Notes: CBT： Cognitive-behavioral therapy **ST：** Supportive therapy **EC：** Enhanced care **EDU：** Education

Supplementary Table S4.3.2 – Forest plot for QoL in adults

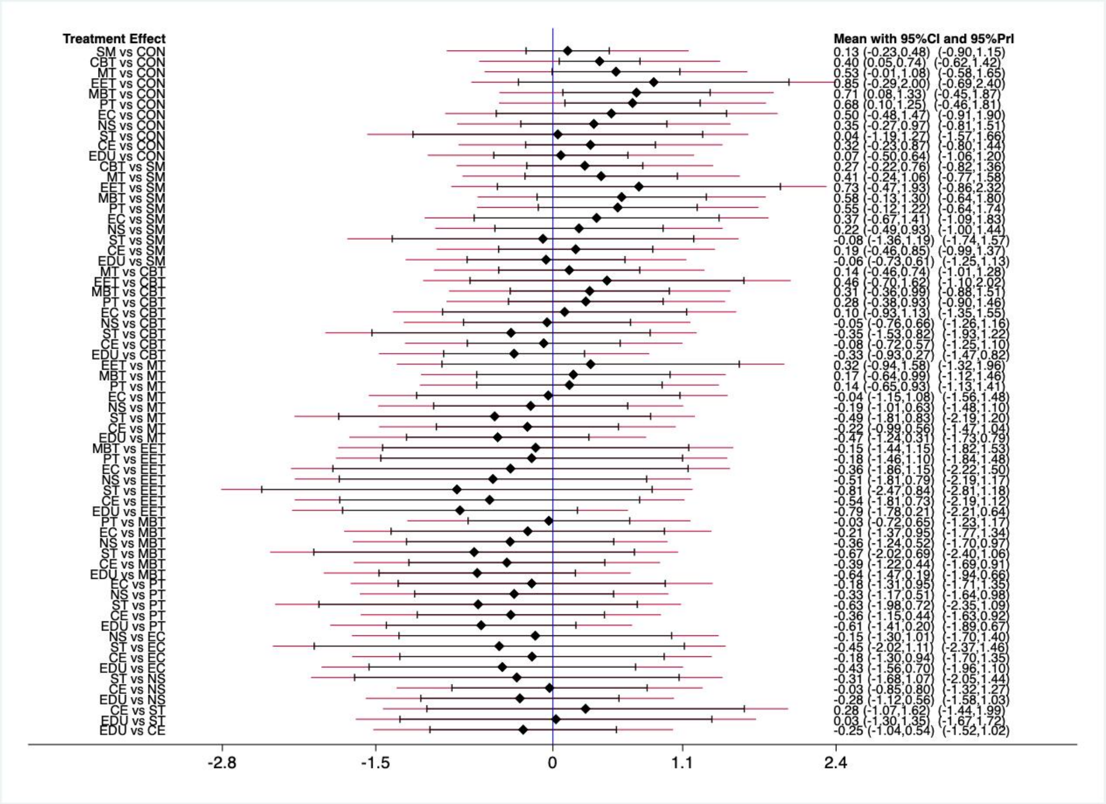

Notes: CON： Control group **SM：** Self-management **CBT：** Cognitive-behavioral therapy **MT：** Multi-component intervention **EET：** Enhanced education therapy **MBT：** Mind-body therapy **PT：** Psychotherapy **EC：** Enhanced care **NS：** Neurostimulation **ST：** Supportive therapy **CE：** Conventional Exercise **EDU：** Education

Supplementary Table S5 – League table

Supplementary Table S5.1 – Anxiety: league table

CON											
0.19 (-0.56,0.94)	SM										
0.26 (-0.35,0.87)	0.07 (-0.90,1.04)	CBT									
0.35 (-0.64,1.35)	0.16 (-1.08,1.41)	0.09 (-0.98,1.16)	MT								
1.80 (0.30,3.31)	1.61 (-0.07,3.30)	1.54 (-0.08,3.17)	1.45 (-0.35,3.26)	EET							
0.95 (-0.02,1.92)	0.76 (-0.47,1.98)	0.69 (-0.46,1.83)	0.59 (-0.80,1.98)	-0.86 (-2.65,0.93)	MBT						
1.41 (0.39,2.44)	1.22 (-0.05,2.49)	1.15 (-0.04,2.34)	1.06 (-0.37,2.49)	-0.39 (-2.21,1.43)	0.47 (-0.95,1.88)	PT					
0.76 (-0.18,1.71)	0.57 (-0.63,1.78)	0.50 (-0.62,1.63)	0.41 (-0.96,1.78)	-1.04 (-2.82,0.74)	-0.18 (-1.54,1.17)	-0.65 (-1.75,0.45)	EC				
0.10 (-0.72,0.93)	-0.09 (-1.20,1.03)	-0.16 (-1.18,0.87)	-0.25 (-1.54,1.04)	-1.70 (-3.42,0.01)	-0.84 (-2.12,0.43)	-1.31 (-2.62,-0.00)	-0.66 (-1.91,0.59)	NS			
0.10 (-1.94,2.13)	-0.09 (-2.26,2.07)	-0.16 (-2.29,1.96)	-0.26 (-2.52,2.01)	-1.71 (-4.24,0.82)	-0.85 (-3.10,1.40)	-1.32 (-3.59,0.96)	-0.67 (-2.91,1.57)	-0.01 (-2.20,2.19)	RT		
0.14 (-1.00,1.28)	-0.05 (-1.42,1.31)	-0.12 (-1.41,1.17)	-0.21 (-1.73,1.30)	-1.67 (-3.55,0.22)	-0.81 (-2.30,0.69)	-1.27 (-2.80,0.26)	-0.63 (-2.11,0.85)	0.04 (-1.37,1.44)	0.04 (-1.64,1.73)	CE	
-0.25 (-1.85,1.35)	-0.44 (-2.21,1.32)	-0.51 (-1.99,0.96)	-0.60 (-2.43,1.22)	-2.06 (-4.25,0.14)	-1.20 (-3.07,0.67)	-1.66 (-3.56,0.24)	-1.01 (-2.87,0.84)	-0.35 (-2.15,1.44)	-0.35 (-2.93,2.24)	-0.39 (-2.35,1.57)	EDU

Notes: CON： Control group **SM：** Self-management **CBT：** Cognitive-behavioral therapy **MT：** Multi-component intervention **EET：** Enhanced education therapy **MBT：** Mind-body therapy **PT：** Psychotherapy **EC：** Enhanced care **NS：** Neurostimulation **RT：** Relaxation therapy **CE：** Conventional Exercise **EDU：** Educatio

Supplementary Table S5.1.1 – Anxiety in adolescents: league table

CON			
1.80 (0.62,2.99)	EET		
-0.04 (-1.26,1.17)	-1.85 (-3.55,-0.15)	PT	
0.58 (-0.29,1.45)	-1.23 (-2.70,0.25)	0.62 (-0.88,2.12)	EC

Supplementary Table S5.1.2 – Anxiety in adults: league table

CON										
0.19 (-0.60,0.98)	SM									
0.26 (-0.38,0.90)	0.07 (-0.95,1.09)	CBT								
0.35 (-0.70,1.40)	0.16 (-1.15,1.48)	0.09 (-1.03,1.22)	MT							
0.95 (-0.07,1.96)	0.76 (-0.53,2.04)	0.69 (-0.51,1.89)	0.59 (-0.86,2.05)	MBT						
1.67 (0.39,2.96)	1.48 (-0.02,2.99)	1.41 (-0.02,2.85)	1.32 (-0.33,2.98)	0.73 (-0.91,2.36)	PT					
1.39 (-0.63,3.41)	1.20 (-0.97,3.37)	1.13 (-0.99,3.25)	1.04 (-1.24,3.31)	0.44 (-1.82,2.70)	-0.28 (-1.85,1.28)	EC				
0.11 (-0.75,0.97)	-0.08 (-1.25,1.09)	-0.15 (-1.22,0.93)	-0.24 (-1.60,1.12)	-0.83 (-2.16,0.50)	-1.56 (-3.10,-0.02)	-1.28 (-3.47,0.92)	NS			
0.10 (-2.02,2.22)	-0.09 (-2.36,2.17)	-0.16 (-2.38,2.06)	-0.25 (-2.62,2.11)	-0.85 (-3.20,1.51)	-1.57 (-4.05,0.90)	-1.29 (-4.22,1.64)	-0.01 (-2.30,2.28)	RT		
0.14 (-1.05,1.33)	-0.05 (-1.48,1.38)	-0.12 (-1.47,1.24)	-0.21 (-1.80,1.38)	-0.81 (-2.37,0.76)	-1.53 (-3.28,0.22)	-1.25 (-3.59,1.10)	0.03 (-1.44,1.50)	0.04 (-1.71,1.80)	CE	
-0.25 (-1.94,1.43)	-0.44 (-2.30,1.42)	-0.51 (-2.07,1.05)	-0.60 (-2.53,1.32)	-1.20 (-3.16,0.77)	-1.92 (-4.04,0.19)	-1.64 (-4.27,0.99)	-0.36 (-2.26,1.53)	-0.35 (-3.06,2.36)	-0.39 (-2.46,1.67)	EDU

Notes:CON： Control group **SM：** Self-management **CBT：** Cognitive-behavioral therapy **MT：** Multi-component intervention **EET：** Enhanced education therapy **MBT：** Mind-body therapy **PT：** Psychotherapy **EC：** Enhanced care **NS：** Neurostimulation **ST：** Supportive therapy **CE：** Conventional Exercise **EDU：** Education

Supplementary Table S5.2 — Depression: league table

CON												
0.28 (-0.06,0.63)	SM											
0.30 (-0.08,0.68)	0.02 (-0.49,0.53)	CBT										
0.27 (-0.29,0.83)	-0.01 (-0.67,0.64)	-0.03 (-0.66,0.59)	MT									
0.81 (-0.17,1.80)	0.53 (-0.51,1.57)	0.51 (-0.54,1.57)	0.54 (-0.59,1.68)	EET								
0.58 (-0.12,1.28)	0.30 (-0.48,1.08)	0.28 (-0.51,1.07)	0.31 (-0.58,1.21)	-0.23 (-1.44,0.97)	MBT							
1.05 (0.38,1.71)	0.76 (0.02,1.50)	0.75 (-0.02,1.51)	0.78 (-0.09,1.65)	0.23 (-0.95,1.42)	0.47 (-0.49,1.42)	PT						
0.52 (-0.11,1.15)	0.24 (-0.48,0.96)	0.22 (-0.51,0.96)	0.26 (-0.59,1.10)	-0.29 (-1.46,0.88)	-0.06 (-1.00,0.88)	-0.52 (-1.25,0.20)	EC					
0.37 (-0.15,0.90)	0.09 (-0.53,0.71)	0.07 (-0.57,0.72)	0.11 (-0.66,0.87)	-0.44 (-1.55,0.68)	-0.21 (-1.07,0.66)	-0.67 (-1.47,0.13)	-0.15 (-0.96,0.66)	NS				
0.15 (-0.77,1.07)	-0.13 (-1.12,0.85)	-0.15 (-1.06,0.75)	-0.12 (-1.18,0.94)	-0.67 (-2.01,0.68)	-0.43 (-1.59,0.72)	-0.90 (-2.04,0.24)	-0.38 (-1.49,0.74)	-0.23 (-1.29,0.83)	RT			
-0.53 (-1.78,0.73)	-0.81 (-2.11,0.49)	-0.83 (-2.02,0.37)	-0.79 (-2.14,0.55)	-1.34 (-2.93,0.25)	-1.11 (-2.54,0.33)	-1.57 (-2.99,-0.16)	-1.05 (-2.45,0.35)	-0.90 (-2.26,0.46)	-0.67 (-2.17,0.82)	ST		
0.44 (-0.21,1.08)	0.15 (-0.58,0.88)	0.14 (-0.58,0.85)	0.17 (-0.68,1.01)	-0.38 (-1.55,0.80)	-0.15 (-1.09,0.80)	-0.61 (-1.53,0.31)	-0.09 (-0.99,0.81)	0.06 (-0.76,0.89)	0.29 (-0.65,1.22)	0.96 (-0.43,2.36)	CE	
-0.14 (-0.85,0.58)	-0.42 (-1.21,0.37)	-0.44 (-1.15,0.27)	-0.41 (-1.30,0.48)	-0.95 (-2.17,0.26)	-0.72 (-1.72,0.28)	-1.19 (-2.16,-0.21)	-0.66 (-1.61,0.29)	-0.51 (-1.40,0.37)	-0.29 (-1.41,0.84)	0.39 (-1.00,1.78)	-0.57 (-1.52,0.38)	EDU

Notes:CON： Control group **SM：** Self-management **CBT：** Cognitive-behavioral therapy **MT：** Multi-component intervention **EET：** Enhanced education therapy **MBT：** Mind-body therapy **PT：** Psychotherapy **EC：** Enhanced care **NS：** Neurostimulation **RT：** Relaxation therapy **ST：** Supportive therapy **CE：** Conventional Exercise **EDU：** Education

Supplementary Table S5.2.1 — Depression in adolescents: league table

CON				
0.81 (0.49,1.13)	EET			
0.18 (-0.44,0.79)	-0.64 (-1.33,0.05)	MBT		
0.70 (0.31,1.08)	-0.11 (-0.62,0.39)	0.52 (-0.20,1.25)	PT	
0.41 (0.11,0.71)	-0.40 (-0.84,0.04)	0.24 (-0.44,0.92)	-0.28 (-0.53,-0.04)	EC

Supplementary Table S5.2.2 — Depression in adults: league table

CON									
0.29 (-0.10,0.67)	SM								
0.30 (-0.13,0.73)	0.01 (-0.56,0.59)	CBT							
0.27 (-0.36,0.90)	-0.02 (-0.76,0.72)	-0.03 (-0.74,0.67)	MT						
0.84 (-0.12,1.81)	0.56 (-0.48,1.60)	0.54 (-0.51,1.60)	0.58 (-0.58,1.73)	MBT					
1.25 (0.42,2.09)	0.97 (0.05,1.88)	0.95 (0.01,1.89)	0.98 (-0.06,2.03)	0.41 (-0.87,1.68)	PT				
0.41 (-0.16,0.99)	0.13 (-0.56,0.82)	0.11 (-0.60,0.83)	0.15 (-0.71,1.00)	-0.43 (-1.55,0.69)	-0.84 (-1.81,0.13)	NS			
0.14 (-0.87,1.15)	-0.14 (-1.22,0.94)	-0.16 (-1.15,0.83)	-0.12 (-1.30,1.05)	-0.70 (-2.10,0.70)	-1.11 (-2.42,0.21)	-0.27 (-1.43,0.89)	RT		
0.44 (-0.26,1.15)	0.15 (-0.65,0.96)	0.14 (-0.65,0.93)	0.17 (-0.77,1.11)	-0.40 (-1.60,0.79)	-0.81 (-1.90,0.28)	0.03 (-0.88,0.93)	0.30 (-0.72,1.32)	CE	
-0.14 (-0.95,0.67)	-0.42 (-1.32,0.48)	-0.44 (-1.25,0.37)	-0.40 (-1.41,0.60)	-0.98 (-2.24,0.28)	-1.39 (-2.55,-0.23)	-0.55 (-1.54,0.44)	-0.28 (-1.53,0.97)	-0.58 (-1.64,0.48)	EDU

Notes:CON： Control group **SM：** Self-management **CBT：** Cognitive-behavioral therapy **MT：** Multi-component intervention **EET：** Enhanced education therapy **MBT：** Mind-body therapy **PT：** Psychotherapy **EC：** Enhanced care **NS：** Neurostimulation **ST：** Supportive therapy **CE：** Conventional Exercise **EDU：** Education

Supplementary Table S5.3 – QoL: league table

CON												
-0.13 (-0.49,0.24)	SM											
-0.41 (-0.76,-0.07)	-0.29 (-0.79,0.21)	CBT										
-0.54 (-1.09,0.02)	-0.41 (-1.08,0.25)	-0.13 (-0.74,0.48)	MT									
-0.84 (-1.95,0.28)	-0.71 (-1.89,0.46)	-0.43 (-1.56,0.71)	-0.30 (-1.54,0.94)	EET								
-0.66 (-1.29,-0.03)	-0.53 (-1.26,0.19)	-0.25 (-0.92,0.43)	-0.12 (-0.95,0.71)	0.18 (-1.09,1.45)	MBT							
-0.52 (-1.03,-0.02)	-0.40 (-1.02,0.23)	-0.11 (-0.71,0.49)	0.01 (-0.74,0.77)	0.31 (-0.91,1.54)	0.14 (-0.54,0.81)	PT						
-1.18 (-1.94,-0.42)	-1.06 (-1.90,-0.21)	-0.77 (-1.60,0.07)	-0.64 (-1.59,0.30)	-0.34 (-1.69,1.01)	-0.52 (-1.50,0.46)	-0.66 (-1.57,0.26)	EC					
-0.35 (-0.98,0.28)	-0.22 (-0.95,0.51)	0.06 (-0.65,0.78)	0.19 (-0.65,1.03)	0.49 (-0.79,1.77)	0.31 (-0.58,1.20)	0.18 (-0.64,0.99)	0.83 (-0.16,1.82)	NS				
0.35 (-0.39,1.08)	0.47 (-0.35,1.29)	0.76 (0.06,1.46)	0.89 (-0.02,1.79)	1.19 (-0.07,2.44)	1.01 (0.06,1.96)	0.87 (-0.02,1.76)	1.53 (0.47,2.59)	0.70 (-0.28,1.67)	ST			
-0.26 (-0.77,0.26)	-0.13 (-0.76,0.50)	0.16 (-0.45,0.77)	0.28 (-0.47,1.04)	0.58 (-0.62,1.78)	0.40 (-0.41,1.21)	0.27 (-0.46,0.99)	0.92 (0.01,1.84)	0.09 (-0.73,0.91)	-0.60 (-1.48,0.28)	CE		
-0.05 (-0.50,0.40)	0.07 (-0.50,0.65)	0.36 (-0.14,0.86)	0.49 (-0.22,1.19)	0.79 (-0.23,1.81)	0.61 (-0.15,1.37)	0.47 (-0.20,1.15)	1.13 (0.25,2.01)	0.30 (-0.48,1.07)	-0.40 (-1.13,0.34)	0.21 (-0.43,0.84)	EDU	

Notes:CON： Control group **SM**： Self-management **CBT**： Cognitive-behavioral therapy **MT**： Multi-component intervention **EET**： Enhanced education therapy
MBT： Mind-body therapy **PT**： Psychotherapy **EC**： Enhanced care **NS**： Neurostimulation **ST**： Supportive therapy **CE**： Conventional Exercise **EDU**： Education

Supplementary Table S5.3.1 – QoL in adolescents: league table

CBT			
1.50 (0.68,2.33)	ST		
1.44 (0.16,2.71)	-0.07 (-1.04,0.91)	CE	
1.33 (0.36,2.31)	-0.17 (-0.70,0.35)	-0.11 (-0.92,0.71)	EDU

Supplementary Table S5.3.2 – QoL in adults: league table

CON										
-0.13 (-0.48,0.23)	SM									
-0.40 (-0.74,-0.05)	-0.27 (-0.76,0.22)	CBT								
-0.53 (-1.08,0.01)	-0.41 (-1.06,0.24)	-0.14 (-0.74,0.46)	MT							
-0.85 (-2.00,0.29)	-0.73 (-1.93,0.47)	-0.46 (-1.62,0.70)	-0.32 (-1.58,0.94)	EET						
-0.71 (-1.33,-0.08)	-0.58 (-1.30,0.13)	-0.31 (-0.99,0.36)	-0.17 (-0.99,0.64)	0.15 (-1.15,1.44)	MBT					
-0.68 (-1.25,-0.10)	-0.55 (-1.22,0.12)	-0.28 (-0.93,0.38)	-0.14 (-0.93,0.65)	0.18 (-1.10,1.46)	0.03 (-0.65,0.72)	PT				
-0.50 (-1.47,0.48)	-0.37 (-1.41,0.67)	-0.10 (-1.13,0.93)	0.04 (-1.08,1.15)	0.36 (-1.15,1.86)	0.21 (-0.95,1.37)	0.18 (-0.95,1.31)	EC			
-0.35 (-0.97,0.27)	-0.22 (-0.93,0.49)	0.05 (-0.66,0.76)	0.19 (-0.63,1.01)	0.51 (-0.79,1.81)	0.36 (-0.52,1.24)	0.33 (-0.51,1.17)	0.15 (-1.01,1.30)	NS		
-0.04 (-1.27,1.19)	0.08 (-1.19,1.36)	0.35 (-0.82,1.53)	0.49 (-0.83,1.81)	0.81 (-0.84,2.47)	0.67 (-0.69,2.02)	0.63 (-0.72,1.98)	0.45 (-1.11,2.02)	0.31 (-1.07,1.68)	ST	
-0.32 (-0.87,0.23)	-0.19 (-0.85,0.46)	0.08 (-0.57,0.72)	0.22 (-0.56,0.99)	0.54 (-0.73,1.81)	0.39 (-0.44,1.22)	0.36 (-0.44,1.15)	0.18 (-0.94,1.30)	0.03 (-0.80,0.85)	-0.28 (-1.62,1.07)	CE

Notes:CON：Control group **SM**：Self-management **CBT**：Cognitive-behavioral therapy **MT**：Multi-component intervention **EET**：Enhanced education therapy **MBT**：Mind-body therapy **PT**：Psychotherapy **EC**：Enhanced care **NS**：Neurostimulation **ST**：Supportive therapy **CE**：Conventional Exercise **EDU**：Education

Supplementary Table S6 – Sucra Score and Ranking

Supplementary Table S6.1 – Sucra Score and Ranking

Anxiety (28 studies, *N* = 3554)			Depression (40 studies, *N* = 4422)			Quality of Life (43 studies, *N* = 4823)		
Treament	SUCRA	Mean Rank	Treament	SUCRA	Mean Rank	Treament	SUCRA	Mean Rank
EET	91.5	1.9	PT	92.4	1.9	EC	93.1	1.8
PT	86.9	2.4	EET	78.2	3.6	EET	76.2	3.6
MBT	72.7	4	MBT	68.7	4.8	MBT	72.7	4
EC	65.2	4.8	EC	64.8	5.2	MT	64.9	4.9
MT	46.6	6.9	CE	59	5.9	PT	64.3	4.9
CBT	42.3	7.4	NS	54.8	6.4	CBT	56.6	5.8
RT	38.5	7.8	CBT	49.3	7.1	NS	49.9	6.5
SM	38	7.8	SM	47	7.4	CE	42.8	7.3

CE	36.1	8	MT	46.1	7.5	SM	30.9	8.6
NS	33.3	8.3	RT	38.5	8.4	EDU	24.4	9.3
CON	25.1	9.2	CON	20.3	10.6	CON	17.7	10.1
EDU	24	9.4	EDU	19.3	10.7	ST	6.6	11.3
			ST	11.6	11.6			

Notes: CON：Control group **SM**：Self-management **CBT**：Cognitive-behavioral therapy **MT**：Multi-component intervention **EET**：Enhanced education therapy **MBT**：Mind-body therapy **PT**：Psychotherapy **EC**：Enhanced care **NS**：Neurostimulation **RT**：Relaxation therapy **ST**：Supportive therapy **CE**：Conventional Exercise **EDU**：Education

Supplementary Table S6 – Sucra Score and Ranking in adolescents

Anxiety			Depression			Quality of Life		
Treament	SUCRA	Mean Rank	Treament	SUCRA	Mean Rank	Treament	SUCRA	Mean Rank
EET	97.6	1.1	EET	89.9	1.4	CBT	99.5	1.0
EC	58.4	2.2	PT	81.0	1.8	EDU	44.4	2.7
PT	22.7	3.3	EC	45.0	3.2	CE	32.3	3.0
CON	21.3	3.4	MBT	26.6	3.9	ST	23.8	3.3
			CON	0.0	4.7			

Notes: CON： Control group **SM**： Self-management **CBT**： Cognitive-behavioral therapy **MT**： Multi-component intervention **EET**： Enhanced education therapy **MBT**： Mind-body therapy **PT**： Psychotherapy **EC**： Enhanced care **NS**： Neurostimulation **RT**： Relaxation therapy **ST**： Supportive therapy **CE**： Conventional Exercise **EDU**： Education

Supplementary Table S6.1 – Sucra Score and Ranking in adults

Anxiety			Depression			Quality of Life		
Treament	SUCRA	Mean Rank	Treament	SUCRA	Mean Rank	Treament	SUCRA	Mean Rank
PT	91.3	1.9	PT	94.3	1.5	EET	77.5	3.5
EC	79.1	3.1	MBT	78.6	2.9	MBT	76.1	3.6
MBT	74.2	3.6	CE	57.6	4.8	PT	74.3	3.8
MT	49.2	6.1	NS	56.7	4.9	MT	64.7	4.9
CBT	45.2	6.5	CBT	48.7	5.6	EC	58.0	5.6
SM	40.9	6.9	SM	47.0	5.8	CBT	53.9	6.1
RT	39.4	7.1	MT	45.0	6.0	NS	49.0	6.6
CE	39.0	7.1	RT	47.0	6.7	CE	46.5	6.9
CON	36.1	8.0	CON	17.9	8.4	ST	31.4	8.5
EDU	26.2	8.4	EDU	17.6	8.4	SM	28.6	8.8

	EDU	24.6	9.3	
	CON	15.3	10.3	

Notes: CON：Control group **SM**：Self-management **CBT**：Cognitive-behavioral therapy **MT**：Multi-component intervention **EET**：Enhanced education therapy **MBT**：Mind-body therapy **PT**：Psychotherapy **EC**：Enhanced care **NS**：Neurostimulation **RT**：Relaxation therapy **ST**：Supportive therapy **CE**：Conventional Exercise **EDU**：Education

Supplementary Table S6.2 – Ranking Probability for anxiety

Anxiety	Intervention											
Rank	CON	SM	CBT	MT	EET	MBT	PT	EC	NS	RT	CE	EDU
Best	0.0	0.1	0.0	0.7	59.5	6.4	25.7	1.9	0.1	4.6	0.3	0.7
2nd	0.0	0.6	0.3	3.3	18.9	18.5	38.6	9.1	0.6	6.8	1.3	2.0
3rd	0.0	2.3	1.6	7.1	9.2	23.2	18.8	21.0	2.1	7.2	4.1	3.5
4th	0.0	5.8	5.6	10.8	5.2	19.8	8.5	21.5	4.3	7.4	7.0	4.2
5th	0.4	10.2	11.1	14.0	2.8	12.4	4.1	15.4	8.0	7.4	9.3	5.0
6th	2.3	13.0	17.1	13.1	1.5	7.8	2.0	10.6	10.6	6.3	10.4	5.4
7th	8.2	13.7	18.9	11.4	1.0	4.6	1.1	7.3	12.0	5.7	10.0	6.1
8th	18.7	13.6	16.0	9.5	0.7	3.2	0.7	5.0	12.3	5.2	9.3	5.9
9th	26.5	12.5	13.2	8.4	0.4	1.8	0.3	3.3	12.4	5.1	10.1	6.0
10th	24.9	11.7	9.3	8.1	0.3	1.3	0.1	2.5	13.2	6.8	12.8	8.9
11th	14.4	10.7	5.6	8.0	0.3	0.6	0.0	1.7	14.2	13.1	16.7	14.7
Worst	4.6	5.9	1.4	5.6	0.2	0.4	0.0	0.7	10.3	24.4	8.7	37.8

Notes: CON：Control group **SM**：Self-management **CBT**：Cognitive-behavioral therapy **MT**：Multi-component intervention **EET**：Enhanced education therapy **MBT**：Mind-body therapy **PT**：Psychotherapy **EC**：Enhanced care **NS**：Neurostimulation **RT**：Relaxation therapy **CE**：Conventional Exercise **EDU**：Education

Supplementary Table S6.2.1 – Ranking Probability for anxiety in adolescents

Anxiety	Intervention			
Rank	CON	EET	PT	EC
Best	0.0	93.5	1.4	5.1
2nd	5.5	5.8	18.0	70.7
3rd	52.8	0.6	28.0	18.6
Worst	41.7	0.1	52.6	5.6

Notes: CON： Control group **EET**： Enhanced education therapy **PT:** Psychotherapy **EC**： Enhanced care

Supplementary Table S6.2.2 – Ranking Probability for anxiety in adults

Anxiety	Intervention										
Rank	CON	SM	CBT	MT	MBT	PT	EC	NS	RT	CE	EDU
Best	0.0	0.3	0.1	2.0	11.2	45.8	31.0	0.5	6.7	0.7	1.7
2nd	0.0	1.5	1.0	4.3	16.7	36.7	27.9	1.2	5.9	2.4	2.5
3rd	0.0	5.1	4.6	10.8	30.4	10.1	13.7	4.6	9.7	6.3	4.8
4th	0.6	10.5	10.8	15.2	17.6	3.7	7.4	9.2	8.7	10.4	5.9
5th	2.5	13.1	17.1	14.1	9.8	1.8	4.7	11.5	7.3	12.2	6
6th	8.8	14.3	18.7	12.6	5.8	0.8	3.3	11.9	6.0	11.1	6.7
7th	18.6	13.7	17.5	10.1	3.6	0.4	2.6	12.8	5.4	9.5	5.9
8th	26.2	12.8	12.9	9.0	2.2	0.4	2.3	12.9	5.1	10.1	6.1
9th	24.7	11.9	9.7	8.8	1.3	0.2	2.3	13.2	6.3	12.9	8.8
10th	14.8	10.8	6.1	7.8	1.0	0.1	2.6	13.1	12.5	16.3	15
Worst	3.8	6.1	1.6	5.2	0.4	0.0	2.3	9.2	26.4	8.3	36.8

Notes: CON： Control group **SM：** Self-management **CBT：** Cognitive-behavioral therapy **MT：** Multi-component intervention **MBT：** Mind-body therapy **PT：** Psychotherapy **EC：** Enhanced care **NS：** Neurostimulation **RT：** Relaxation therapy **CE：** Conventional Exercise **EDU：** Education

Supplementary Table S6.3 – Ranking Probability for depression

Depression	Intervention												
Rank	CON	SM	CBT	MT	EET	MBT	PT	EC	NS	RT	ST	CE	EDU
Best	0	0.1	0.1	0.6	29.7	8.8	51.6	2.6	0.8	2.2	0.5	2.9	0.2
2nd	0	0.6	1	2.7	21	16.1	27.6	12.5	4.5	4.1	0.9	8.4	0.5
3rd	0	2.7	3	5.7	13	17.7	10.7	17.7	9.3	5.9	1.4	12	0.9
4th	0	6.9	7.2	8.2	8.5	13.4	4.9	15.7	12.3	6.4	1.5	13.6	1.6
5th	0	10.3	11.8	10.1	5.8	10.4	2.4	12.6	13.6	6.7	1.6	12.8	2
6th	0.1	14.4	15.6	11	4.8	7.7	1.2	9.5	13.5	6.3	1.8	11.1	2.9
7th	0.7	16.5	17.5	11.6	3.9	7	0.8	8	12.1	6.6	2.1	9.7	3.5
8th	3.8	17.1	17.4	12.2	3.3	5.7	0.4	6.8	10	7.3	2.6	8.3	5.2
9th	13	14.5	14.2	10.9	2.8	4.6	0.2	5.4	9.4	8.1	2.7	7.5	6.7
10th	27.6	9.8	8.4	10	2.4	3.8	0.1	4.2	6.6	8.7	3.6	5.7	9.1
11th	33.6	4.9	3.1	9.4	2.2	2.5	0	2.6	4.7	12	5.2	4.7	15.2
12th	17.9	2	0.7	5.9	2	1.9	0	1.7	2.5	16.8	12.7	2.6	33.3
Worst	3.4	0.3	0.1	1.9	0.6	0.4	0	0.6	0.7	8.9	63.4	0.7	19.1

Notes:CON: Control group **SM**: Self-management **CBT**: Cognitive-behavioral therapy **MT**: Multi-component intervention **EET**: Enhanced education therapy **MBT**: Mind-body therapy **PT**: Psychotherapy **EC**: Enhanced care **NS**: Neurostimulation **ST**: Supportive therapy **CE**: Conventional Exercise **EDU**: Education

Supplementary Table S6.3.1 – Ranking Probability for depression in adolescents

Anxiety	Intervention				
Rank	CON	EET	MBT	PT	EC
Best	0.0	65.7	2.1	32.1	0.0
2nd	0.0	28.8	6.8	60.1	4.3
3rd	0.1	5.0	15.8	7.4	71.7
4th	29.4	0.5	46.1	0.3	23.7
Worst	70.5	0.0	29.3	0.0	0.3

Notes: CON： Control group **EET**： Enhanced education therapy **MBT**： Mind-body therapy **PT**： Psychotherapy **EC**： Enhanced care

Supplementary Table S6.3.2 – Ranking Probability for depression in adults

Depression	Intervention									
Rank	CON	SM	CBT	MT	MBT	PT	NS	RT	CE	EDU
Best	0.0	0.2	0.2	0.8	23.6	68.2	1.3	2.4	3.0	0.1
2nd	0.0	2.6	2.7	5.4	36.4	21.8	9.8	6.7	13.3	1.3
3rd	0.0	9.4	8.7	11.6	13.0	4.9	19.4	9.3	21.0	2.7
4th	0.0	15.1	15.8	12.5	7.5	2.2	18.0	9.3	15.8	3.8
5th	0.5	18.7	20.2	13.2	5.6	1.3	15.4	8.0	12.5	4.5
6th	3.8	19.1	20.9	14.0	4.4	0.7	12.3	8.5	10.2	6.2
7th	14.4	16.4	16.3	13.1	3.3	0.5	10.0	8.5	9.3	8.1
8th	31.6	11.2	10.1	11.8	2.4	0.2	6.9	9.0	6.9	10.0
9th	35.2	5.5	4.2	10.5	2.0	0.1	4.4	15.4	5.3	17.4
Worst	14.4	1.8	0.8	7.2	1.7	0.1	2.4	23.0	2.6	46.0

Notes: CON： Control group **SM：** Self-management **CBT：** Cognitive-behavioral therapy **MT：** Multi-component intervention **MBT：** Mind-body therapy **PT：** Psychotherapy **NS：** Neurostimulation **RT：** Relaxation therapy **CE：** Conventional Exercise **EDU：** Education

Supplementary Table S6.4 – Ranking Probability for QoL

QoL	Intervention											
Rank	CON	SM	CBT	MT	EET	MBT	PT	EC	NS	ST	CE	EDU
Best	0	0	0.2	3	27.3	6.7	1.6	59.5	1.4	0	0.2	0
2nd	0	0.2	1.8	12.2	24.7	20.3	9	24.1	5.7	0.1	2	0
3rd	0	0.6	6.9	17.9	11.6	23.3	17	7.8	9.7	0.1	4.8	0.3
4th	0	1.5	14.2	17.4	7.3	16.6	20.1	3.9	10.4	0.3	7.2	0.9
5th	0	3.9	20.9	14.3	5.9	11.6	17.4	2.1	11.3	0.7	9.8	2.2
6th	0.1	7.5	23.1	12	5.4	8.1	13.1	1.2	12	1.1	12.5	4.1
7th	1.2	13	17.7	9	4.6	5.1	9.7	0.6	12.4	2	16	8.7
8th	6.1	19.3	10.1	6.4	4	3.7	5.6	0.3	11.5	3.3	15.4	14.3
9th	21	20.3	3.7	3.5	2.7	2.2	3.5	0.3	8.4	4.2	12.6	17.6
10th	35.4	16.3	1.2	2.2	2.2	1.2	1.9	0.1	6.6	5.7	8.4	18.9
11th	29.7	12.9	0.2	1.7	2.6	0.9	0.9	0	6.7	10.4	7.7	26.3
Worst	6.5	4.6	0	0.5	1.7	0.3	0.3	0	3.8	72.2	3.5	6.6

Notes: CON：Control group **SM**：Self-management **CBT**：Cognitive-behavioral therapy **MT**：Multi-component intervention **EET**：Enhanced education therapy **MBT**：Mind-body therapy **PT**：Psychotherapy **EC**：Enhanced care **NS**：Neurostimulation **RT**：Relaxation therapy **ST**：Supportive therapy **CE**：Conventional Exercise **EDU**：Education

Supplementary Table S6.4.1 – Ranking Probability for QoL in adolescents

Anxiety	Intervention			
Rank	CBT	ST	EC	EDU
Best	98.6	0.0	1.1	0.3
2nd	1.2	19.1	35.8	43.8
3rd	0.2	33.3	21.8	44.7
Worst	0.0	47.6	41.2	11.2

Notes: CBT： Cognitive-behavioral therapy **ST：** Supportive therapy **EC：** Enhanced care **EDU：** Education

Supplementary Table S6.4.2 – Ranking Probability for QoL in adults

QoL	Intervention											
Rank	CON	SM	CBT	MT	EET	MBT	PT	EC	NS	ST	CE	EDU
Best	0.0	0.0	0.3	6.3	41	16	12.6	14.3	2.7	5.3	1.4	0.0
2nd	0.0	0.2	1.9	11.8	14.3	22.6	20.6	12.7	6.2	5.2	4.0	0.5
3rd	0.0	0.6	5.9	14.9	9.0	19.2	20.6	9.7	7.8	4.3	6.7	1.3
4th	0.0	1.4	11.8	15.7	7.2	14.1	14.3	9.6	9.4	5.2	9.3	2.2
5th	0.0	3.2	18.5	14.2	5.9	9.5	10.8	8.0	11.0	4.6	10.6	3.8
6th	0.1	6.1	21.3	11.4	4.5	6.5	7.7	7.1	11.7	5.1	12.4	6.1
7th	1.0	10.9	18.3	9.4	3.8	4.9	5.4	6.9	11.8	5.5	13.6	8.6
8th	5.3	16.5	12.8	6.9	3.8	3.3	3.8	6.2	11.0	6.2	12.7	11.6
9th	15.1	20.4	6.4	4.6	2.7	1.9	2.4	5.6	9.4	6.8	11.1	13.7
10th	30.6	18.6	2.3	2.9	2.7	1.1	1.2	5.0	7.8	6.4	7.4	14
11th	33.1	14.4	0.5	1.5	2.7	0.5	0.4	6.4	6.4	8.8	5.8	19.5
Worst	14.9	7.7	0.1	0.7	2.4	0.4	0.2	8.5	4.8	36.7	5.0	18.6

Notes: CON： Control group **SM：** Self-management **CBT：** Cognitive-behavioral therapy **MT：** Multi-component intervention **EET：** Enhanced education therapy **MBT：** Mind-body therapy **PT：** Psychotherapy **EC：** Enhanced care **NS：** Neurostimulation **ST：** Supportive therapy **CE：** Conventional Exercise **EDU：** Education

Supplementary Table S7 – Contribution graph

Supplementary Table S7.1 – Contribution graph for anxiety

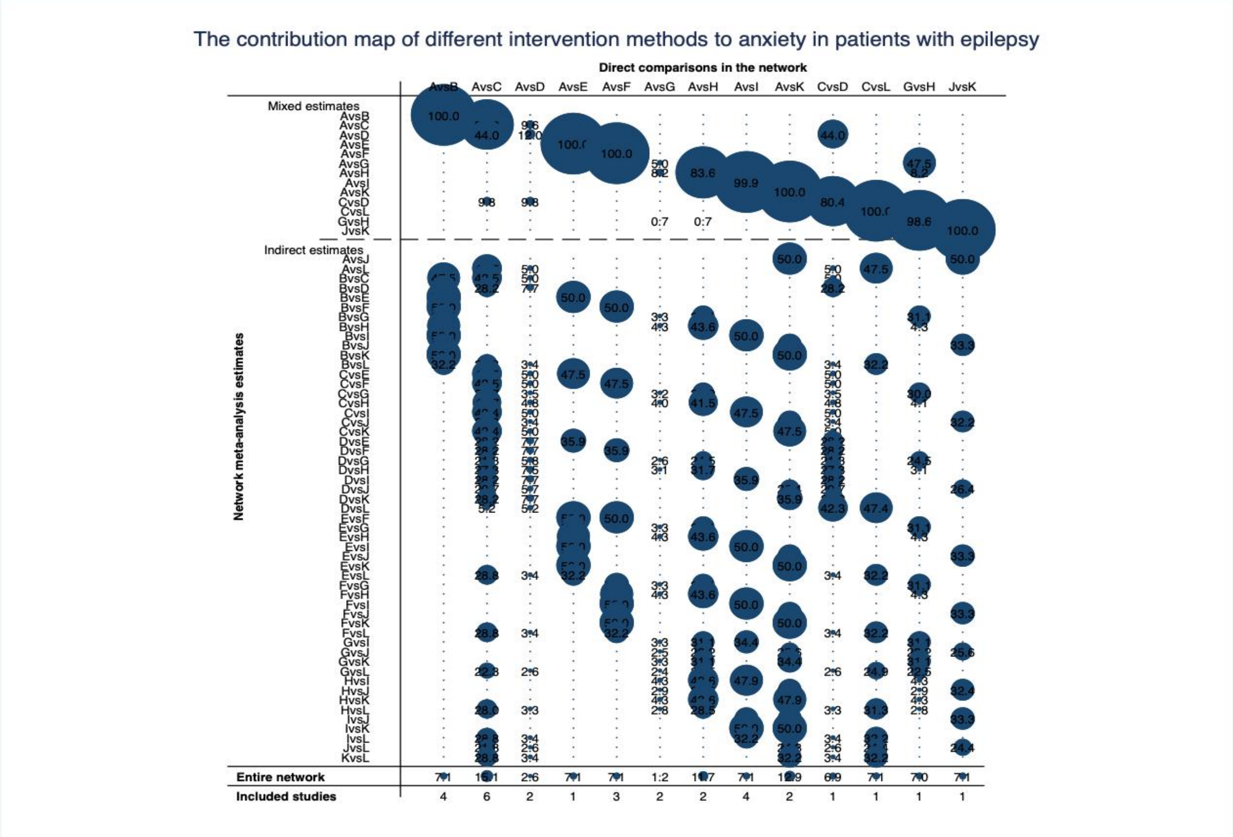

Notes: A： Control group **B：** Self-management **C：** Cognitive-behavioral therapy **D：** Multi-component intervention **E：** Enhanced education therapy **F：** Mind-body therapy **G** Psychotherapy **H：** Enhanced care **I：** Neurostimulation **J：** Relaxation therapy **K：** Conventional Exercise **L：** Education

Supplementary Table S7.1.1 – Contribution graph for anxiety in adolescents

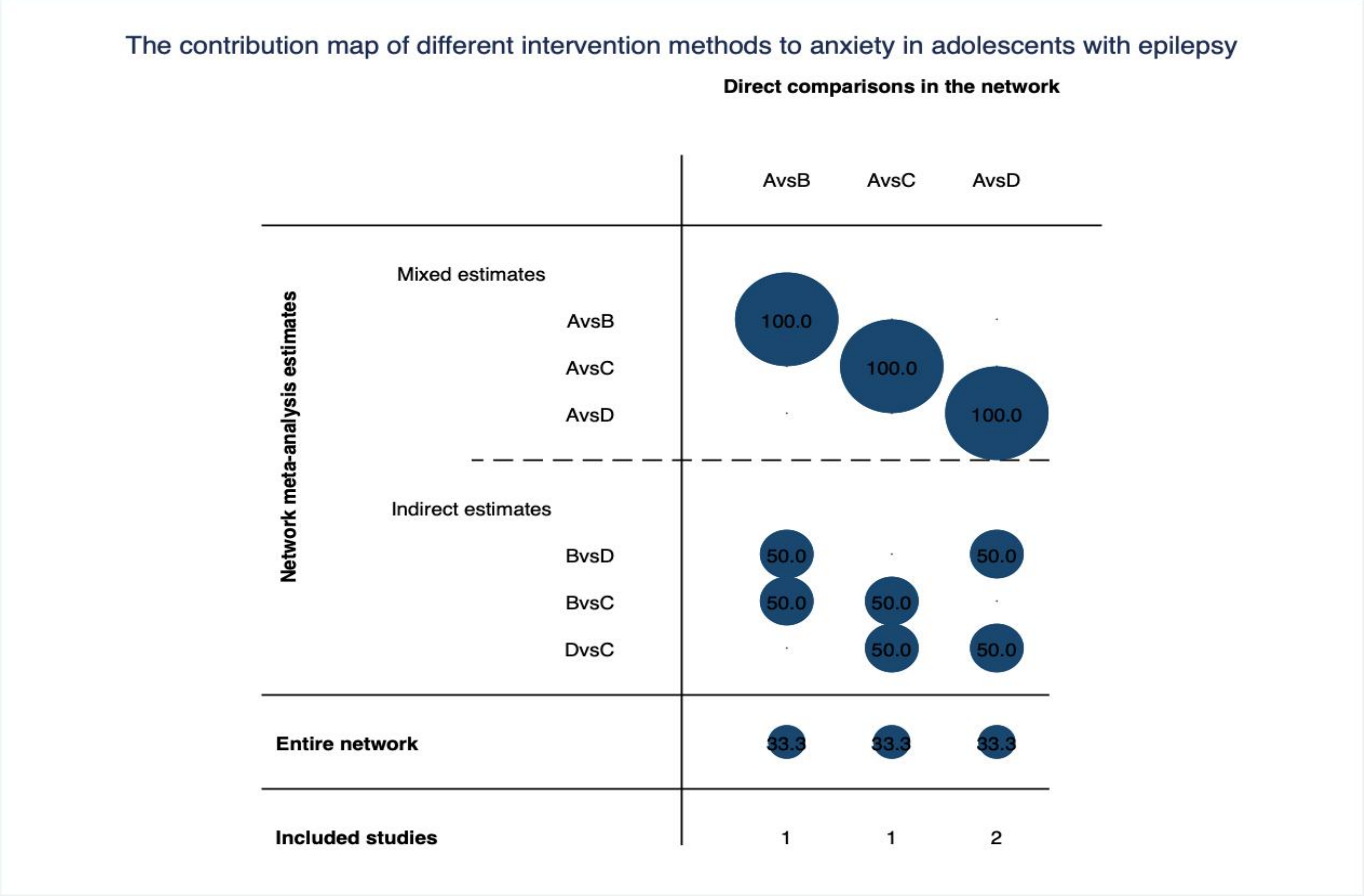

Notes: A： Control group **B：** Enhanced education therapy **C:** Psychotherapy **D：** Enhanced care

Supplementary Table S7.1.2 – Contribution graph for anxiety in adults

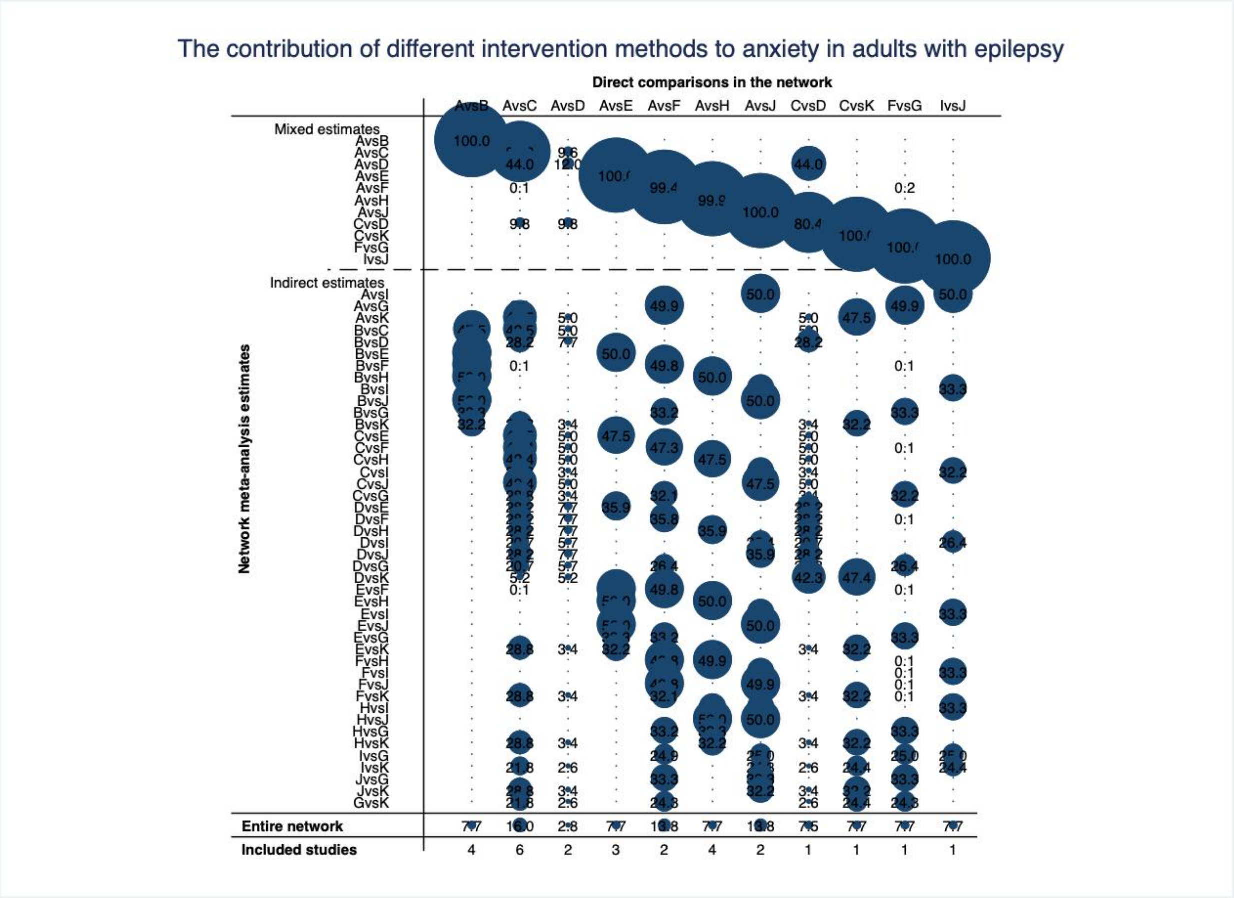

Notes: A： Control group **B：** Self-management **C：** Cognitive-behavioral therapy **D：** Multi-component intervention E：Mind-body therapy F: Psychotherapy G：Enhanced care

H： Neurostimulation I：Relaxation therapy J：Conventional Exercise **K：** Education

Supplementary Table S7.2 – Contribution graph for depression

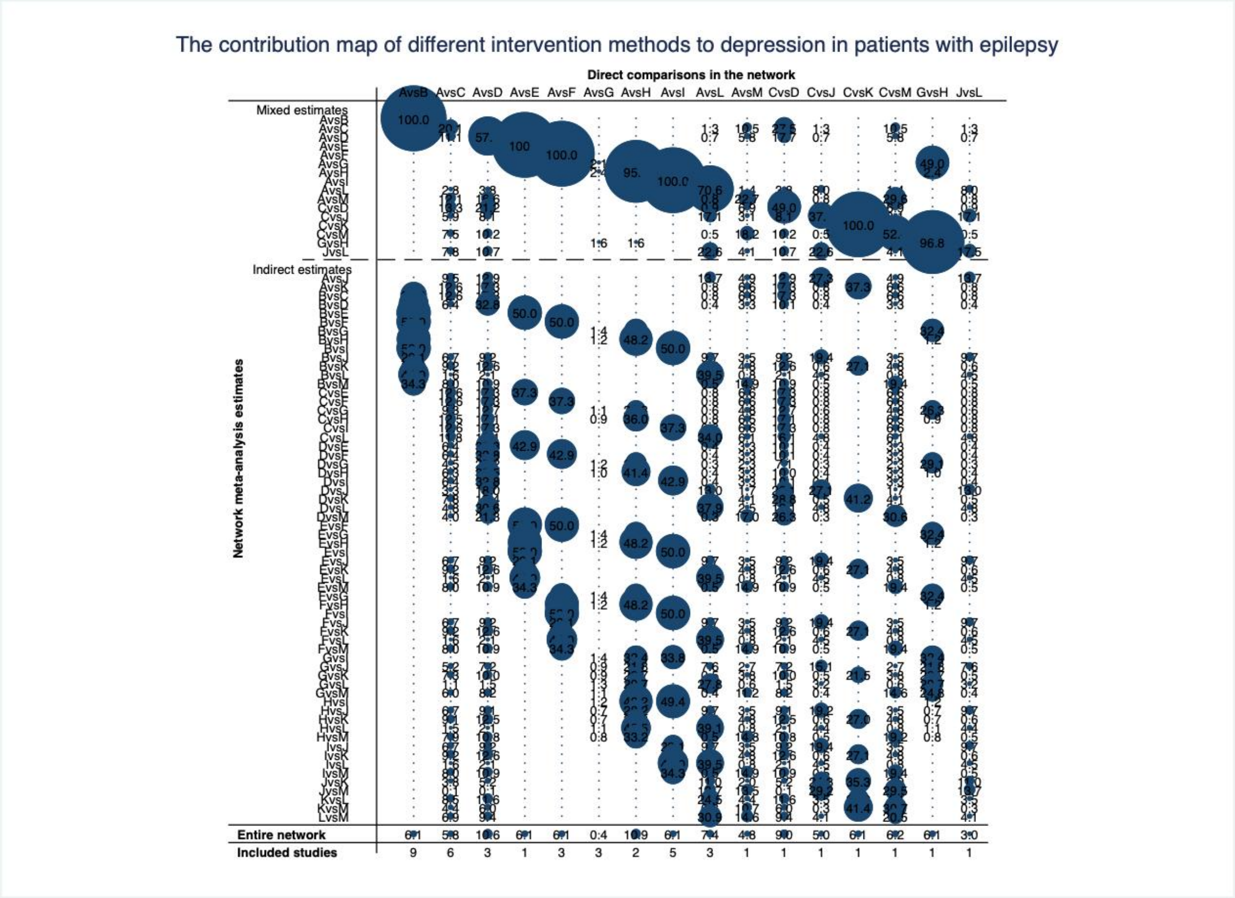

Notes: A：Control group **B：**Self-management **C：**Cognitive-behavioral therapy **D：**Multi-component intervention **E：**Enhanced education therapy **F：**Mind-body therapy **G：**Psychotherapy **H：**Enhanced care **I：** Neurostimulation **J：** Relaxation therapy **K：** Supportive therapy **L：** Conventional Exercise **M：** Education

Supplementary Table S7.2.1 – Contribution graph for depression in adolescents

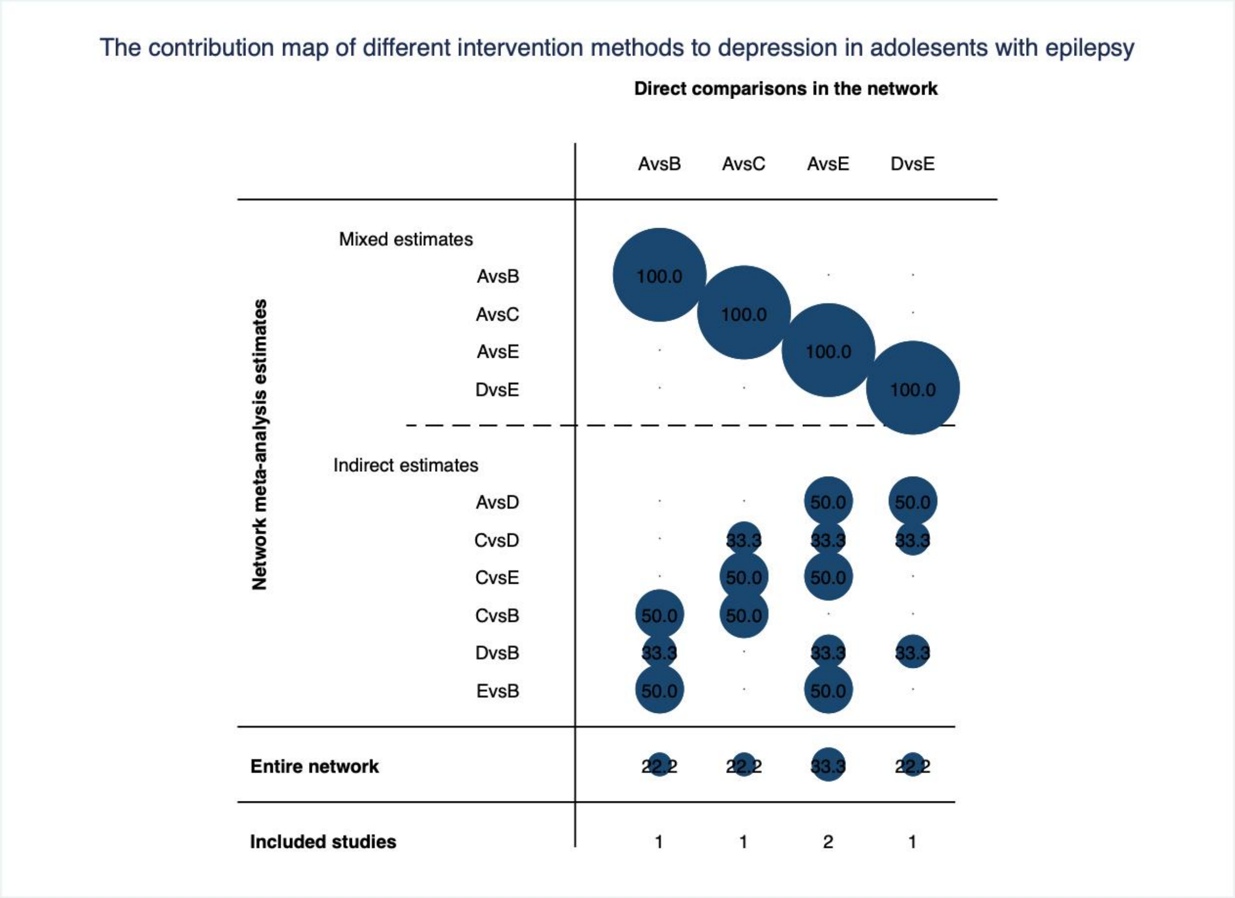

Notes: A： Control group **B：** Enhanced education therapy **C：** Mind-body therapy **D：** Psychotherapy **E：** Enhanced care

Supplementary Table S7.2.2 – Contribution graph for depression in adults

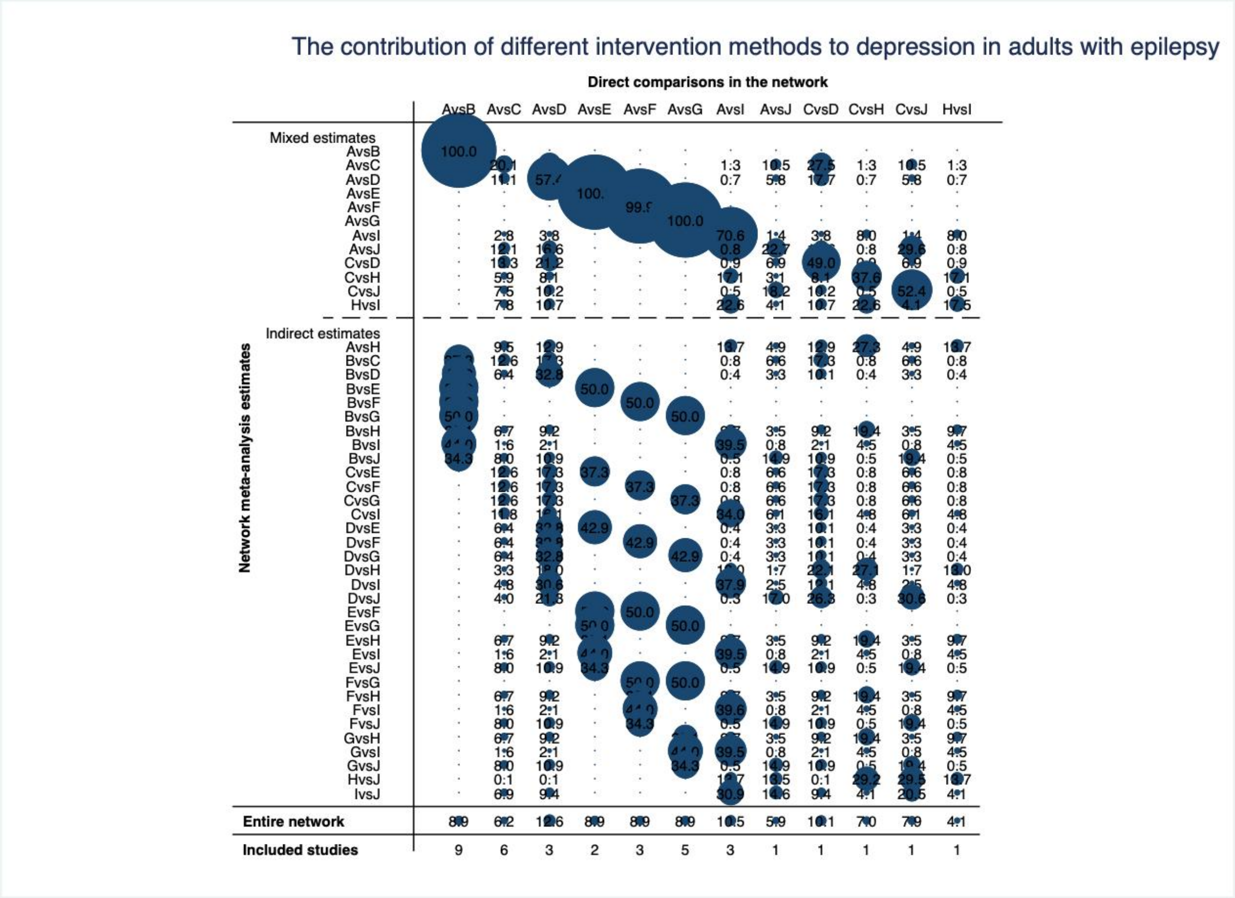

Notes: A：Control group **B：**Self-management **C：**Cognitive-behavioral therapy **D：**Multi-component intervention **E：**Mind-body therapy **F：**Psychotherapy **G：**Neurostimulation **H：**Relaxation therapy

I： Conventional Exercise **J：** Education

Supplementary Table S7.3 – Contribution graph for QoL

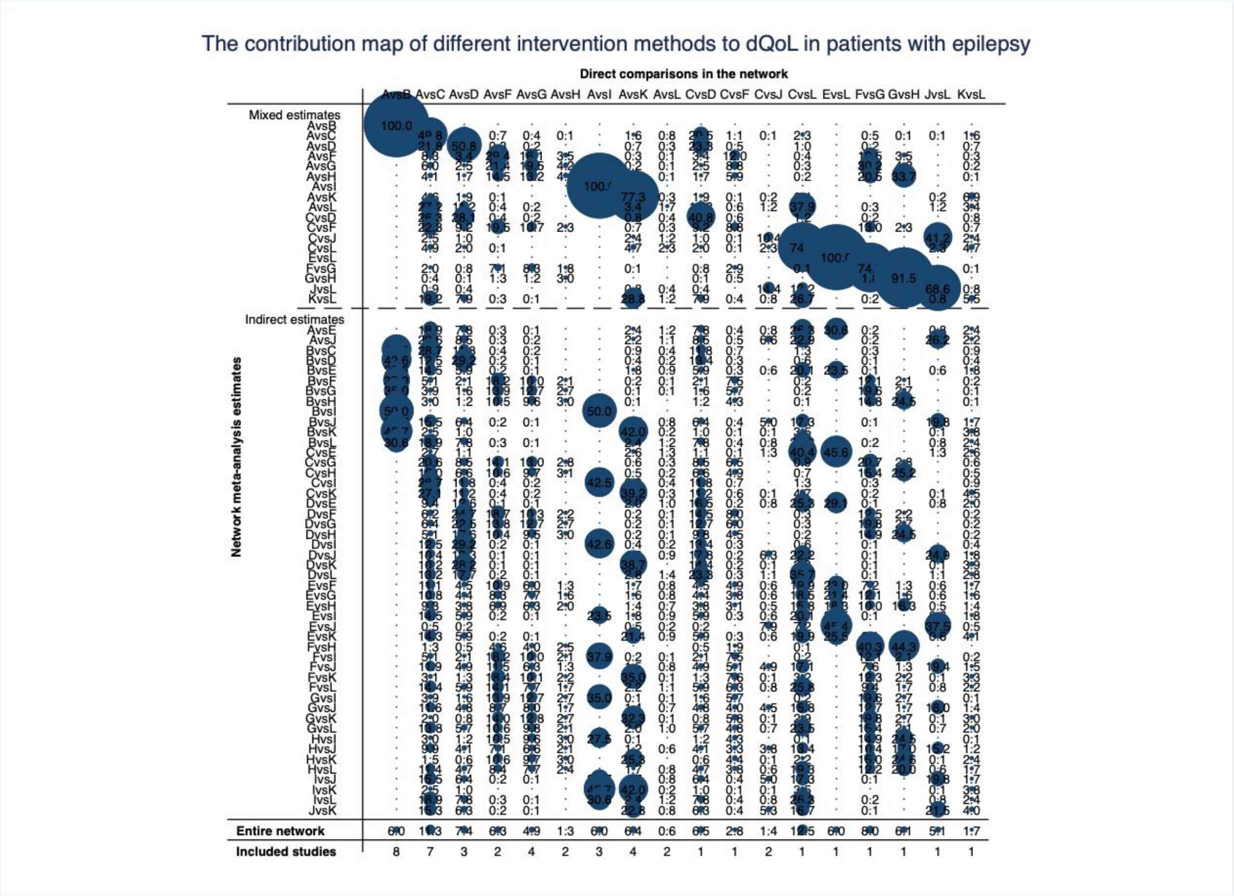

Notes: A：Control group **B：**Self-management **C：**Cognitive-behavioral therapy **D：**Multi-component intervention **E：**Enhanced education therapy **F：**Mind-body therapy **G：**Psychotherapy **H：**Enhanced care **I：** Neurostimulation **J：** Supportive therapy **K：** Conventional Exercise **L：** Education

Supplementary Table S7.3.1 – Contribution graph for QoL in adolescents

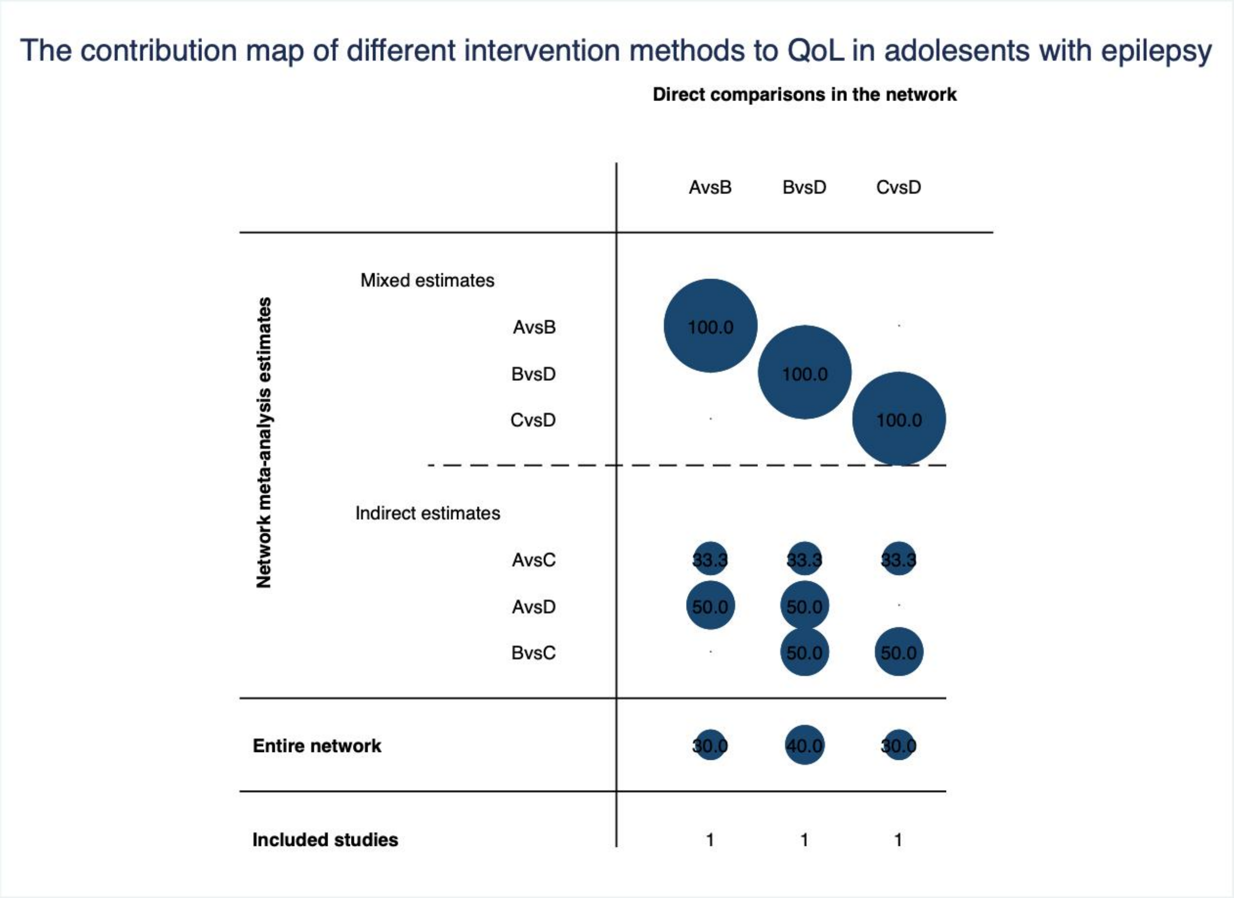

Notes: A： Cognitive-behavioral therapy **B：** Supportive therapy **C：** Enhanced care **D：** Education

Supplementary Table S7.3.2 – Contribution graph for QoL in adults

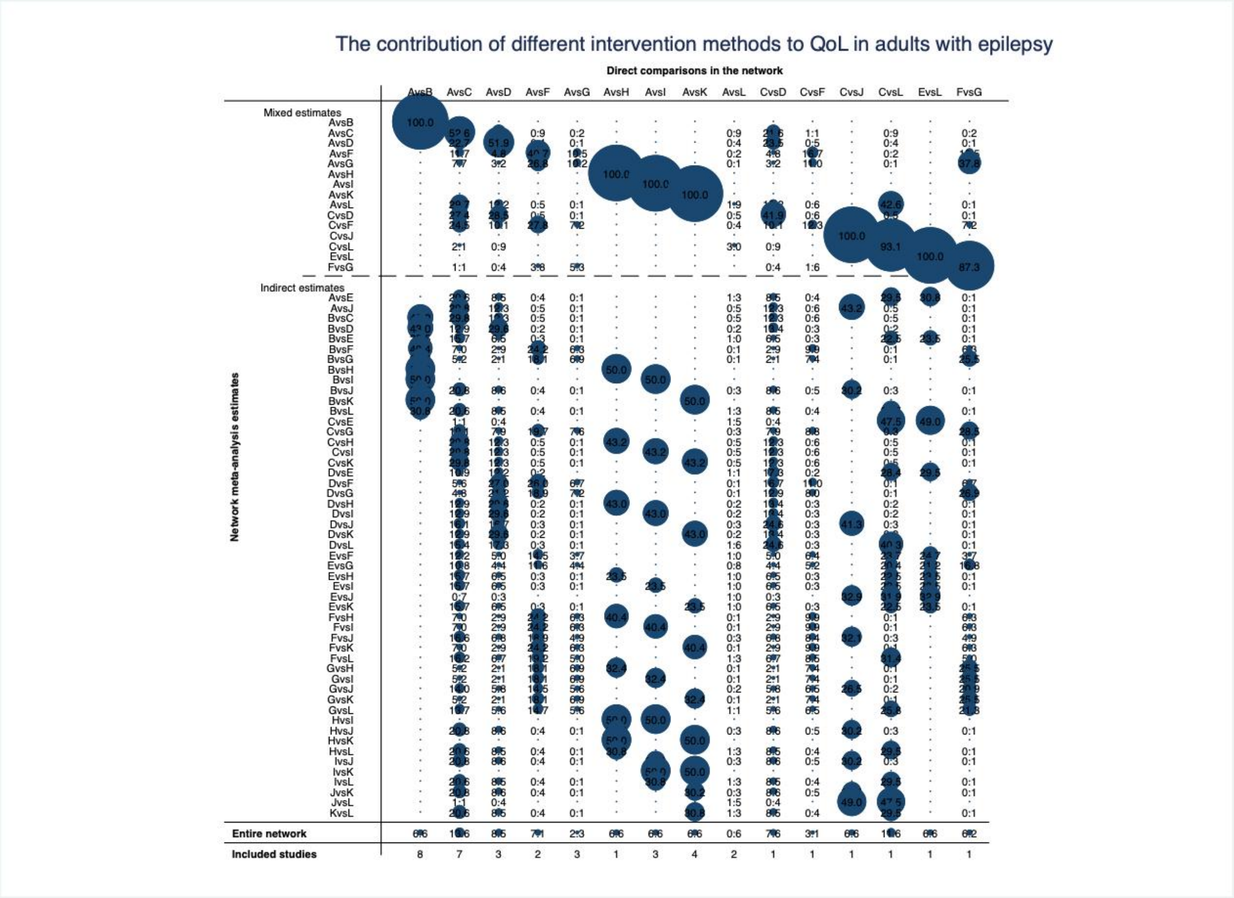

Notes: A： Control group **B：** Self-management **C：** Cognitive-behavioral therapy **D：** Multi-component intervention **E：** Enhanced education therapy **F：** Mind-body therapy **G：** Psychotherapy **H：** Enhanced care **I：** Neurostimulation **J：** Supportive therapy **K：** Conventional Exercise **L：** Education

Supplementary Table S8 – Sensitivity analyses

Supplementary Table S8.1 – Sensitivity analyses for anxiety

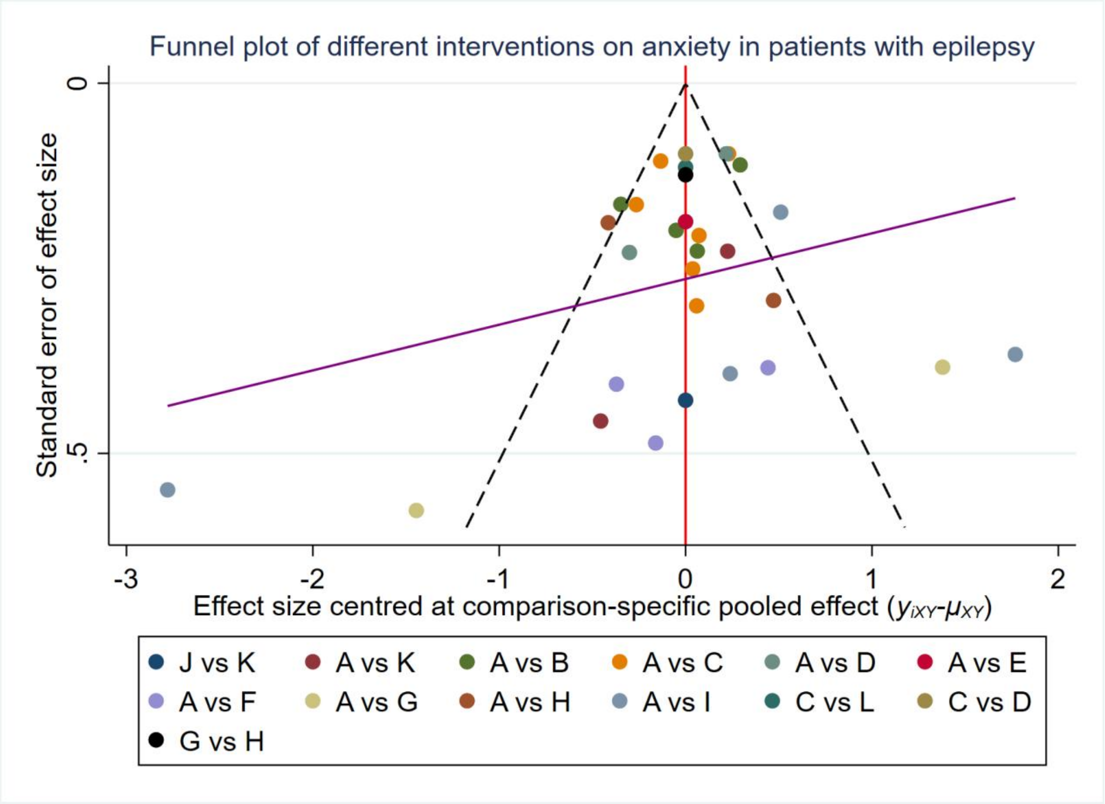

Notes: A：Control group **B**：Self-management **C**：Cognitive-behavioral therapy **D**：Multi-component intervention **E**：Enhanced education therapy **F**：Mind-body therapy **G**：Psychotherapy **H**：Enhanced care **I**：Neurostimulation **J**：Relaxation therapy **K**：Conventional Exercise **L**：Education

Supplementary Table S8.1.1 – Sensitivity analyses for anxiety in adolescents

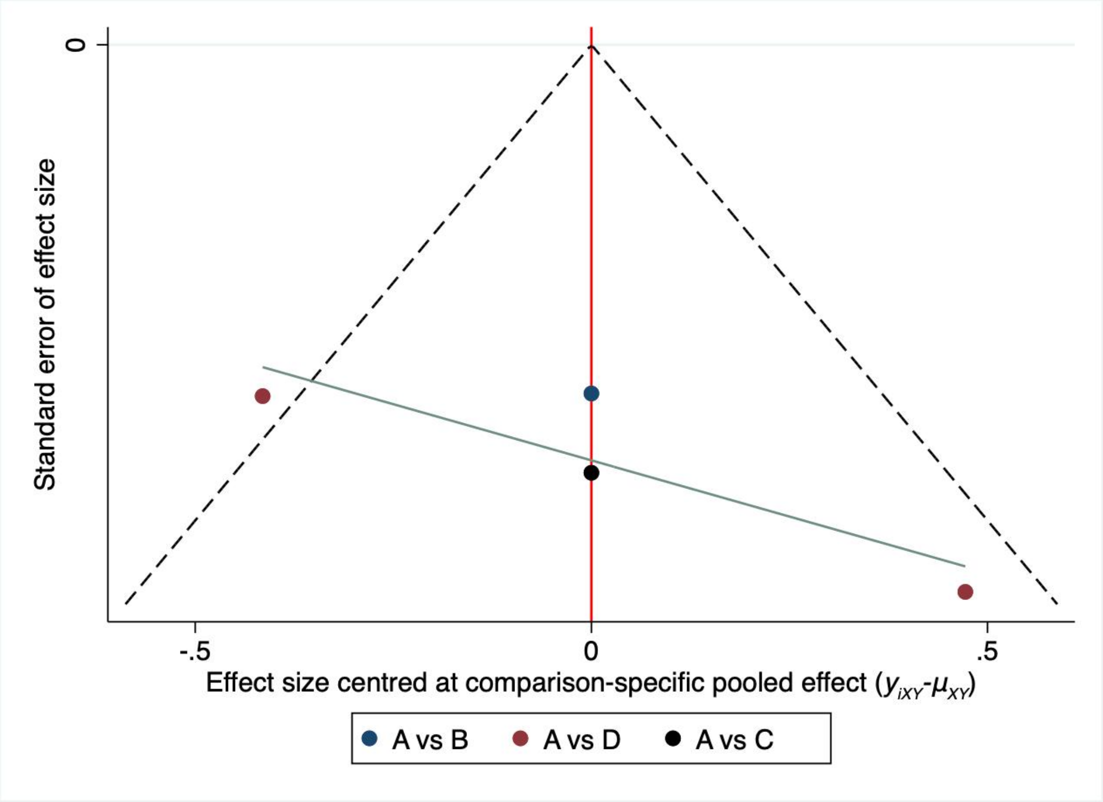

Notes: A： Control group **B：** Enhanced education therapy **C:** Psychotherapy **D：** Enhanced care

Supplementary Table S8.1.2 – Sensitivity analyses for anxiety in adults

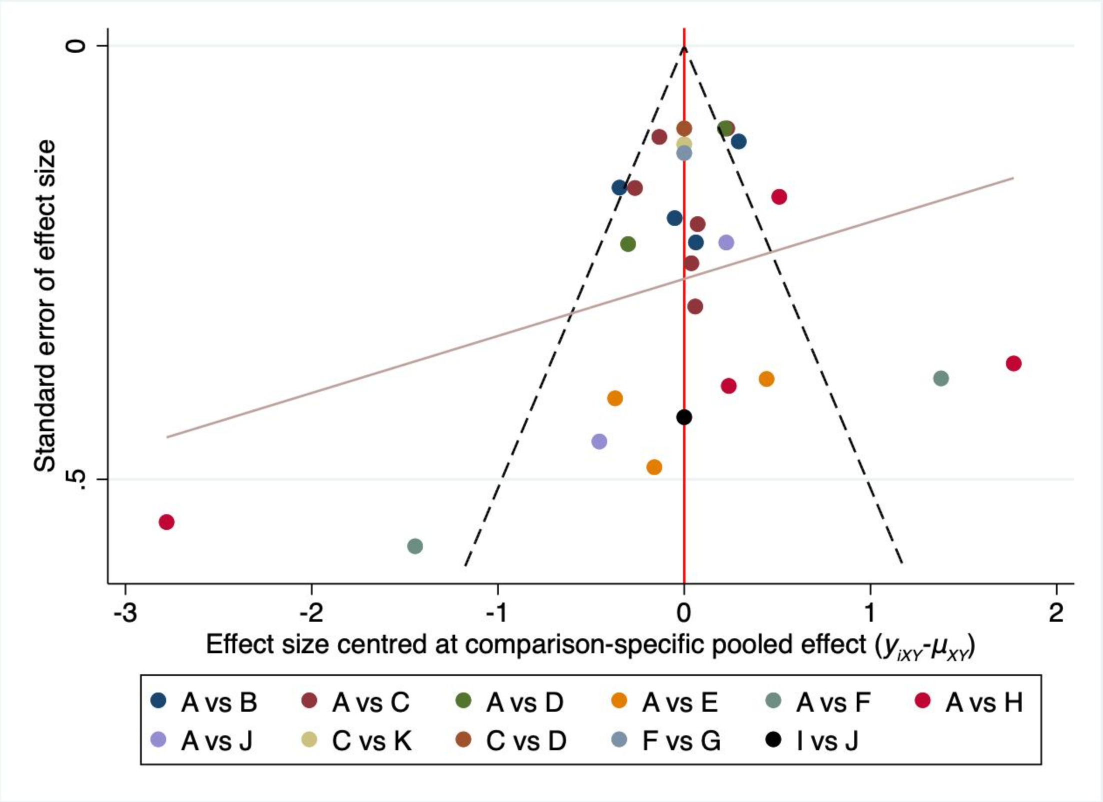

Notes: A: Control group **B**: Self-management **C**: Cognitive-behavioral therapy **D**: Multi-component intervention E: Mind-body therapy F: Psychotherapy G: Enhanced care

H: Neurostimulation I: Relaxation therapy J: Conventional Exercise **K**: Education

Supplementary Table S8.2 – Sensitivity analyses for depression

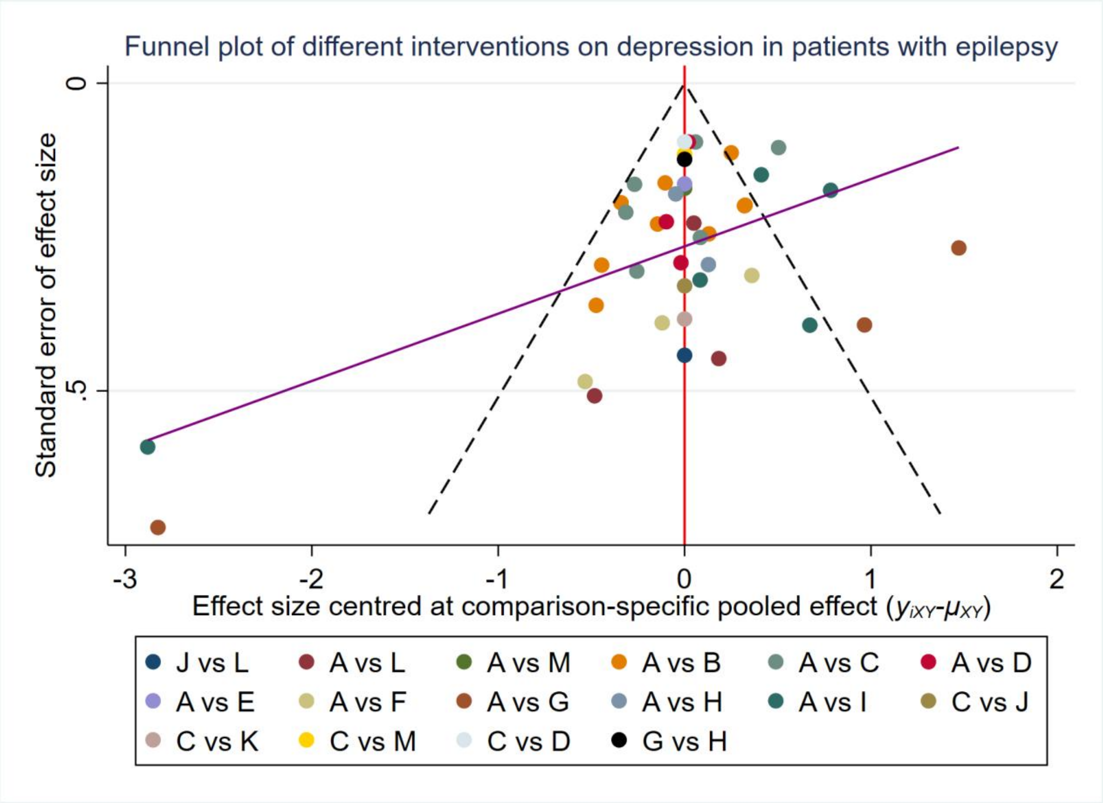

Notes: A： Control group **B：** Self-management **C：** Cognitive-behavioral therapy **D：** Multi-component intervention **E：** Enhanced education therapy **F：** Mind-body therapy **G：** Psychotherapy **H：** Enhanced care **I：** Neurostimulation **J：** Relaxation therapy **K：** Supportive therapy **L：** Conventional Exercise **M：** Education

Supplementary Table S8.2.1 – Sensitivity analyses for depression in adolescents

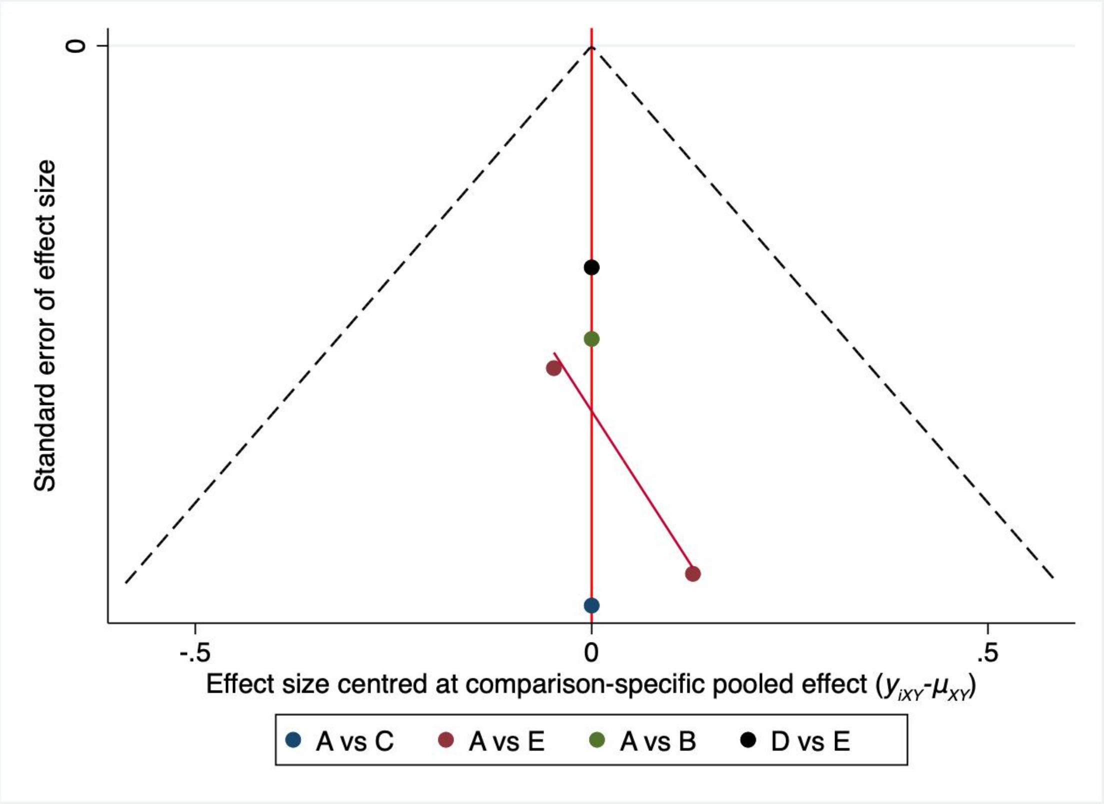

Notes: A： Control group **B**： Enhanced education therapy **C**： Mind-body therapy **D**： Psychotherapy **E**： Enhanced care

Supplementary Table S8.2.2 – Sensitivity analyses for depression in adults

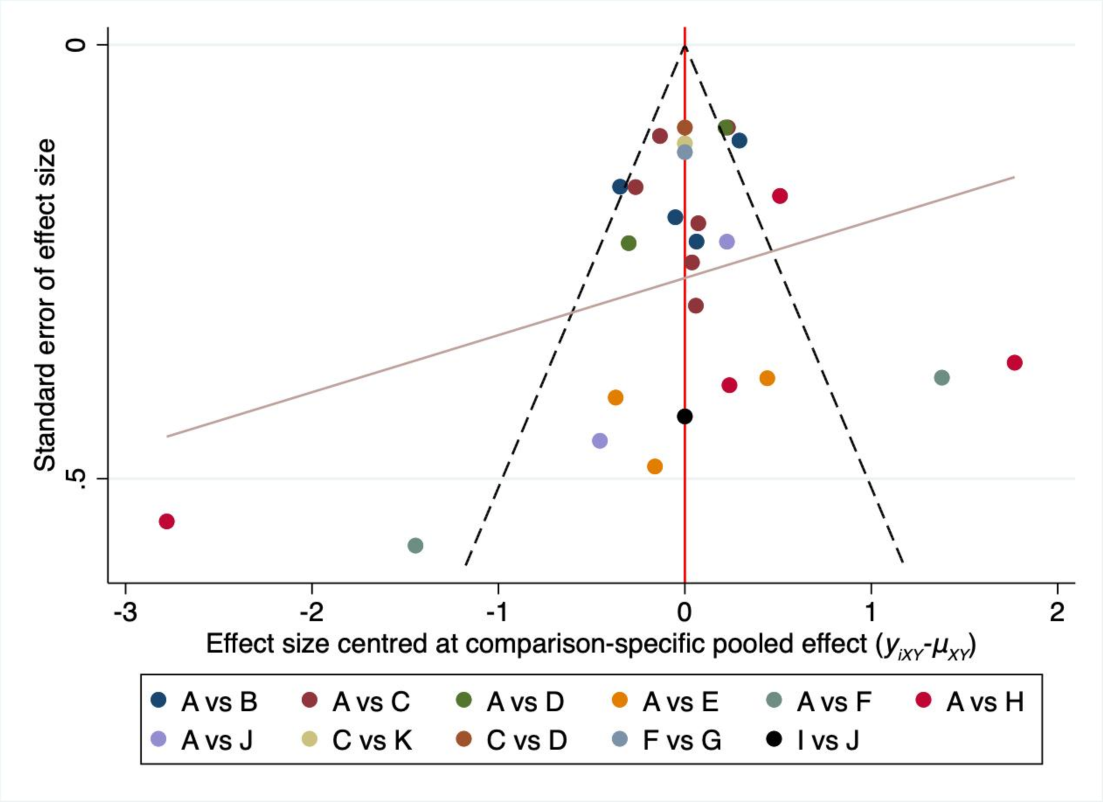

Notes: A： Control group **B：** Self-management **C：** Cognitive-behavioral therapy **D：** Multi-component intervention **E：** Mind-body therapy **F：** Psychotherapy **G：** Neurostimulation **H：** Relaxation therapy **I：** Conventional Exercise **J：** Education

Supplementary Table S8.3 – Sensitivity analyses for QoL

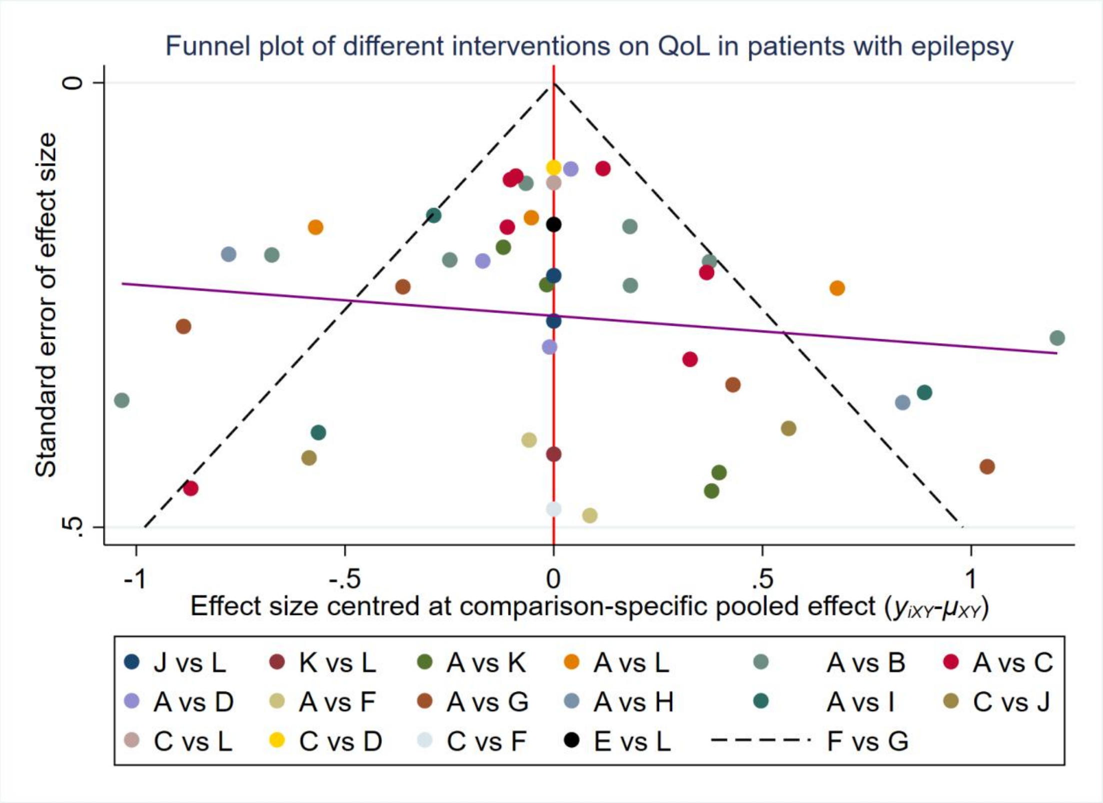

Notes: A: Control group **B:** Self-management **C:** Cognitive-behavioral therapy **D:** Multi-component intervention **E:** Enhanced education therapy **F:** Mind-body therapy **G:** Psychotherapy **H:** Enhanced care **I:** Neurostimulation **J:** Supportive therapy **K:** Conventional Exercise **L:** Education

Supplementary Table S8.3.1 – Sensitivity analyses for QoL in adolescents

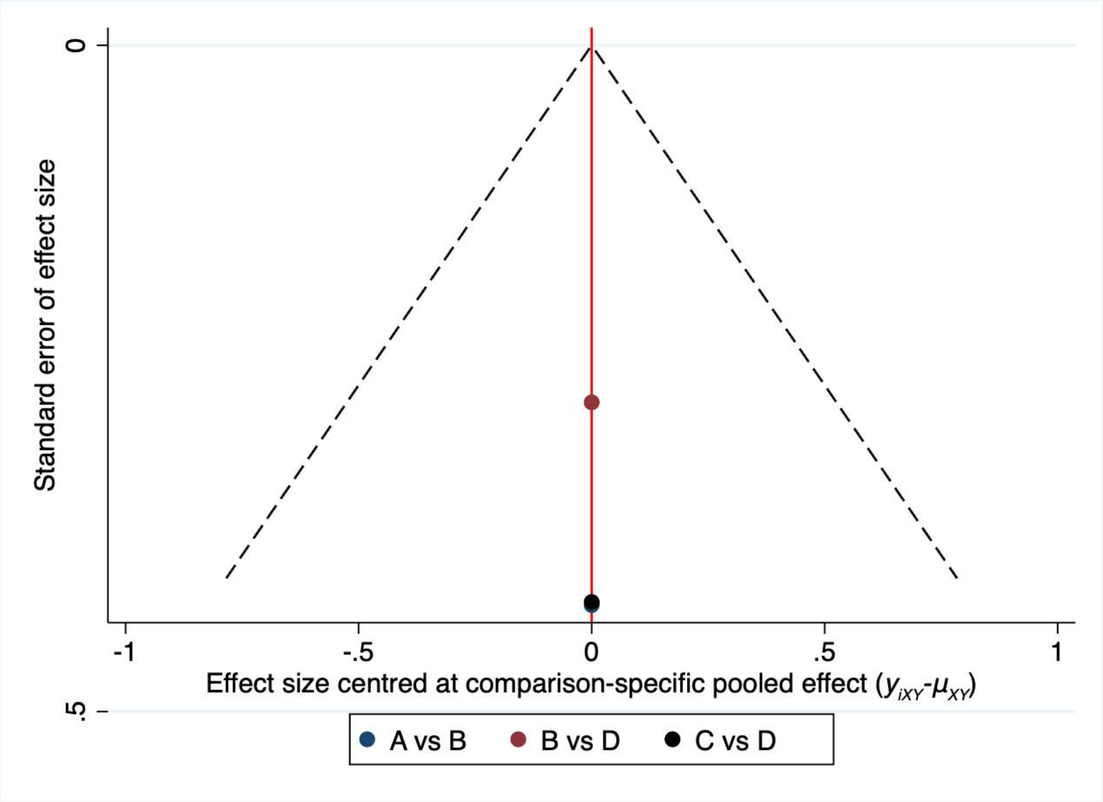

Notes: A： Cognitive-behavioral therapy **B：** Supportive therapy **C：** Enhanced care **D：** Education

Supplementary Table S8.3.2 – Sensitivity analyses for QoL in adults

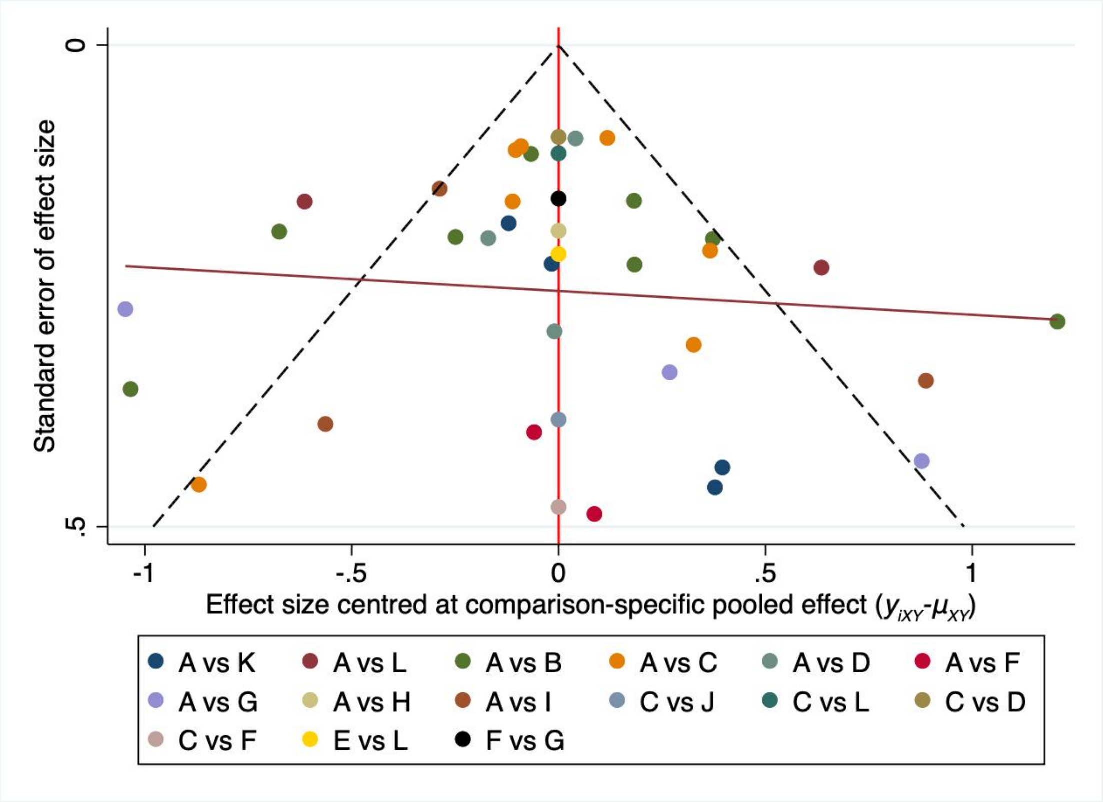

Notes: A： Control group **B**： Self-management **C**： Cognitive-behavioral therapy **D**： Multi-component intervention **E**： Enhanced education therapy **F**： Mind-body therapy **G**： Psychotherapy **H**： Enhanced care **I**： Neurostimulation **J**： Supportive therapy **K**： Conventional Exercise **L**： Education

Supplementary Table S9 – GRADE assessment

Supplementary Table S9.1 – The Grading of Recommendations Assessment, Development and Evaluation (GRADE) assessment for anxiety

Comparison	Risk of Bias	Publication bias	Indirectness	Imprecision	Inconsistency	Confidence rating
CONvsSM	Low risk	No concerns	No concerns	No concerns	No concerns	High
CONvsCBT	High risk	No concerns	No concerns	No concerns	No concerns	Low
CONvsMT	Low risk	No concerns	No concerns	No concerns	No concerns	High
CONvsEET	High risk	No concerns	No concerns	Some concerns	Some concerns	Very low
CONvsMBT	Low risk	No concerns	No concerns	Some concerns	No concerns	Moderate
CONvsPT	Low risk	No concerns	No concerns	Some concerns	Some concerns	Low
CONvsEC	High risk	No concerns	No concerns	Some concerns	No concerns	Low
CONvsNS	High risk	No concerns	No concerns	Some concerns	No concerns	Low
CONvsCE	High risk	No concerns	No concerns	Some concerns	No concerns	Low
CBTvsMT	High risk	No concerns	No concerns	No concerns	No concerns	Moderate
CBTvsEDU	High risk	No concerns	No concerns	Some concerns	No concerns	Low
PTvsEC|	High risk	No concerns	No concerns	Some concerns	No concerns	Moderate
RTvsCE	High risk	No concerns	No concerns	Some concerns	No concerns	Moderate

Risk of bias: high risk: If the study has unclear randomization processes, improper blinding methods, or significant missing data that were not adequately addressed. Low risk: If the study reports a reasonable randomization method, employs appropriate blinding, and has minimal missing data that were handled appropriately.

Imprecision: If the sample size is less than 400, or if the differences between each intervention are very small, the results are considered to be highly imprecise.

Inconsistency: If the predicted intervals for pairwise comparison cross the invalid line alone, they will be downgraded by one level.

Supplementary Table S9.2 – The Grading of Recommendations Assessment, Development and Evaluation (GRADE) assessment for depression

Comparison	Risk of Bias	Publication bias	Indirectness	Imprecision	Inconsistency	Confidence rating
CON VS SM	Low risk	Some concerns	No concerns	No concerns	No concerns	Moderate
CON VS CBT	High risk	Some concerns	No concerns	No concerns	No concerns	Low
CON VS MT	Low risk	Some concerns	No concerns	No concerns	No concerns	Moderate
CON VS EET	High risk	Some concerns	No concerns	Some concerns	No concerns	Very low
CON VS MBT	High risk	Some concerns	No concerns	Some concerns	No concerns	Very low
CON VS PT	High risk	Some concerns	No concerns	Some concerns	No concerns	Very low
CON VS EC	High risk	Some concerns	No concerns	Some concerns	No concerns	Very low
CON VS NS	High risk	Some concerns	No concerns	No concerns	No concerns	Low
CON VS CE	Low risk	Some concerns	No concerns	Some concerns	No concerns	Low
CON VS EDU	High risk	Some concerns	No concerns	Some concerns	No concerns	Low
CBT VS PT	High risk	Some concerns	No concerns	No concerns	No concerns	Low
CBT VS RT	High risk	Some concerns	No concerns	Some concerns	No concerns	Very low
CBT VS ST	High risk	Some concerns	No concerns	Some concerns	No concerns	Very low
CBT VS EDU	High risk	Some concerns	No concerns	Some concerns	No concerns	Very low
PT VS EC	High risk	Some concerns	No concerns	Some concerns	No concerns	Very low
RT VS CE	High risk	Some concerns	No concerns	Some concerns	No concerns	Very low

Risk of bias: high risk: If the study has unclear randomization processes, improper blinding methods, or significant missing data that were not adequately addressed. Low risk: If the study reports a reasonable randomization method, employs appropriate blinding, and has minimal missing data that were handled appropriately.
Imprecision: If the sample size is less than 400, or if the differences between each intervention are very small, the results are considered to be highly imprecise.
Inconsistency: If the predicted intervals for pairwise comparison cross the invalid line alone, they will be downgraded by one level.

Supplementary Table S9.3 – The Grading of Recommendations Assessment, Development and Evaluation (GRADE) assessment for QoL

Comparison	Risk of Bias	Publication bias	Indirectness	Imprecision	Inconsistency	Confidence rating
CONvsSM	Low risk	No concerns	No concerns	No concerns	No concerns	High
CONvsCBT	High risk	No concerns	No concerns	No concerns	Some concerns	Low
CONvsMT	High risk	No concerns	No concerns	No concerns	No concerns	Moderate
CONvsMBT	High risk	No concerns	No concerns	Some concerns	Some concerns	Very low
CONvsPT	High risk	No concerns	No concerns	Some concerns	Some concerns	Very low
CONvsEC	High risk	No concerns	No concerns	Some concerns	Some concerns	Very low
CONvsNS	High risk	No concerns	No concerns	Some concerns	No concerns	Low
CONvsCE	Low risk	No concerns	No concerns	Some concerns	No concerns	Moderate
CONvsEDU	Low risk	No concerns	No concerns	No concerns	No concerns	High
CBTvsMT	High risk	No concerns	No concerns	No concerns	No concerns	Moderate
CBTvsMBT	Low risk	No concerns	No concerns	Some concerns	No concerns	Moderate
CBTvsST	Low risk	No concerns	No concerns	Some concerns	Some concerns	Low
CBTvsEDU	High risk	No concerns	No concerns	Some concerns	No concerns	Low
EETvsEDU	High risk	No concerns	No concerns	Some concerns	No concerns	Low
MBTvsPT	High risk	No concerns	No concerns	Some concerns	No concerns	Low
STvsEDU	High risk	No concerns	No concerns	Some concerns	No concerns	Low
CEvsEDU	Low risk	No concerns	No concerns	Some concerns	No concerns	Moderate

Risk of bias: high risk: If the study has unclear randomization processes, improper blinding methods, or significant missing data that were not adequately addressed. Low risk: If the study reports a reasonable randomization method, employs appropriate blinding, and has minimal missing data that were handled appropriately.
Imprecision: If the sample size is less than 400, or if the differences between each intervention are very small, the results are considered to be highly imprecise.
Inconsistency: If the predicted intervals for pairwise comparison cross the invalid line alone, they will be downgraded by one level.

Supplementary Table S10 – Major modifications for protocal in PROSPERO(CRD420251015149)

Registered	Deviation
Our protocol: The researchers include: Xaoran Luo; Xianming Ding; Junyu Zhang.	Changed: The researchers include: Haoran Luo; Xianming Ding; Junyu Zhang.
